# Genome stability of *Propionibacterium acnes*: a comprehensive study of indels and homopolymeric tracts

**DOI:** 10.1038/srep20662

**Published:** 2016-02-09

**Authors:** Christian F. P. Scholz, Holger Brüggemann, Hans B. Lomholt, Hervé Tettelin, Mogens Kilian

**Affiliations:** 1 Department of Biomedicine, Aarhus University, Denmark; 2 Institute for Genome Sciences, University of Maryland School of Medicine, USA

## Abstract

We present a species-wide comparative analysis of 90 genomes of *Propionibacterium acnes* that represent the known diversity of the species. Our results are augmented by six high-quality genomes and a manual investigation of all gene-sized indels found in the strains. Overall, the order of genes is conserved throughout the species. A public sybil database for easy comparative analysis of the 90 genomes was established. The analysis of indels revealed a total of 66 loci of non-core genes that correlate with phylogenetic clades. No gene was strain-specific in agreement with our conclusion that the *P. acnes* pan-genome is closed. An exhaustive search for homopolymeric tracts (HPTs) identified a total of 54 variable-length HPTs almost exclusively of guanine/cytosines located between genes or affecting the reading frame of genes. The repeat variation was consistent with phylogenetic clades suggesting slow accumulation over time rather than active modification. By transcriptome analysis we demonstrate how an HPT variation can affect the gene expression levels. Selected cases of both indels and HPTs are described. The catalogued data and the public *P. acnes* Sybil database provide a solid foundation for generating hypotheses and facilitate comparative genetic analyses in future *P. acnes* research.

*Propionibacterium acnes*, a Gram-positive rod-shaped bacterium, is a ubiquitous inhabitant of human skin^1^,^2^. Despite the omnipresence on healthy individuals, it is mainly known for its pathogenic potential associated with acne vulgaris^3^,^4^. Other *P. acnes* disease associations include eye- and prosthetic device infections^5^,^6^ and sarcoidosis^7^. Recent *P. acnes* research has focused on population genetics and sequence typing in an attempt to identify sub-populations with differing pathogenic potentials^8^,^9^,^10^,^11^,^12^,^13^. However, interpretation of results is difficult for two main reasons. First, due to its omnipresence, contamination with *P. acnes* is common and hard to exclude^14^. Second, several clones of *P. acnes* inhabit the same niche in each individual^2^,^12^. To overcome these difficulties a better understanding of the pathogenic potential of the individual sub-populations is needed.

One mechanism of differential pathogenic potential may be the ability to turn genes on or off, or to fine-tune their expression according to environmental/ecological circumstances. Single nucleotide repeat sequences, or homopolymeric tracts (HPTs), have been described as signatures of phase-variation in a number of species, e.g. *Neisseria meningitidis*, *Helicobacter pylori* and *Bordetella pertussis*^15^,^16^,^17^. HPTs were detected in the first complete genome of *P. acnes*^18^. Several subsequent studies have noted the presence of HPTs in *P. acnes*^11^,^19^,^20^. In particular, genes encoding putative surface proteins, such as the dermatan-sulphate adhesins, have been shown to be associated with HPTs located in the 5′ untranslated region (UTR) or within the coding sequence^11^. Despite this example a comprehensive genome-wide overview of all HPTs is lacking so far. Moreover, the association of the different repeat sizes with recognized sub-populations and their potential significance for differences in habitat and disease association are unknown.

A second driver of altering the pathogenic potential is gene acquisition or deletion. Several studies revealed inserted genomic islands in *P. acnes* with variable presence across the population^21^,^22^,^23^,^24^. The pan-genome analysis of *P. acnes* by Tomida *et al.* provided an overview of the genetic landscape^25^. However, being the first of its kind it naturally focused on generic observations and left large territories uncharted. Here we present a comprehensive and easily accessible catalogue of all indels, together with an analysis of the HPTs and their distribution across the phylogenetic clades of the species. This may provide clues about the evolution of the species and new information for understanding the dual nature of the association of *P. acnes* with the human host, and may serve as a valuable fundament for generating new hypotheses.

## Results

### Establishment of a genome database of *P. acnes* for comparative studies

Ninety genome sequences were selected that represent the known diversity of *P. acnes*. The genome comparison tool Sybil was employed to create a genome database to facilitate comparative analysis^26^,^27^. A Sybil database is a visual representation of a multiple genome alignment based on sequence homology between proteins encoded by annotated genes. We focused on the identification of indels, HPTs and sites of rearrangement. A link for the freely accessible website for the *P. acnes* Sybil database is provided in the methods section.

### The gene synteny of *P. acnes* is conserved

**Figure 1:**
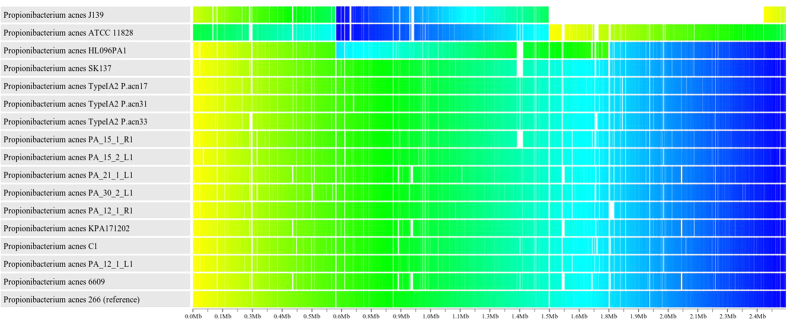
Demonstration of synteny of selected representative genomes. The closed genomes are coloured from start to end with a gradient of yellow through dark blue. Each coloured genome is subsequently mapped to the reference genome (strain 266) and any sharp colour contrast reveals a rearrangement of the genome relatively to the reference sequences (see reference Riley *et al*. 2012^27^ for details). Note, that the J139 genome is not closed, explaining the white region; this is a single large contig clearly showing an inversion relatively to strain 266. Note, that the NCBI sequences of strains J139 and ATCC 11828 are not aligned to start at the origin of replication as indicated by the yellow colour to the right.

The Sybil database allowed us to visually inspect the multiple alignment gene-by-gene. The comparison revealed that the order of genes is conserved, i.e. we found no genes with alternative surrounding genes, unless the gene was flanked by a deletion or an insertion. At the genome scale, large inversions of sequences between two rRNA operons can arise due to the often-complete sequence identity of the respective operons^28^. Such inversions have been described for two clade II strains, ATCC_11828 and HL096PA1^24^,^29^. In strain HL096PA1 the inversion was verified by PCR, and involves the sequence between the first and the second operon^24^, whereas in strain ATCC_11828 it involves the sequence between the first and the third operon, but has not been verified. Our analysis confirmed this observation and, in addition, showed that a similar inversion apparently is present in the *P. acnes* strain J139 (Fig. 1). The fact that these inversions occurred in strains of clade II, and not the other clades, is supported by the disruption of the normal G/C skew pattern in those strains. Despite a large-scale inversion between rRNA operons, the synteny is remarkably conserved, indicative of a highly stable *P. acnes* chromosome.

### Gene-sized indels

Indels of approximately 300 bp or larger may be associated with a gain or loss of function and, therefore, represent genomic footprints that may infer phenotypic differences. The Sybil database was used to spot indels across the different sub-populations of *P. acnes* and to investigate their annotations across the genomes. Different gene prediction tools were used for different *P. acnes* genomes. However, the Sybil database allowed us to identify genes that are missed due to gene prediction biases in some genomes and to detect potential conflicts of annotation on the same genomic region across the genomes.

**Figure 2:**
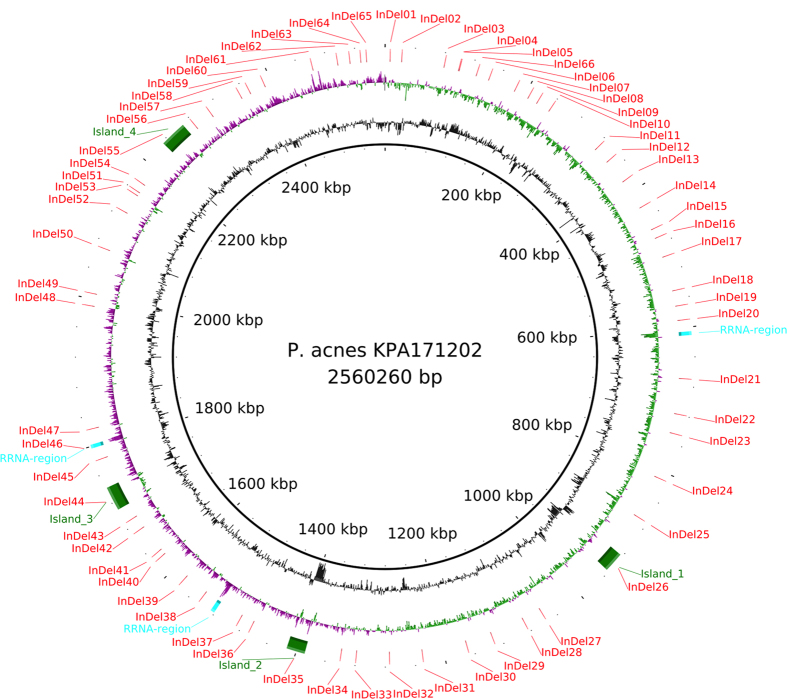
A circular plot showing the distribution of the indel loci. From the center and out the rings describe, coordinates according to the KPA171202 genome, GC content, GC skew (Purple: negative, green: positive) and indels. The three rRNA operons are indicated. Four islands indicated by green bars are as described by Brzuszkiewicz *et al.*^21^.

The analysis revealed a total of 66 sites of larger indels (>300 bp). Their genome distribution is visualised in Fig. 2. The size of indels ranges from small genes to islands encoding up to 32 genes. The supplementary table S1 is an overview catalogue of the 66 loci and the absence/presence of indels across the different clades. The table includes a short description of the putative function of each indel, coordinates according to the KPA171202 genome (NC_006085.1) and a direct link to the *P. acnes* Sybil database that provides a view of the indel with a representative selection of genomes. Previously reported genomic islands have been correctly identified^21^,^24^,^25^,^29^. In addition, our genome-wide reference-free analysis and thorough manual inspection of the data identified new indels and provides easy access to the relevant information.

### Indel analysis reveals overall lack of strain-specific genes

Recently Minegishi *et al.* described an arsenic resistance island of 18.8 kb uniquely defining the *P. acnes* strain C1^23^. As mentioned by the authors, the island is found also in the strain *Propionibacterium* sp. 5U42AFAA, a strain that we previously demonstrated to be *P. acnes*^12^. In the present analysis we found the resistance island also in PA_15_2_L1, like 5U42AFAA a *P. acnes* strain of a particular lineage of clade IA_1_. Although the island is not confined to C1, this particular strain constitutes a monophyletic clade in a phylogenetic tree based on 1262 core genes^23^. The C1 strain is also unique among the genomes investigated in this study by having several copies of a unique insertion sequence distributed across the genome: 14 loci are present in strain C1 that encode the same transposase element. Two transposase elements flank the arsenic resistance island in C1 and PA_15_2_L1. However, in contrast to strain C1 no replications of the elements are found elsewhere in the PA_15_2_L1 genome. Except for the transposase loci of strain C1, we only found three other strain-specific indels i.e. deletions that uniquely define a single strain (Supplementary table S1, indels 40, 46 and 47). Indel_40 is a deletion of two genes in the type II strain HL001PA1. The genes encode an ABC transporter (substrate-binding domain-containing protein) and actinobacterial surface-anchored domain protein. Indel_46 is a 25 kb deletion in strain PA_12_1_R1 including the DNA polymerase III alpha subunit, a mur ligase and lysozyme M1 (Supplementary table S1). Finally, indel_47 is a 13 kb deletion in strain PA_30_2_L1. The region encodes transport proteins and proteins with von Willebrand factor domains. These rare cases provide an opportunity to investigate the biological effects of these particular indels. The lack of strain-specific genes indicates that the inclusion of new genes is a rare event in *P. acnes* which is consistent with the current limitation to genetically modify the organism. Moreover, it suggests that a newly sequenced strain of the same phylogenetic clade would show the same indel pattern.

### Prophage-like regions in the *P. acnes* genomes

Indel_44 constitutes an insertion of 30 kb in all clade IB strains (indel_44 in Sybil). The island has been previously described as a cryptic prophage based on the homology to phage proteins^21^. The island is inserted into the gene encoding a type II restriction enzyme, giving rise to two pseudogenes (PPA1578 and PPA1614). Interestingly, the gene is also deleted together with four functionally related genes (DEAD/DEAH box helicase family proteins) in clade II strains and moreover, a smaller deletion has removed the gene from the C1 strain. Only a subset of the clade IA strains (SLST clades A, B, E, G and F4-F6) has an intact reading frame encoding 1541 aa. Notably, there are two different frame-shift mutations within the clades, introducing two different stop codons. Thus, five different events independently have inactivated the restriction system. This indicates that a recent change in the ecological niche caused a significant disadvantage for strains that express the restriction-modification system.

Clade III strains have an insertion of approximately 20 kb encoding phage related genes (see indel_32). A blastn search revealed that parts of the region are found in other species as well as in the *P. acnes* clade IB (indel 44 described above). The best matches were found in *P. avidum*, *P. acnes* and species of *Corynebacterium* and *Mobiluncus*. The two strongest hits (*P. avidum* and *Mobiluncus curtisii subsp. curtisii*) cover 3.1 kb of the 20 kb insertion, with an identity of 71% at the nucleotide level.

Despite the omnipresence of active phages that can infect *P. acnes*^30^, phage-like regions are scarce in *P. acnes* genomes. Our data thus supports the assumption that phage genome insertion, as part of a temperate phage life cycle, is rare or does not take place at all in this species, since prophage insertions would have left their genomic footprints. Instead, a pseudolysogenic life cycle was suggested for present-day, active phages specific for *P. acnes*, where the phage genome can be found as an extrachromosomal element^31^.

### Indels encoding bacteriocins and other antimicrobial compounds in *P. acnes*

**Figure 3:**
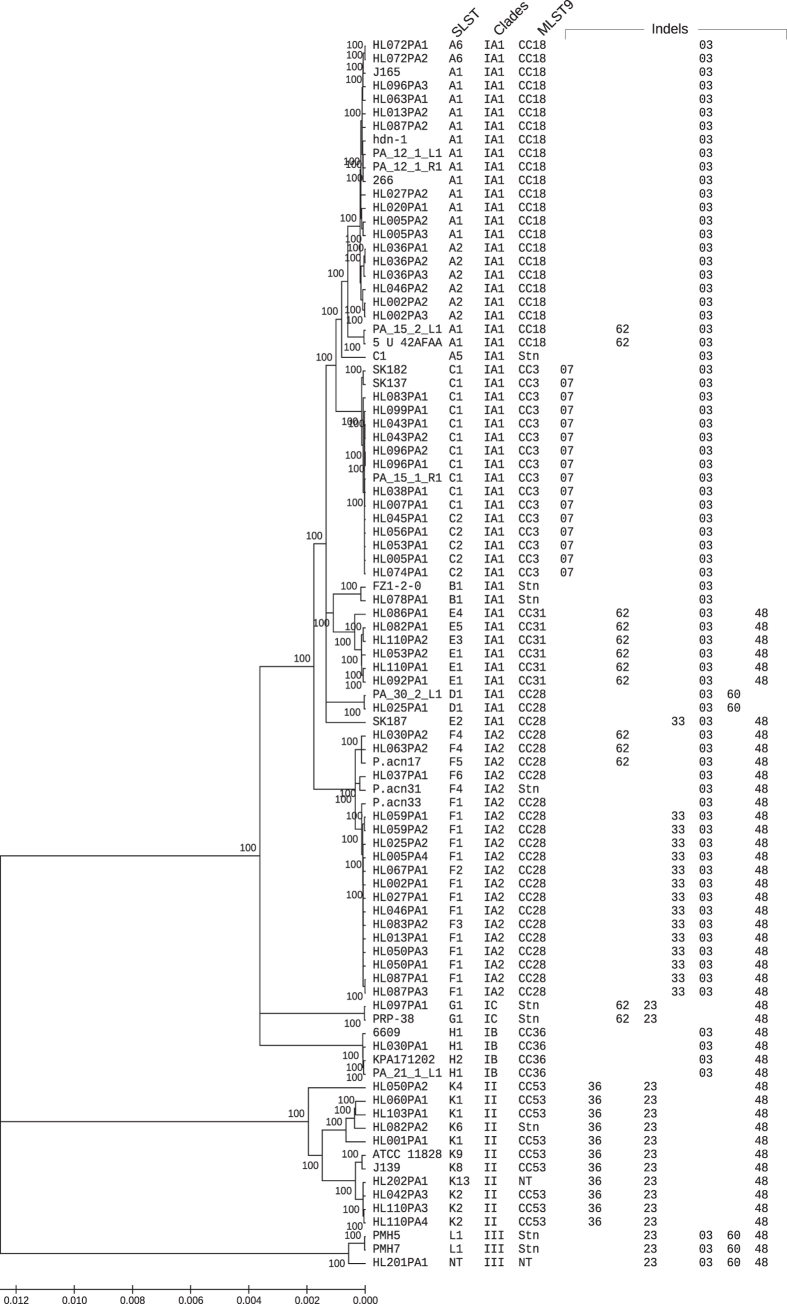
Phylogenetic tree constructed on 1.5 Mb shared sequences in Mega v5.05 with the minimum-evolution algorithm set to complete deletion and 500 boot-strap replications (showing only values of 100%). The figure provides a conversion table between the major phylogenetic clades, the SLST scheme and the MLST9 scheme, as well as a distribution overview of a selected range of indels. Strains not typed because of lack of data or Sanger verification is denoted “NT” and singletons “Stn”.

Our analysis revealed a range of indels encoding putative antimicrobial compounds that are variably present between the phylogenetic clades (Fig. 3). Conceivably, these are important for the strains to combat each other or other species encountered in the same habitat.

An island (indel_26) in clade IB strains and strain SK187 includes a gene cluster encoding a thiopeptide that was previously described^21^. We also found the island in all clade III strains. The annotation suggests that the cluster encodes a class I bacteriocin of the thiopeptide type. Furthermore, we found this locus to be a hotspot for insertions/deletions (see description of indel_26 below).

Another island (indel_25) is composed of 10–11 genes that might encode a class IIb bacteriocin related to lactococcin (PFAM: PPA0802). This island is absent in clade III strains. Interestingly, only the gene encoding a lactococcin 972-domain protein is deleted from clade II strains, leaving the remaining part of the island intact. The other functions encoded by this island are transport functions possibly specific for iron. Thus, this island confers multiple fitness functions. The two independent losses of the bacteriocin gene suggest a common environmental challenges of clade II and III strains.

Indel_07 constitutes a 20 kb island present in all SLST-C strains. The island contains genes related to the synthesis of a streptolysin-like protein. The island has been previously described^21^,^24^.

Another putative bacteriocin is encoded in an island of three genes (indel_60). The genes encode two ABC transporters and a thiazolylpeptide-type bacteriocin (UniProt). The co-insertion of these genes suggests that they are functionally linked. The island is only present in clade I strains, and two genes upstream of the island the same strains have a deletion of a gene encoding a putative dihydrodipicolinate synthase, which belongs to the family of lyases, specifically the amine-lyases that cleave carbon-nitrogen bonds. Interestingly, there is one exception to this pattern. The strains of the SLST-D clade (part of clade IA_1_) are identical to the clade III strains in this region of the genome (Fig. 3).

Indel_62 describes a 25 kb island of 21 genes inserted in genomes belonging to clade IC, SLST-E, a subgroup of SLST-F and a subgroup of SLST-A (Fig. 3). Interestingly, we found the island outside *P. acnes* in a strain closely related to *P. avidum* (GenBank: ATFM00000000.1). The island encodes genes for the biosynthesis of a lantibiotic substance, regulatory function and transporters. It is conceivable that this island harbours a complete gene-set for producing and exporting an antimicrobial substance, thereby providing an advantage for the producer strains in their habitats.

### Bacitracin resistance

Bacitracin, a mixture of cyclic peptides, is produced by some isolates of *Bacillus subtilis*, and has been widely used as a topical antibiotic to treat and prevent skin infections. We found an island of six genes (indel_36) present in all clade II strains that harbours a gene related to resistance to bacitracin. In addition, the island encodes a transcriptional regulator of the PadR family as well as four hypothetical proteins.

### The complex locus of indel_26

The indels of *P. acnes* are usually simple and can be explained by one or two genetic events. However, one site seems to be a hotspot for insertions. The locus of indel 26 is shaped by a complex mixture of insertions and/or deletions. All three clade III genomes have two contig breaks in this locus; thus the reconstitution of this locus in clade III strains remains speculative. Clade II strains contain the lowest number of genes in this locus, all of which are conserved in other clades. We used the clade II genome as reference to describe the various indels that constitute the locus. The first insertion is an island of genes encoding ABC transporters and hypothetical proteins (PPA0840-PPA0845) present in all clade I strains. The next island (PPA0846-PPA0856), which is composed of genes annotated as TraA-like proteins, DNA processing/helicase and hypothetical proteins, is found only in clade IB strains and the clade IA_1_ strain SK187. TraA refers to conjugal transfer protein TraA of *Escherichia coli*^32^, indicating that this region is or has been part of a mobile genetic element. The third indel is the already mentioned thiopeptide island (PPA0857-PPA0869), which is present in clades IB, III and strain SK187. The fourth indel consisting of approximately seven genes (PAZ_c09000-PAZ_c09060), is only found in clade IA and IC strains and encodes a putative two-component system and a response regulator of the LuxR family, likely to affect the expression of yet unidentified genes. Another insertion is a two-gene indel found in all clade I and III strains, encoding a “GntR family transcriptional regulator” and a hypothetical protein (PPA0875 and PPA0873). Downstream of the previous indel clade IA and IB strains harbour a gene encoding an UvrABC system protein A (PPA0877), and clades IC and III have five genes encoding a type I restriction-modification system (HMPREF9344_02055-HMPREF9344_02061). This region is unique in being the only locus with such a complex genetic composition.

### Indel encoding a dimethyl sulfoxide reductase is lacking in clade III genomes

Indel_19, which is missing in all clade III strains, contains genes encoding all three subunits of a dimethyl sulfoxide reductase. This was first mentioned by Tomida *et al.*^25^. This deletion suggests that clade III strains are unable to carry out anaerobic respiration with DMSO as a terminal electron acceptor. This may affect their growth rate under anaerobic conditions^33^.

### Iron uptake may be impaired in clade II and IC strains

Iron availability is limited in the human host and thus, low iron concentration often becomes a bottleneck for bacterial proliferation. Thus, bacteria have evolved iron uptake systems with high affinity. Indel_03, present in strains of clades IA, IB and III, harbours genes (PPA0075-PPA0078) involved in iron uptake and energy coupling to transport according to a StringDB search^34^. The genes form part of a larger iron uptake network (see supplementary Fig. S1). This indel is deleted in clade IC and II strains, which may affect their survival under low iron conditions.

### CRISPR/cas system is partly lost in clade III strains

The CRISPR/cas system has been previously identified in clade II strains^22^,^25^. Our analysis confirms the presence in all clade II strains. However, as displayed in indel_58 (see Sybil indel_58), part of the system is also present in clade III strains. As clade III strains lack the genes for Cas3, CasA, Cse2 and CasC, the system apparently is not functional in clade III strains. However, the remains provide interesting information on the evolution of the species. The CRISPR/cas system is also not found in the two included “*P. humerusii*” strains, which suggests that either the system has been lost independently in different lineages during adaptation to the human host or has been acquired in a common ancestor of clade II and III and then partly lost again in clade III.

### Hyaluronate lyase was acquired twice in *P. acnes*

Indel_14 is briefly mentioned by Brzuszkiewicz *et al.*^21^. Here we have more genomes available to describe the indel in more detail. The hyaluronate lyase gene is present in all strains except for clade III strains. Interestingly, there are two major alleles of this gene, one found in the clades IB and II, and one found in the clades IA and IC. Indeed, the indel_14 constitutes two different insertions at the same locus both including a gene encoding a hyaluronate lyase that exhibits 73% protein identity between the clades IB/II and IA/IC. The insertion in clades IB/II includes four hypothetical genes, and the insertion in IA/IC encodes a 4-phosphoerythronate dehydrogenase, a putative oxidoreductase, an AP endonuclease, a glycosyl hydrolase, a pyridine nucleotide-disulfide oxidoreductase and a 3-ketoacyl-(acyl-carrier-protein) reductase.

### The delusive inversion

**Figure 4:**
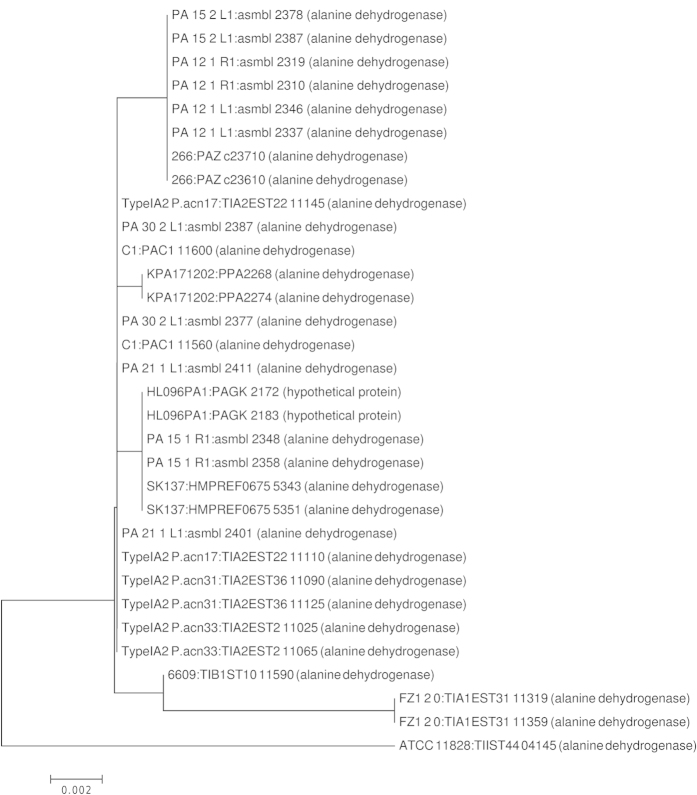
A minimum-evolution tree of the two copies of alanine dehydrogenase. Note, that most strains have identical copies.

At first glance indel_61 looks like a simple inversion; however, the direction of the reading frames reveals it to be a more complex rearrangement. Interestingly, two genes both coding for an alanine dehydrogenase and both located on the same strand flank the site. The two copies are for most strains identical in sequence (Fig. 4). Sixty six draft genomes that are not closed had a small contig spanning only the rearrangement site. Thus, it is possible that the rearrangement and contig-break is a result of an assembly error occurring because of the repeated sequence.

## Homopolymeric tracts

**Figure 5:**
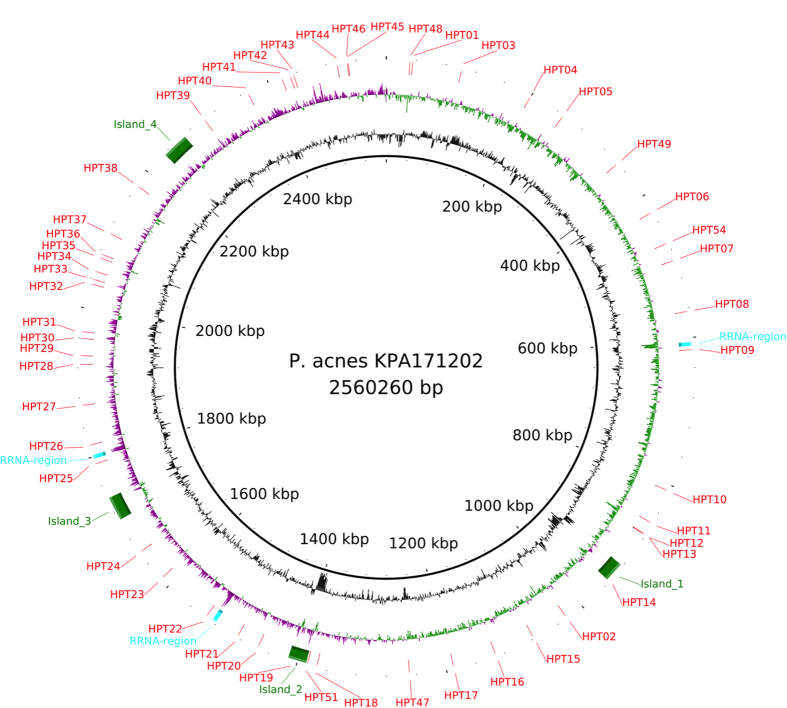
A circular plot showing the distribution of the HPT loci. From the center and out the rings describe; coordinates according to the KPA171202 genome, GC content, GC skew (Purple: negative, green: positive) and HPTs. The three rRNA operons are indicated. Four islands indicated by green bars are as described by Brzuszkiewicz *et al.*^21^.

In total, 54 polymorphic HPTs were found across 90 genomes spanning the known diversity of *P. acnes*. Notably, 96% of the HPTs were repeats of guanines (G) or cytosines (C), and only two were adenine (A) or thymine (T) repeats. However, six of the G/C HPTs were joined to a A/T repeat and another two were connected to a GA or CT repeat section (i.e. GAGAGGGG or TTTTCCCC). Thirty nine out of 54 (72.2%) of the HPTs were found between annotated genes, and the rest within open reading frames. The HPTs were unevenly distributed over the genomes (Fig. 5).

A major concern with single-nucleotide-repeats in high-throughput sequencing data is that the length-variation of repeats is an artefact due to sequencing errors. For this reason we validated the six new genome sequences using Sanger sequencing in all single nucleotide repeats loci. These high-quality genomes represent different lineages of clade I. We observed two kinds of clade-specific patterns in the distribution of the HPT repeat-lengths (see supplementary table S2). First, in most cases, strains of the same SLST clade had the same HPT repeat-length (Supplementary table S2). Second, the variation in the repeat-length for several HPTs was uniquely confined to a particular clade (e. g. HPT_03). Evidently, for most HPTs the signal of common decent is stronger than what can be concealed by the noise of sequencing errors. However, the few HPTs (HPT_01, 13, 16, 19, 33, 40, 41, 52) that did not show any clade-specific patterns all had low variation across clades, with sporadic outliers (Supplementary table S2). Some of these may be due to sequencing errors, as only three of these HPTs (HPT_16, 33, 52) have confirmed variable lengths in the six high-quality genomes.

The HPTs fall into three different categories: intragenic HPTs that cause potential frameshifts, intergenic HPTs that potentially affect promoters, UTRs or regulatory sequences, and HPTs that are associated with genomic rearrangement sites. In the following we will highlight examples of each category. The full list of HPTs is given in supplementary tables S2 and S3.

### Variation of intragenic HPTs can lead to frameshifts

Variable repeats within open reading frames conceivably affect the reading frame and may lead to truncated proteins. The sequence containing the HPT_06 is only found in clade IB and II strains. There is considerable variation in the length of the HPT (6–12 nt) and in some strains, it causes truncation of the encoded protein, annotated as putative oxidoreductase (PPA0378) HPT_07 is located within a gene encoding a hypothetical protein (PPA0448) and variants of HPT_07 confer a loss of function for most clade I strains. The most extensive variation in the length of HPT_07 (8–13 nt) is seen among the SLST-A strains. The HPT_10 is in most strains a 6-nt repeat, but in clade II strains there is some variation. The singleton strain SK187 harbours a longer repeat, and there is some minor variation in a few clade I strains. HPT_10 is located within a gene encoding a putative hydrolase (PPA0735). Interestingly, in the strain C1 this gene is split by insertion of a genomic island encoding arsenic resistance (Indel_05). HPT_21 marks the location of two hypothetical genes upstream of a gene encoding the small subunit of a 3-isopropylmalate dehydratase. In an alignment with “*P. humerusii”* it becomes conceivable that the two hypothetical proteins are the deteriorated remains of the large subunit of a 3-isopropylmalate dehydratase, with which they share homology (See supplement Fig. S2 or go to Sybil). For a complete list of HPTs that are located within annotated CDSs see supplementary table S3.

### Intergenic HPTs

**Figure 6:**
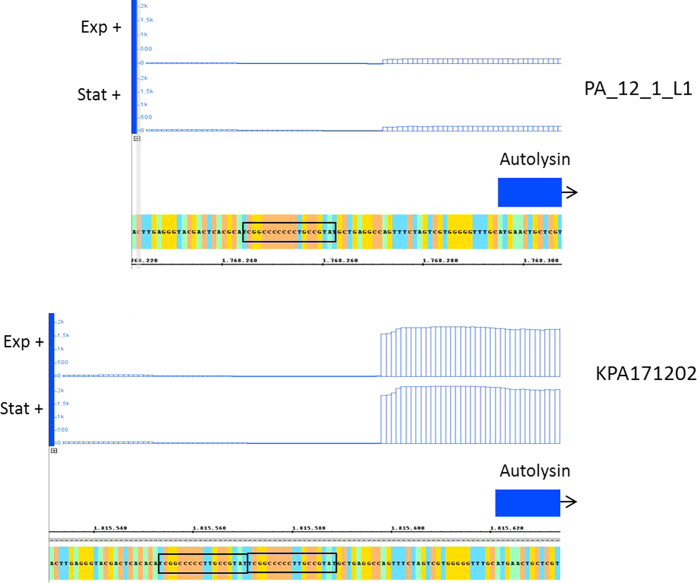
Gene expression difference associated with variation in HPT_26 The graph depicts the results of transcriptome analysis in the vicinity of HPT_26, which is located upstream of a gene encoding a putative autolysin (PPA1662 in strain KPA171202). The number of sequence reads in this area (after normalization) is shown, highlighting that mRNA levels of the autolysin gene is 10-fold increased in strain KPA171202 compared to PA_12_1_L1. HPT_26 is part of an 18 nt element duplicated in strain KPA171202 and located within the promoter of the autolysin gene.

A majority of the identified HPTs (72.2%) resides between annotated genes. Three of these intergenic HPTs are located between the 3′-ends of two genes, thus may overlap with a sequence that harbours a transcription terminator. Terminators are often associated with a stretch of repeating nucleotides; Rho-dependent terminators often contain cytosine-rich sequences on the mRNA (Rho utilization sites), whereas Rho-independent ones often contain poly-T tracts. The majority of HPTs (66.6%) are located upstream of the 5′-end of a gene. Such HPTs could be located in the 5′UTR, in the promoter region or in an upstream region with possible regulatory function. A polymorphic HPT in such regions thus could affect expression efficiency in individual strains. As a proof of concept, and as an inspiration for future work, we compared the transcriptome of two strains belonging to different clades (i.e. IA (PA_12_1_L1) and IB (KPA171202)), and looked at the expression profiles of genes that harbour HPTs with different repeat-lengths in their upstream regions. The presented transcriptome data is a small preliminary excerpt of a larger dataset that will be analysed and presented in a future report. The transcriptional profile of the genomic region around HPT_26 is shown in Fig. 6. HPT_26 can be found upstream of the gene PPA1662 that encodes the putative autolysin (lysozyme M1) of *P. acnes*. Previous studies have shown that a clade IA strain, in contrast to a clade IB strain, secretes decreased levels of PPA1662^19^. In agreement, microarray-based experiments showed down-regulation of PPA1662 in a clade IA strain compared to a clade IB strain^21^. This is confirmed by our transcriptome analysis: we found that expression of PPA1662 is approximately 10-fold reduced in strain PA_12_1_L1 compared to KPA171202 in the exponential growth phase. The possible explanation for this transcriptional difference is linked to the variability of HPT_26 (Fig. 6). This HPT contains a stretch of 5 and 7 cytosines in strains KPA171202 and PA_12_1_L1, respectively. In contrast to strain PA_12_1_L1, the C stretch in strain KPA171202 is part of a region of 18 nucleotides that is directly repeated. As the transcriptome data revealed the transcriptional start site of PPA1662 (23 nucleotides upstream of the start codon), we can conclude that the C-stretch containing direct repeat is located within the promoter region, i.e. overlapping with the −10 element. The biological significance of different autolysin levels in type IA and IB strains has to be further investigated. In *Streptococcus pneumoniae* the autolysin is an important factor contributing to its virulence^35^. Another example is HPT_37, which is located upstream of PPA1943, encoding a putative ubiquinone/menaquinone biosynthesis C-methylase (UbiE) that is required for biosynthesis of ubiquinone (coenzyme Q) or menaquinone (vitamin K2). These are essential isoprenoid quinone components of the respiratory electron transport chain. Here the transcriptome profile reveals a 2-fold reduction of expression of PPA1943 in strain KPA171202 compared to PA_12_1_L1 in the exponential growth phase. As the transcriptional and translation start site is identical (i.e. leaderless transcript), HPT_37 must be located between the −10 and −35 elements of the promoter. HPT_37 contains a stretch of 5 and 7 cytosines in strains KPA171202 and PA_12_1_L1, respectively, thus affecting the spacer length of the promoter, which might explain the observed expression difference.

### HPTs marking rearrangement sites

HPT_15 (Indel_28) is located at a position that marks a rearrangement spot. At this position an indel is present. Clade II strains harbour an insertion of two genes at this position, encoding a glucoside hydrolase family protein and a beta-glucosidase (see Sybil – HPT 15). It seems likely that the HPT_15 represents a rearrangement ‘scar’ that has formed after deletion of these genes. HPT_49 marks a similar rearrangement event (Indel_12). Four genes (PPA0295: Sugar transporter, PPA0296: 4-hydroxythreonine-4-phosphate dehydrogenase, PPA0298: Hypothetical protein and PPA0299: DeoR family transcriptional regulator) have been deleted in clade IA except for the SLST-D strains, leaving only a fragment of the sugar transporter (see Sybil-HPT 49). HPT_52 marks another rearrangement ‘scar’. The sequence of the HPT is not found in clades IB and IC. The other clades have four intact genes (Indel_51) encoding an ABC transport system (Sybil-HPT 52).

## Discussion

Comprehensive genome-based analyses are required to improve our understanding of the complex relationships of *P. acnes* with its human host. Previously a genome comparison between a few strains of *P. acnes* was conducted by Brzuszkiewicz and co-workers^21^. More recently Tomida *et al.* computed the pan-genome of the species^25^. The “blind” script approach of genome comparison is error-prone as genomes are sequenced at variable quality, and annotations are faulty and ambiguous. To accommodate these difficulties, we have established a public Sybil database^26^ for *P. acnes*. The *P. acnes* Sybil database allows users to visually inspect alignments at gene and sequence level of 88 *P. acnes* strains representing the known diversity of the species, in addition to two “*P. humerusii*” strains, and to critically consider our conclusions.

The manual inspections to detect and further investigate indels allowed us to confidently handle cases of ambiguous annotations. With an automatic analysis we would not have been able to reveal that the diversity of gene content for the species is distributed at only 66 loci. The Sybil database is constructed from a reference-free whole genome multiple alignment of the included genomes and allows focusing on a particular clade or a subset of strains. We have not addressed the absence or presence of a described linear plasmid in *P. acnes*^24^, as this plasmid apparently is unstable and easily lost during isolation or preparation for genome sequencing. This plasmid has been found in several strains and was previously described in detail^22^,^24^,^36^.

A general method that will allow genetic modification of any strain of *P. acnes* has not yet been developed. However, using our results, it is possible to correlate phenotypic difference between lineages to genetic candidate loci represented by the indels (supplementary table S1).

Investigating the indels of *P. acnes* resulted in the detection of two areas that might be due to assembly errors. The first area is the duplication of the alanine dehydrogenase encoding gene (PPA2268 and PPA2274) spaced by nine genes. The second example is the large inversion between the three loci of rRNA operons. The inversion in strain HL096PA1 was verified by PCR by Kasimatis *et al.*^24^. However, the reported genome of strain ATCC_11828 has a different inversion between the rRNA operons, which has not been verified yet. It remains to be seen if a large inversion happened twice in clade II genomes while it apparently never happened in other clades or if this is a result of assembly errors in the strains J139 and ATCC_11828. A PCR-based investigation should be performed for both areas during assembly of new genomes of *P. acnes*.

It has been reported that the pan-genome of *P. acnes* is open^25^. However, our data demonstrate that no genes are unique to any single strain, which contradicts this conclusion. The limited number of variable genes is strictly associated with the phylogenetic clades. This suggests that insertions and deletions may be main drivers of diversification of the species into specific clades. It is conceivable that any newly sequenced genomes will not add new genes to the pan-genome, unless it belongs to a new yet unknown phylogenetic lineage. We therefore conclude that the pan-genome of *P. acnes* is closed. Notably, the clade II strains behave as a single clade in terms of shared indels. Thus, an indel found in one clade II strain is found in all clade II strains. Interestingly, the deeper branches in clade II (Fig. 3) thus indicative of more accumulated mutations suggests that clade II is significantly older than any of the clade I sub-clades. The relative stability with regard to gene content of clade II genomes over a long period may be a consequence of the intact CRISPR/cas system in this clade.

Our reference-free approach to identify variable-length HPTs and the subsequent manual evaluation allowed us to distinguish HPTs that likely arose from sequencing errors and authentic HPTs that infer another level of species diversification associated with biological impact.

The use of genome sequences of different geographical and temporal origin, laboratory of processing, and sequencing technologies combined with the inclusion of six high-quality closed representative genomes allow us to rule out biases in the repeat patterns from the processing pipelines. Had we seen a random distribution of the repeat length, it would have been impossible to distinguish random sequence/assembly errors from actual differences in repeat length. Based on the observed clear pattern of repeat length consistent with the population genetic structure of the species, it is conceivable that the noise of errors is limited and not affecting our conclusions.

Our main findings are that HPTs are almost exclusively G or C repeats and that the repeat length variation correlates with the phylogenetic clades. The first finding could be related to the high G+C-content of the genome (60%). However, a binomial likelihood test shows the high G+C content is not the main driver for the identified HPT sequence (>0.0001). The correlation with clades suggests that events of changes are sufficiently rare not to cause significant differences since the divergence of the clades.

The transcriptome analysis revealed how HPTs can impact the expression of genes, and thereby potentially affect phenotypes, primarily between clades. Although *P. acnes* is a highly clonal species, small changes and even SNPs may have an impact on the phenotype. In the absence of effective means of adapting to new challenges, HPTs may serve as a simple method for inactivation of genes or even adjustment of expression levels. Similarly, the exceptional strain C1 may have allowed the transposase element of the arsenic resistance island to be copied within the genome as a high-risk/high-gain method to facilitate adaptation. Thus, three genes effectively were inactivated by the insertion of transposase elements, a sensor histidine kinase MtrB, a NAD-dependent glycerol-3-phosphate dehydrogenase and a glycosyl hydrolase 31 family protein.

We identified two genomic events suggesting a selective pressure towards convergent evolution in *P. acnes*. The acquisition of a hyaluronate-lyase-containing island from two different sources in the clades IB/II and IA/IC and the surprising fact that it was inserted the same place in the genomes is strongly suggesting a mutual selective pressure for this phenotype. Interestingly, the gene is not found in clade III strains, suggesting a different niche or a lower fitness. In the same manner we identified five independent inactivations of the same restriction-modification system. Thus, it is conceivable that *P. acnes* is converging towards better fitness in a fight for nutrients enforced by an assumable easy transmission from face to face of the human host.

## Materials and Methods

### Genome sequences

Six strains of *P. acnes* (PA_12_1_L1, PA_12_1_R1, PA_15_1_R1, PA_15_2_L1, PA_21_1_L1 PA_30_2_L1)^10^ representing different phylogenetic clades of the *P. acnes* population were genome sequenced by shot-gun sequencing using pyrosequencing (454) technology. Assembly resulted in from 4 to 13 contigs. Using the KPA171202 genome (NC_006085.1) as reference the respective genomes were closed by Sanger sequencing of PCR amplicons covering the gaps. Following alignment of corresponding fragments of the six genomes and the KPA171202 genome in MEGA4^37^ every site showing single nucleotide repeats of different lengths were validated or corrected by Sanger sequencing of amplicons of sequences that include the site generated with primer pairs designed on the basis of conserved flanking sequences in Primer-BLAST^38^.

Other genome sequences of *P. acnes* were downloaded from the NCBI Genome and WGS databases. A total of 90 genomes (including six sequenced from this study) were used. Two strains (HL037PA2 and HL037PA3) are likely to belong to the species tentatively named “*Propionibacterium humerusii”*, closely related to *P. acnes*^39^. For the full list of genomes used and accession numbers see supplementary table S4.

### Detection of homopolymeric tracts

Each genome was analysed by a Python script trawling through the sequences to identify locations of single nucleotide repeats longer than six bases. Sequences of 120nt centred on the located HPTs were extracted and stored in a multi-fasta file. The resulting file was processed to remove redundancy, using blastn^40^. The remaining unique HPT candidates were blasted against all included strains, and best hit from each candidate were aligned using muscle^41^ for manual evaluation. Candidates affected by sequencing error or poor sequencing quality at the end of contigs were eliminated according to manual inspection. The manual evaluation reduced the number of HPTs from 86 to 54. For an overview of the sequence difference between strains across the 54 HPT sites see supplementary table S7.

### Sybil database

The strains sequenced for this study where annotated in the CloVR^42^ annotation pipeline. The NCBI Genbank annotation was used for the remaining strains. The storage and processing power was provided by the DIAG cloud computing facility (http://diagcomputing.org/).

A comparative multiple alignment of all 90 genome sequences and generation of a Sybil database were conducted in a CloVR virtual machine image (“clovr-standard-2014-10-07-21-11-54_conv.img”) on the National Science Foundation funded MRI-R2 project #DBI-0959894 facility.

The resulting *P. acnes* Sybil database can be accessed and utilised at http://sybil-clovr.igs.umaryland.edu/sybil/ChristianScholz_Pacnes_sybil. For a video guide on how to use the new database see https://www.youtube.com/user/SybilScreencasts.

### Visualizing data

The distribution of HPTs and indel loci was visualized using BRIG^43^. BRIG was also used to investigate the GC skew of the large inversion in clade II strains. The degree of synteny over the aligned genomes was visualized by the “Synteny Gradient display” option in the Sybil database running on CloVR^26^,^42^.

### RNA preparation, sequencing and sequence analysis

Total RNA from *P. acnes* strains KPA171202 and PA_12_1_L1 grown under anaerobic conditions to the exponential and stationary growth phase in BHI medium at 37 °C was prepared with the RNA PowerSoil^®^ Total RNA Isolation Kit (Mo-Bio, USA), following the instructions described by the manufacturer. RNA quality was controlled using the Bioanalyzer (Agilent Technologies, USA).

The cDNA libraries were constructed by Vertis Biotechnology AG, Germany, as previously described^44^,^45^. The cDNA libraries were sequenced using a HiSeq 2000 machine (Illumina) in single-read mode and 100 cycles. Detailed descriptions of procedures used for quality control, read mapping, expression graph construction and normalization of expression graphs have been published previously^45^. For graph visualization the Integrative Genome Browser (IGB version 8.1.14) was used^46^.

## Additional Information

**Accession codes**: The six genomes are available at the *P. acnes* Sybil database and at GenBank by accession numbers CP012350, CP012351, CP012352, CP012353, CP012354, CP012355, and CP012356.

**How to cite this article**: Scholz, C. F. P. *et al.* Genome stability of *Propionibacterium acnes*: a comprehensive study of indels and homopolymeric tracts.. *Sci. Rep.* **6**, 20662; doi: 10.1038/srep20662 (2016).

## Supplementary Material

Supplementary Information
