# Supplementary material for: Genome stability of Propionibacterium acnes: a comprehensive study of indels and homopolymeric tracts
Source: Sci Rep. 2016 Feb 9;6:20662. doi: 10.1038/srep20662 (PMC4746626; doi:10.1038/srep20662)

Supplementary information

for

Genome stability of *Propionibacterium acnes*: a comprehensive study of indels and homopolymeric tracts.

By

Christian F. P. Scholz, Holger Brüggemann, Hans B. Lomholt, Hervé Tettelin, Mogens Kilian.

Supplementary figure S1

Summary of the predicted evidence of interaction between genes related to iron acquisition as provided by the StringDB v10 analysis.

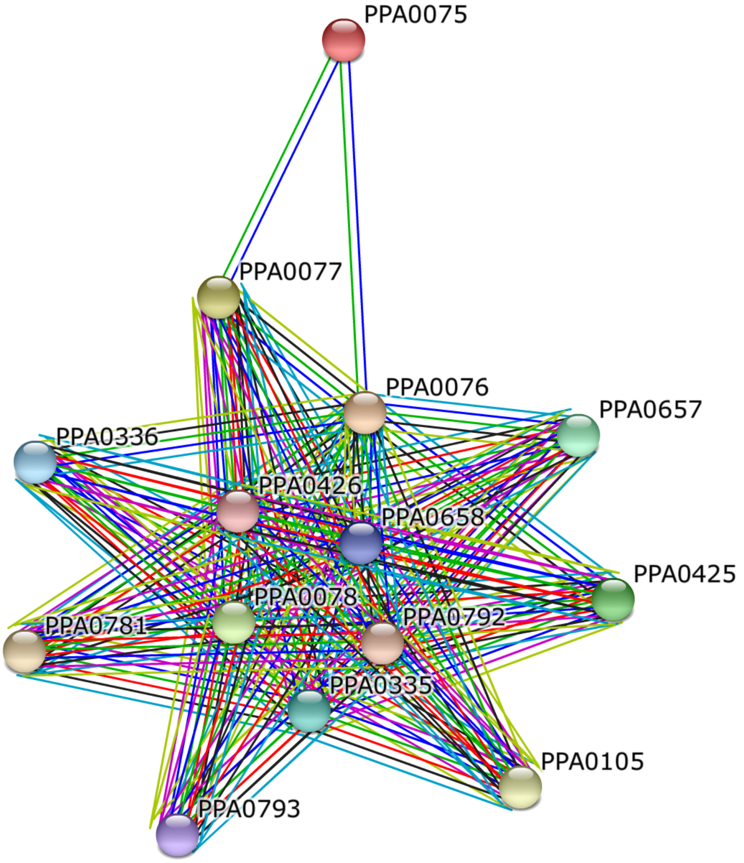

Supplementary figure S2

An alignment of two clade III strains (HL201PA1 and PMH5), a clade IB strain (KPA171202), a clade IA strain (266) and a ”*P. humerusii*” strain (HL037PA2).

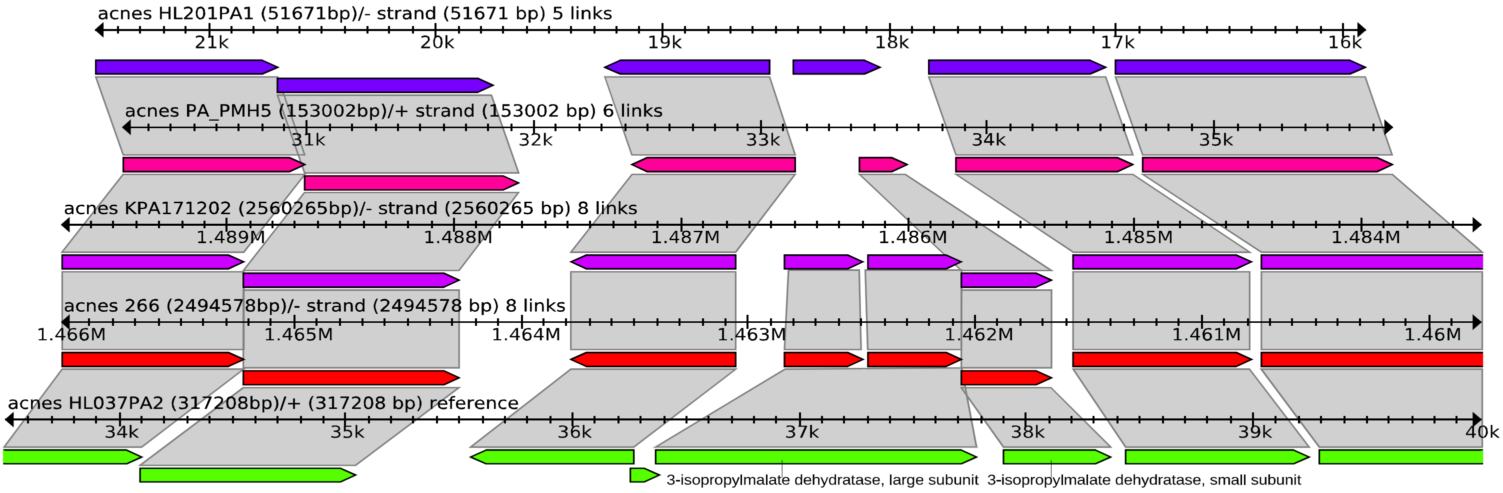

Supplementay table S1. A list of indels indicating absence/presence with reference to SLST clades. The table includes a direct link to the Sybil database for each indel, as well as a short title of predicted function and coordinates according to the KPA171202 genome.

		Carbohydrate processing	Iron transport	Ricin B lectin related	Resistance to arsenic an[illegible] cadmium	Resolvase related	Hypothetical genes	Streptolysin		Putative DNA binding
Location	7000	23000	82000	102500	103600	146200	146200	185000	206000	220000
# of genes	-	6-7	4	3-4	32	7	3-4	27	-	2
InDel #	InDel01	InDel02	InDel03	InDel04	InDel05	InDel06a	InDel06b	InDel07	InDel08	InDel09
**Type I**	Complex	-	Partly	-	Partly	Partly	Partly	Partly	Complex	-
**Type IA1**	Complex	-	Present	-	Partly	Partly	-	Partly	Complex	-
SLST-A	Complex	-	Present	-	Partly	Partly	-	-	Complex	-
SLST-B	Complex	-	Present	-	-	-	-	-	Complex	-
SLST-C	Complex	-	Present	-	-	Present	-	Present	Complex	-
SLST-D	Complex	-	Present	-	-	-	-	-	Complex	-
SLST-E	Complex	-	Present	-	-	-	-	-	Complex	-
**Type IA2**	Complex	-	Present	-	-	-	-	-	Complex	-
**Type IB**	Complex	-	Present	-	-	-	-	-	Complex	-
**Type IC**	Complex	-	-	-	-	-	Present	-	Complex	-
**Type II**	Complex	Present	-	Present	-	-	Present	-	Complex	Present
**Type III**	Complex	-	Present	Present	-	-	-	-	Complex	Present

	YD repeat protein and type IV secretion	Type II secretion	Sugar transporter	ABC transporter	Hyaloronate lyase	3-ketoacyl-ACP reductase	Two-component system	Divergent AAA protein	ABC transporter	Inositol transport/modificat[illegible]
Location	240000	323500	347000	382000	425000	425000	459000	472000	472000	500000
# of genes	?	10-11	3	2-3	3	6-7	2	1-2	3	7
InDel #	InDel10	InDel11	InDel12	InDel13	InDel14a	InDel14b	InDel15	InDel16a	InDel16b	InDel17
**Type I**	Partly	-	Partly	-	Present	Partly	Partly	Partly	-	-
**Type IA1**	Present	-	Partly	-	Present	-	Partly	Partly	-	-
SLST-A	Present	-	-	-	Present	-	Present	-	-	-
SLST-B	Present	-	-	-	Present	-	Present	-	-	-
SLST-C	Present	-	-	-	Present	-	Present	-	-	-
SLST-D	Present	-	Present	-	Present	-	Present	-	-	-
SLST-E	Present	-	-	-	Present	-	-	Present	-	-
**Type IA2**	Present	-	-	-	Present	-	Present	-	-	-
**Type IB**	Present	-	Present	-	Present	Present	Present	-	-	-
**Type IC**	-	-	Present	-	Present	-	Present	-	-	-
**Type II**	Present	-	Present	Present	Present	Present	Present	-	-	-
**Type III**	Present	Present	Present	-	-	-	Present	-	Present	Present

	Tyrosine transport/modification		Alcohol dehydrogenase	Fucose transport/modification	Type I restriction enzyme	Hemin ABC transporter, fecCD transport	Gluconate:proton sym-porter, Shikimate kinase	Magnesium-chelatase, iron-transport, bacteriocin		Hypothetical
Location	548500	570000	590000	670000	719000	746000	810000	870000	940000	1056000
# of genes	7-8	3	3	9-10	7	5-6	2	10-11	-	1-2
InDel #	InDel18	InDel19	InDel20	InDel21	InDel22	InDel23	InDel24	InDel25	InDel26	InDel27
**Type I**	Partly	Present	Present	Partly	-	-	Patly	Present	Complex	Present
**Type IA1**	Partly	Present	Present	Partly	-	-	-	Present	Complex	Present
SLST-A	-	Present	Present	Present	-	-	-	Present	Complex	Present
SLST-B	-	Present	Present	Present	-	-	-	Present	Complex	Present
SLST-C	-	Present	Present	Present	-	-	-	Present	Complex	Present
SLST-D	Present	Present	Present	Present	-	-	-	Present	Complex	Present
SLST-E	-	Present	Present	Present	-	-	-	Present	Complex	Present
**Type IA2**	-	Present	Present	Partly	-	-	-	Present	Complex	Present
**Type IB**	-	Present	Present	Present	-	-	Present	Present	Complex	Present
**Type IC**	-	Present	Present	-	-	Present	-	Present	Complex	Present
**Type II**	-	Present	-	-	Present	Present	Present	Present	Complex	Present
**Type III**	-	-	Present	-	-	Present	Present	-	Complex	-

	Beta-glucosidase	?	Gyrase, integrase, transposase	Tryptophan synthase	Transposase, phage related	Phage integrase	Transposase	?	PadR, BcrA	3-isopropylmalate dehydrogenase	Integrase
Location	1077000	1131000	1167500	1229000	1274000	1319200	1340000	1400000	1467500	1486000	1545000
# of genes	1	2	10	½	20-26	4	2	?	6	2	3
InDel #	InDel28	InDel29	InDel30	InDel31	InDel32	InDel33	InDel34	InDel35	InDel36	InDel37	InDel38
**Type I**	-	Present	-	-	-	Partly	Partly	Complex	-	Present	-
**Type IA1**	-	Present	-	-	-	Partly	Partly	Complex	-	Present	-
SLST-A	-	Present	-	-	-	-	Partly	Complex	-	Present	-
SLST-B	-	Present	-	-	-	-	-	Complex	-	Present	-
SLST-C	-	Present	-	-	-	-	-	Complex	-	Present	-
SLST-D	-	Present	-	-	-	-	-	Complex	-	Present	-
SLST-E	-	Present	-	-	-	-	-	Complex	-	Present	-
**Type IA2**	-	Present	-	-	-	Partly	-	Complex	-	Present	-
**Type IB**	-	Present	-	-	-	-	-	Complex	-	Present	-
**Type IC**	-	Present	-	-	-	-	-	Complex	-	Present	-
**Type II**	Present	Present	-	Present	-	-	-	Complex	Present	Present	-
**Type III**	-	-	Present	-	Present	-	-	Complex	-	-	Present

	Dipeptide transport system	Actinobacterial surface protein	Hypothetical	Maltose transport, TraL	Transposase	Cryptic phage	Restriction enzyme	Hypothetical	DNA polymerase III	Von Willebrand factor d- main
Location	1579500	1623200	1631000	1670000	1688000	1725000	1725000	1779500	1800000	1822000
# of genes	7	2	3	3	2	27	5-6	2	15	15
InDel #	InDel39	InDel40	InDel41	InDel42	InDel43	InDel44a	InDel44b	InDel45	InDel46	InDel47
**Type I**	Present	Present	-	Present	Partly	Partly	Present	-	Present	Partly
**Type IA1**	Present	Present	-	Present	Partly	-	Present	-	Present	Partly
SLST-A	Present	Present	-	Present	Partly	-	Present	-	Present	-
SLST-B	Present	Present	-	Present	-	-	Present	-	Present	-
SLST-C	Present	Present	-	Present	-	-	Present	-	Present	-
SLST-D	Present	Present	-	Present	-	-	Present	-	Present	Partly
SLST-E	Present	Present	-	Present	-	-	Present	-	Present	-
**Type IA2**	Present	Present	-	Present	-	-	Present	-	Present	-
**Type IB**	-	Present	-	Present	-	Present	Partly	-	Present	-
**Type IC**	Present	Present	-	Present	-	-	Present	-	Present	-
**Type II**	-	Partly	Present	Present	-	-	-	Partly	Present	-
**Type III**	-	Present	-	-	-	-	Present	-	Present	Present

	Beta-N-acetylhexosamini dase	Nickel import	-	Membrane proteins	Transport system	Trombospondin type 3 re peat protein	Oligopeptide transport	Transport, Transcription regulators and sugar pro- cessing	Transposase	Proccesing of sugar/amino acid
Location	1990000	2007000	2070000	2106000	2127000	2155000	2172500	2240000	2280000	2307000
# of genes	1-2	3-4	-	3-4	2		4	15	2	7
InDel #	InDel48	InDel49	InDel50	InDel51	InDel52	InDel53	InDel54	InDel55	InDel56	InDel57
**Type I**	Partly	-	Complex	Present	Present	-	-	Partly	Partly	-
**Type IA1**	Partly	-	Complex	Present	Present	-	-	-	Partly	-
SLST-A	-	-	Complex	Present	Present	-	-	-	Partly	-
SLST-B	-	-	Complex	Present	Present	-	-	-	-	-
SLST-C	-	-	Complex	Present	Present	-	-	-	-	-
SLST-D	-	-	Complex	Present	Present	-	-	-	-	-
SLST-E	Present	-	Complex	Present	Present	-	-	-	-	-
**Type IA2**	Present	-	Complex	Present	Present	-	-	-	-	-
**Type IB**	Present	-	Complex	-	Present	-	-	Present	-	-
**Type IC**	Present	-	Complex	-	Present	-	-	-	-	-
**Type II**	Present	Present	Complex	Present	-	-	-	-	-	-
**Type III**	Present	Present	Complex	-	Present	-	Present	Present	-	Present

	CRISPR/CAS locus	Iso-amylase domain	Thiazylpeptide bacteriocin	dihydrodipicolinate syn- thase	Amino acid proccessing	,	Sugar transport	Carbohydrate modification	Transposase	Periplasmic binding pr[illegible] tein
Location	2354000	2367700	2390000	2392500	2460000	2495000	2512000	2527000	2535000	128000
# of genes	10-14	1	3	1	9	21	2	5	2	1
InDel #	InDel58	InDel59	InDel60a	InDel60b	InDel61	InDel62	InDel63	InDel64	InDel65	InDel66
**Type I**	-	-	Partly	Partly	Partly	Partly	Partly	-	Partly	Present
**Type IA1**	-	-	Partly	Partly	Present	Partly	Present	-	Partly	Present
SLST-A	-	-	Present	-	Present	-	Present	-	Partly	Present
SLST-B	-	-	Present	-	Present	-	Present	-	-	Present
SLST-C	-	-	Present	-	Present	-	Present	-	-	Present
SLST-D	-	-	-	Present	Present	-	Present	-	-	Present
SLST-E	-	-	Present	-	Present	Present	Present	-	-	Present
**Type IA2**	-	-	Present	-	Present	Partly	Present	-	-	Present
**Type IB**	-	-	Present	-	Present	-	Present	-	-	Present
**Type IC**	-	-	Present	-	Partly	Present	-	-	-	Present
**Type II**	Present	Present	-	-	Present	-	Present	Present	-	Present
**Type III**	Rudiments	-	-	Present	Present	-	Present	-	-	-

Supplementary table S2.

Variation in repeat-length across SLST clades for each HPT, described by missing nucleotides relative to the longest repeat sequence

	SLST phylotypes									
	A n=40	B n=2	C n=18	D n=4	E n=7	F n=20	G n=2	H n=8	K n=13	L n=3
HPT 01	3	3	3	3	3	3-6	3	3	0-3	3
HPT 02	0-5	1-3	0-4	5	5	0-4	5	5	6	5
HPT 03	0-4	3	3-4	3	3	3	3	3	3-4	3
HPT 04	1-3	2	0-2	1-2	2-3	1-2	1	1-2	2	2
HPT 05	0-5	4	4	4	4	4	4	4	-	4
HPT 06	-	-	-	-	-	-	-	2-5	0-7	-
HPT 07	0-5	5	6-7	6	5-6	5-6	6	6	6	6-7
HPT 08	2	2	2	2	2	1-3	2	2	2	1
HPT 09	8	8	8	8	8	8	8	8	0-8	8
HPT 10	6-7	6	6-7	6	1-6	6-7	6	6	1-6	5-6
HPT 11	1-11	11	6-11	12	11-12	11	12	12	11	11
HPT 12	1-3	2	1-2	2	2	2	0	2	2	-
HPT 13	1	1	1	1	0-1	1	1	1	1	-
HPT 14	5	5	2-5	6	0-4	5	5	4	-	4
HPT 15	0-3	3	3	4	4	4	3	4	-	-
HPT 16	2-6	5	5	5	5	3-5	5	6	7	-
HPT 17	0-4	1-3	1-3	4	1-4	3-4	4	4	4	4
HPT 18	4-5	5	5	4	3-4	1-4	5	5	2-4	6
HPT 19	3-4	4	4	4	3-4	3-5	4	3-5	1-4	4
HPT 20	2	2	2	0-1	0	2	2	0	0	1
HPT 21	3-10	9	7-9	10	9	8-10	10	9	11	-
HPT 22	0-6	6	6	3-5	6	6	4	6	6	7
HPT 23	2-3	2	2	2	2	2	0	2	1-3	3
HPT 24	1-4	4	4	4	0-4	4	-	4-5	-	-
HPT 25	2-3	2	2	2	2-3	2-3	0	1-2	-	1-2
HPT 26	19-20	20	18-20	19	19-20	20	20	2-20	2	-
HPT 27	5	5	5	5	0-3	0-4	4	4	1-4	5
HPT 28	6	5-6	0-5	6	6	6	6	6	6	6
HPT 29	4-10	6	1-8	1-2	1-2	3	-	6-8	8-9	4
HPT 30	13-15	14	14	14	14	14	8	2	2	0-1
HPT 31	4	4	4	4	4	0-4	4	4	4	4
HPT 32	0-8	9	5-8	6-8	8	8	8	6-7	8	8
HPT 33	2-9	3-7	4-11	6-7	5-7	3-9	-	6-9	-	10
HPT 34	0-6	6	6	6	6	6	6	6	6	6
HPT 35	0-1	0	0-1	1	1	1-2	0	0-1	0	-
HPT 36	1-6	2-6	0-3	6-7	2-6	5-6	6	2-3	6	2-6
HPT 37	0-3	2-3	3	3	3	3	3	4	4	4
HPT 38	4-5	5	5	5-6	3-5	0-5	4	5	6	2-5
HPT 39	6-7	7	7	7	7	7	7	0-6	7	-
HPT 40	2	2	2	2	2	2	2	2	0-2	2
HPT 41	3	3	1-3	3	3	2-3	3	3	3	3
HPT 42	2-5	3	3-4	3-4	2-5	5	4	2-3	1-5	4
HPT 43	2	2	2	1	2	2	2	2	0-2	2
HPT 44	4	-	4	4	4	4	-	0-4	4	4
HPT 45	6-7	7	1-5	7	7	7	-	7	3-5	6
HPT 46	1-4	3-4	4	4	3-4	0-3	-	4	5	5
HPT 47	7	7	7	7	7	7	7	0-4	0-4	4
HPT 48	0-5	0-2	0-4	3-4	1-4	0-4	0	5	-	11
HPT 49	1-4	1-2	1-3	2	2-3	0-3	3	3	3	3
HPT 50										
HPT 51	12	12	1-12	11-12	12	12	12	12	12	12
HPT 52	2-7	7	0-6	5-7	7	3-8	-	-	0-7	-
HPT 53	-	-	-	-	-	-	-	-	0-3	6
HPT 54	4-5	5	4-5	5	5	1-4	5	-	7	6

Supplementary table S3. List of HPTs including links to the Sybil database, location according to the KPA171202 genome, nearby annotation and type of HPT.

HPT #	Nucleotide type	Gene annotation NCBI	Link to Sybil	Gene relation	KPA171202 coordinates
HPT 01	G/C	Acetyltransferase, GNAT family	Link	intergenic	35554:35593
HPT 02	G/C	Hypothetical protein	Link	intergenic	1025365:1025388
HPT 03	G/C	Glycosyl hydrolase	Link	Intragenic	101518:101544
HPT 04	G/C	GntR family transcriptional regulator	Link	intergenic	199410:199437
HPT 05	G/C	RHS-family protein / YD repeat protein (3 repeats)	Link	intergenic	249739:249764
HPT 06	G/C	Putative oxidoreductase	Link	Intragenic	424850:424877
HPT 07	G/C	Hypothetical protein	Link	? / Intragenic	494973:494999
HPT 08	G/C	Molybdenum cofactor synthesis domain protein	Link	intergenic	565070:565097
HPT 09	G/C (GA repeat)	Sodium neurotransmitter symporter family protein	Link	Intragenic	616529:616545
HPT 10	G/C	Hydrolase, alpha/beta domain protein	Link	Intragenic	808256:808279
HPT 11	G/C (A/T nearby)	HtaA domain protein, Putative Fe-transport	Link	intergenic	856169:856202
HPT 12	G/C	CobN/magnesium chelatase subunit-	Link	Intragenic	873556:873582
HPT 13	A/T	CobN/magnesium chelatase subunit-	Link	Intragenic	874120:874148
HPT 14	G/C	GntR family transcriptional regulator	Link	? / Intragenic	950951:950975
HPT 15	G/C	putative beta-glycosidase	Link	? / Intragenic	1076736:1076761
HPT 16	G/C	Acetyltransferase, GNAT family	Link	intergenic	1131631:1131663
HPT 17	G/C	Molecular chaperon /BnaK domain protein	Link	Intragenic	1189744:1189768
HPT 18	G/C	Putative sensor histidine kinase PdtaS	Link	intergenic	1372989:1373014
HPT 19	G/C	Hypothetical protein	Link	Intragenic	1406560:1406593
HPT 20	G/C	Preprotein translocase SecA subunit	Link	intergenic	1455191:1455211
HPT 21	G/C	3-isopropylmalate dehydratase large/small subunit	Link	intergenic	1485771:1485819
HPT 22	G/C (A/T)	50S ribosomal protein L20	Link	intergenic	1535003:1535026
HPT 23	G/C	translation initiation factor IF-2	Link	intergenic	1612804:1612831
HPT 24	G/C	Hypothetical protein	Link	intergenic	1657456:1657481
HPT 25	G/C	D-serine/D-alanine/glycine transporter	Link	intergenic	1789777:1789810
HPT 26	G/C (In/del nearby)	Glycosyl hydrolase family 25 / lysozyme M1	Link	intergenic	1815548:1815584
HPT 27	G/C	Putative magnesium and cobolt transport CorA	Link	intergenic	1869600:1869634
HPT 28	G/C	Cardiolipin synthetase or phospholipase D	Link	Intragenic	1924263:1924286
HPT 29	G/C (GA repeat)	Hypothetical protein	Link	intergenic	1935888:1935911
HPT 30	G/C (In/del)	Putative holo-[acyl-carrier-protein] synthase	Link	intergenic	1958937:1958964
HPT 31	G/C	endo-beta-N-acetylglucosaminidase family protein	Link	intergenic	1967962:1967986
HPT 32	G/C	Hypothetical protein	Link	Intragenic	2032637:2032658
HPT 33	G/C	Adhesion or S-layer protein	Link	intergenic	2039174:2039199
HPT 34	G/C	50S ribosomal protein L10	Link	intergenic	2050286:2050309
HPT 35	A/T	Protein associated to adhesion protein	Link	? / Intragenic	2069318:2069346
HPT 36	G/C	ABC Transporter, ATP-binding protein	Link	Intragenic	2074490:2074507
HPT 37	G/C	ubiquinone/menaquinone methyltransferase UbiE	Link	intergenic	2103894:2103925
HPT 38	G/C (A/T nearby)	Peptidoglycan binding domain	Link	intergenic	2177057:2177094
HPT 39	G/C	Adhesion or S-layer protein	Link	Intragenic	2302361:2302390
HPT 40	G/C	Class I glutamine aminotransferase	Link	Intragenic	2370041:2370068
HPT 41	G/C	Long-chain fatty-acid—CoA ligase/synthetase	Link	intergenic	2419073:2419099
HPT 42	G/C	3-methyladenine DNA glycosylase	Link	End-End	2430915:2430943
HPT 43	G/C	Glycerol-3-phosphate dehydrogenase	Link	intergenic	2435469:2435495
HPT 44	G/C	Glycerate kinase	Link	End-End	2494978:2495000
HPT 45	G/C (A/T)	Thioredoxin disulfide reductase	Link	End-End	2509661:2509688
HPT 46	G/C	Thioredoxin disulfide reductase	Link	intergenic	2508371:2508396
HPT 47	G/C	Hypothetical protein	Link	intergenic	1249158:1249187
HPT 48	G/C	Hypothetical protein	Link	intergenic	31654:31684
HPT 49	G/C	Inkonsistent annotation	Link	Intragenic	345730:345753
HPT 50	G/C (A/T)	Downstream of 5Srna	Link	intergenic	Multiple loci
HPT 51	G/C (A/T)	-	Link	? / Intragenic	1385893:1385915
HPT 52	G/C	Hypothetical protein	Link	Intragenic	2059397:2059419 (266)
HPT 53	G/C	No annotation nearby	Link	intergenic	785167:785193(ATCC11828)
HPT 54	G/C	Hypothetical protein	Link	intergenic	461950:461979(266)

Supplementary table S4. Strains used in this study, including information on MLST9 types, SLST types and accession numbers.

#	Strain name	SLST	Phylotypes	MLST9 CC	MLST9	Accesion No.	Status	Country
1	P. acnes 266	A1	IA	CC18	ST18	PRJNA162059	Genome	Germany
2	P. acnes 6609	H1	IB	CC36	ST93	PRJNA162137	Genome	Hungary
3	P. acnes ATCC 11828	K9	II	CC53	ST85	PRJNA162177	Genome	Hungary
4	P. acnes C1	A5	IA	Singleton	ST91	PRJNA176501	Genome	Japan
5	P. acnes DSM 1897	A1	IA	CC18	ST18	PRJNA169817	Contigs	USA
6	P. acnes FZ1/2/0	B1	IA	Singleton	ST92	PRJNA192733	Contigs	Hungary
7	P. acnes HL001PA1	K1	II	CC53	ST60	PRJNA181484	Contigs	USA
8	P. acnes HL002PA1	F1	IA	CC28	ST88	PRJNA181433	Contigs	USA
9	P. acnes HL002PA2	A2	IA	CC18	ST59	PRJNA181481	Contigs	USA
10	P. acnes HL002PA3	A2	IA	CC18	ST59	PRJNA181454	Contigs	USA
11	P. acnes HL005PA1	C2	IA	CC3	ST3	PRJNA181478	Contigs	USA
12	P. acnes HL005PA2	A1	IA	CC18	ST12	PRJNA181476	Contigs	USA
13	P. acnes HL005PA3	A1	IA	CC18	ST12	PRJNA181475	Contigs	USA
14	P. acnes HL005PA4	F1	IA	CC28	ST28	PRJNA181485	Contigs	USA
15	P. acnes HL007PA1	C1	IA	CC3	ST3	PRJNA181446	Contigs	USA
16	P. acnes HL013PA1	F1	IA	CC28	ST87	PRJNA181470	Contigs	USA
17	P. acnes HL013PA2	A1	IA	CC18	ST18	PRJNA181450	Contigs	USA
18	P. acnes HL020PA1	A1	IA	CC18	ST77	PRJNA181451	Contigs	USA
19	P. acnes HL025PA1	D1	IA	CC28	ST27	PRJNA181453	Contigs	USA
20	P. acnes HL025PA2	F1	IA	CC28	ST65	PRJNA181477	Contigs	USA
21	P. acnes HL027PA1	F1	IA	CC28	ST28	PRJNA181434	Contigs	USA
22	P. acnes HL027PA2	A1	IA	CC18	ST12	PRJNA181456	Contigs	USA
23	P. acnes HL030PA1	H1	IB	CC36	ST36	PRJNA181438	Contigs	USA
24	P. acnes HL030PA2	F4	IA	CC28	ST66	PRJNA181436	Contigs	USA
25	P. acnes HL036PA1	A2	IA	CC18	ST8	PRJNA181448	Contigs	USA
26	P. acnes HL036PA2	A2	IA	CC18	ST8	PRJNA181457	Contigs	USA
27	P. acnes HL036PA3	A2	IA	CC18	ST8	PRJNA181435	Contigs	USA
28	P. acnes HL037PA1	F6	IA	CC28	ST67	PRJNA181471	Contigs	USA
29	P. acnes HL038PA1	C1	IA	CC3	ST3	PRJNA181440	Contigs	USA
30	P. acnes HL042PA3	K2	II	CC53	ST73	PRJNA225886	Contigs	USA
31	P. acnes HL043PA1	C1	IA	CC3	ST58	PRJNA181426	Contigs	USA
32	P. acnes HL043PA2	C1	IA	CC3	ST58	PRJNA181449	Contigs	USA
33	P. acnes HL045PA1	C2	IA	CC3	ST3	PRJNA181463	Contigs	USA
34	P. acnes HL046PA1	F1	IA	CC28	ST28	PRJNA181479	Contigs	USA
35	P. acnes HL046PA2	A2	IA	CC18	ST59	PRJNA181458	Contigs	USA
36	P. acnes HL050PA1	F1	IA	CC28	ST28	PRJNA181474	Contigs	USA
37	P. acnes HL050PA2	K4	II	CC53	ST60	PRJNA181439	Contigs	USA
38	P. acnes HL050PA3	F1	IA	CC28	ST28	PRJNA181473	Contigs	USA
39	P. acnes HL053PA1	C2	IA	CC3	ST3	PRJNA181466	Contigs	USA
40	P. acnes HL053PA2	E1	IA	CC31	ST31	PRJNA181464	Contigs	USA
41	P. acnes HL056PA1	C2	IA	CC3	ST3	PRJNA181445	Contigs	USA
42	P. acnes HL059PA1	F1	IA	CC28	ST28	PRJNA181459	Contigs	USA
43	P. acnes HL059PA2	F1	IA	CC28	ST28	PRJNA181429	Contigs	USA
44	P. acnes HL060PA1	K1	II	CC53	ST69	PRJNA181441	Contigs	USA
45	P. acnes HL063PA1	A1	IA	CC18	ST18	PRJNA181447	Contigs	USA
46	P. acnes HL063PA2	F4	IA	CC28	ST70	PRJNA181455	Contigs	USA
47	P. acnes HL067PA1	F2	IA	CC28	ST64	PRJNA181482	Contigs	USA
48	P. acnes HL072PA1	A6	IA	CC18	ST18	PRJNA181468	Contigs	USA
49	P. acnes HL072PA2	A6	IA	CC18	ST18	PRJNA181428	Contigs	USA
50	P. acnes HL074PA1	C2	IA	CC3	ST3	PRJNA181462	Contigs	USA
51	P. acnes HL078PA1	B1	IA	Singleton	ST89	PRJNA181469	Contigs	USA
52	P. acnes HL082PA1	E5	IA	CC31	ST31	PRJNA181472	Contigs	USA
53	P. acnes HL082PA2	K6	II	Singleton	ST62	PRJNA181480	Contigs	USA

#	Strain name	SLST	Phylotypes	MLST9 CC	MLST9	Accesion No.	Status	Country
54	P. acnes HL083PA1	C1	IA	CC3	ST3	PRJNA181465	Contigs	USA
55	P. acnes HL083PA2	F3	IA	CC28	ST28	PRJNA181430	Contigs	USA
56	P. acnes HL086PA1	E4	IA	CC31	ST31	PRJNA181452	Contigs	USA
57	P. acnes HL087PA1	F1	IA	CC28	ST28	PRJNA181427	Contigs	USA
58	P. acnes HL087PA2	A1	IA	CC18	ST18	PRJNA181460	Contigs	USA
59	P. acnes HL087PA3	F1	IA	CC28	ST28	PRJNA181437	Contigs	USA
60	P. acnes HL092PA1	E1	IA	CC31	ST31	PRJNA181431	Contigs	USA
61	P. acnes HL096PA1	C1	IA	CC3	ST3	PRJNA198524	Genome	USA
62	P. acnes HL096PA2	C1	IA	CC3	ST3	PRJNA181416	Contigs	USA
63	P. acnes HL096PA3	A1	IA	CC18	ST18	PRJNA181415	Contigs	USA
64	P. acnes HL097PA1	G1	IC	Singleton	ST74	PRJNA181419	Contigs	USA
65	P. acnes HL099PA1	C1	IA	CC3	ST3	PRJNA181418	Contigs	USA
66	P. acnes HL103PA1	K1	II	CC53	ST60	PRJNA181417	Contigs	USA
67	P. acnes HL110PA1	E1	IA	CC31	ST31	PRJNA181467	Contigs	USA
68	P. acnes HL110PA2	E3	IA	CC31	ST31	PRJNA181461	Contigs	USA
69	P. acnes HL110PA3	K2	II	CC53	ST73	PRJNA181443	Contigs	USA
70	P. acnes HL110PA4	K2	II	CC53	ST73	PRJNA181442	Contigs	USA
71	P. acnes HL201PA1	NT*	III	NT*	NT*	PRJNA224116	Contigs	USA
72	P. acnes HL202PA1	K13	II	NT*	NT*	PRJNA224116	Contigs	USA
73	P. acnes J139	K8	II	CC53	ST73	PRJNA42957	Contigs	USA
74	P. acnes J165	A1	IA	CC18	ST18	PRJNA46203	Contigs	USA
75	P. acnes KPA171202	H2	IB	CC36	ST36	PRJNA58101	Genome	Germany
76	P. acnes P.acn17	F5	IA	CC28	ST70	PRJNA80735	Genome	UK
77	P. acnes P.acn31	F4	IA	CC28	ST67	PRJNA80733	Genome	UK
78	P. acnes P.acn33	F1	IA	Singleton	ST86	PRJNA80745	Genome	UK
79	P. acnes PA_12_1_L1	A1	IA	CC18	ST18	PRJNA292485	Genome	Denmark
80	P. acnes PA_12_1_R1	A1	IA	CC18	ST20	PRJNA292486	Genome	Denmark
81	P. acnes PA_15_1_R1	C1	IA	CC3	ST3	PRJNA291008	Genome	Denmark
82	P. acnes PA_15_2_L1	A1	IA	CC18	ST29	PRJNA292484	Genome	Denmark
83	P. acnes PA_21_1_L1	H1	IB	CC36	ST36	PRJNA292488	Genome	Denmark
84	P. acnes PA_30_2_L1	D1	IA	CC28	ST27	PRJNA292487	Genome	Denmark
85	P. acnes PMH5	L1	III	Singleton	ST44	PRJNA292393	Genome	Denmark
86	P. acnes PMH7	L1	III	Singleton	ST44	PRJNA292394	Genome	Denmark
87	P. acnes PRP-38	G1	IC	Singleton	ST74	PRJNA180249	Contigs	UK
88	P. acnes SK137	C1	IA	CC3	ST3	PRJNA48071	Genome	USA
89	P. acnes SK182	C1	IA	CC3	ST3	PRJNA179860	Contigs	USA
90	P. acnes SK187	E2	IA	CC28	ST67	PRJNA46201	Contigs	USA
91	P. sp. 409-HC1	D1	IA	CC28	ST27	PRJNA67825	Contigs	USA
92	P. sp. 5_U_42AFAA	A1	IA	CC18	ST29	PRJNA75117	Contigs	Canada
93	P. sp. CC003-HC2	D1	IA	CC28	ST27	PRJNA71185	Contigs	USA
94	P. sp. KPL1847	K4	II	CC53	ST60	PRJNA224283	Contigs	USA
95	P. sp. KPL1849	K4	II	CC53	ST60	PRJNA224282	Contigs	USA
96	P. sp. KPL1854	C3	IA	CC3	ST3	PRJNA224280	Contigs	USA
97	P. sp. KPL2003	A1	IA	CC18	ST18	PRJNA224278	Contigs	USA
98	P. sp. KPL2008	H1	IB	CC36	ST36	PRJNA224276	Contigs	USA
99	P. sp. KPL2009	C1	IA	CC3	ST3	PRJNA224275	Contigs	USA

* Not typed, needs verification by Sanger sequencing.

HPT#: HPT01
Gene: Acetyltransferase, GNAT family
PPA: PPA0037
Location: 35554:35593 (KPA171202)
Page: 1/54

Supplementary figure S3.

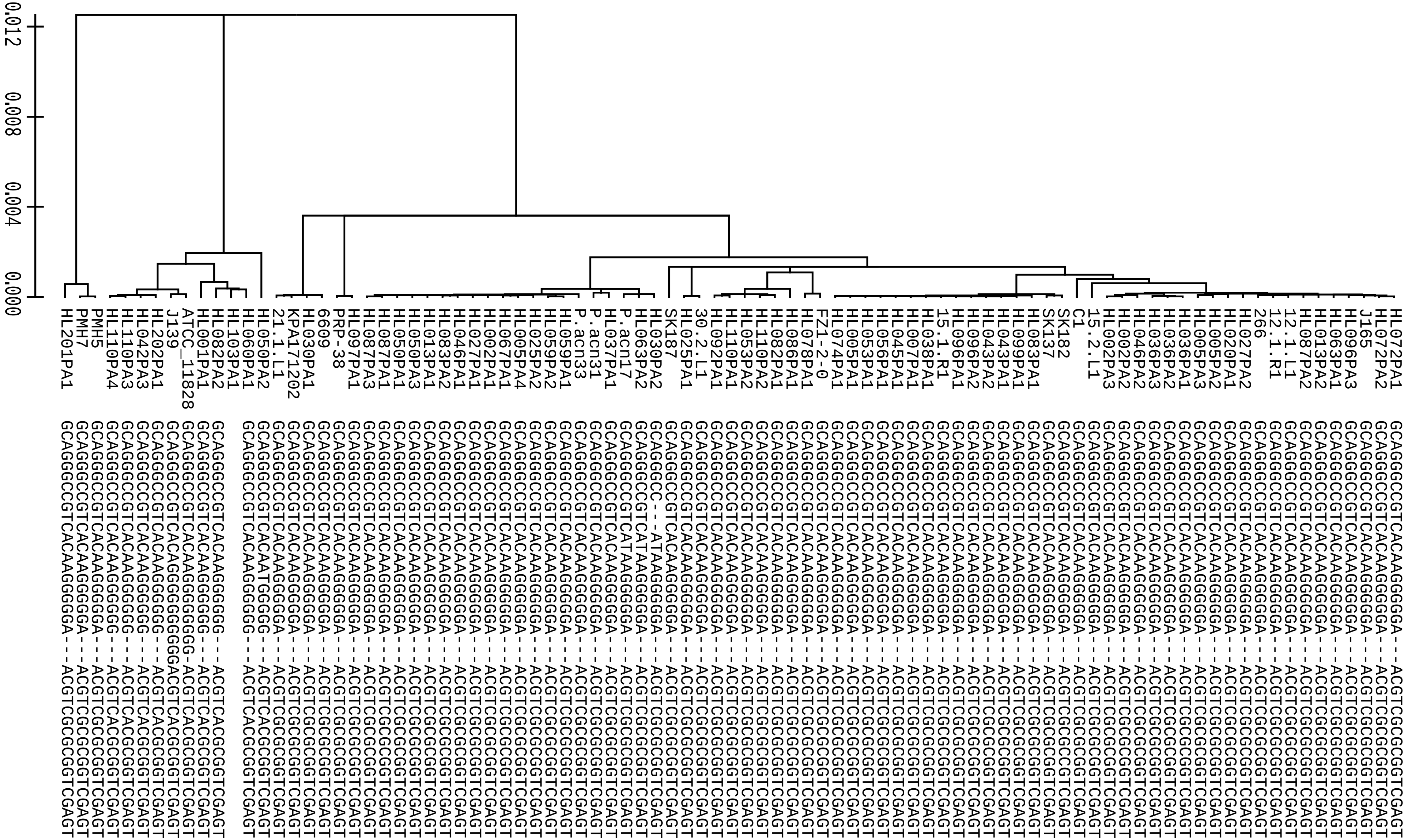

HPT#: HPT02
Gene: Hypothetical Protein
PPA: PPA0089
Location: 1025365:1025388(KPA171202)
Page: 2/54

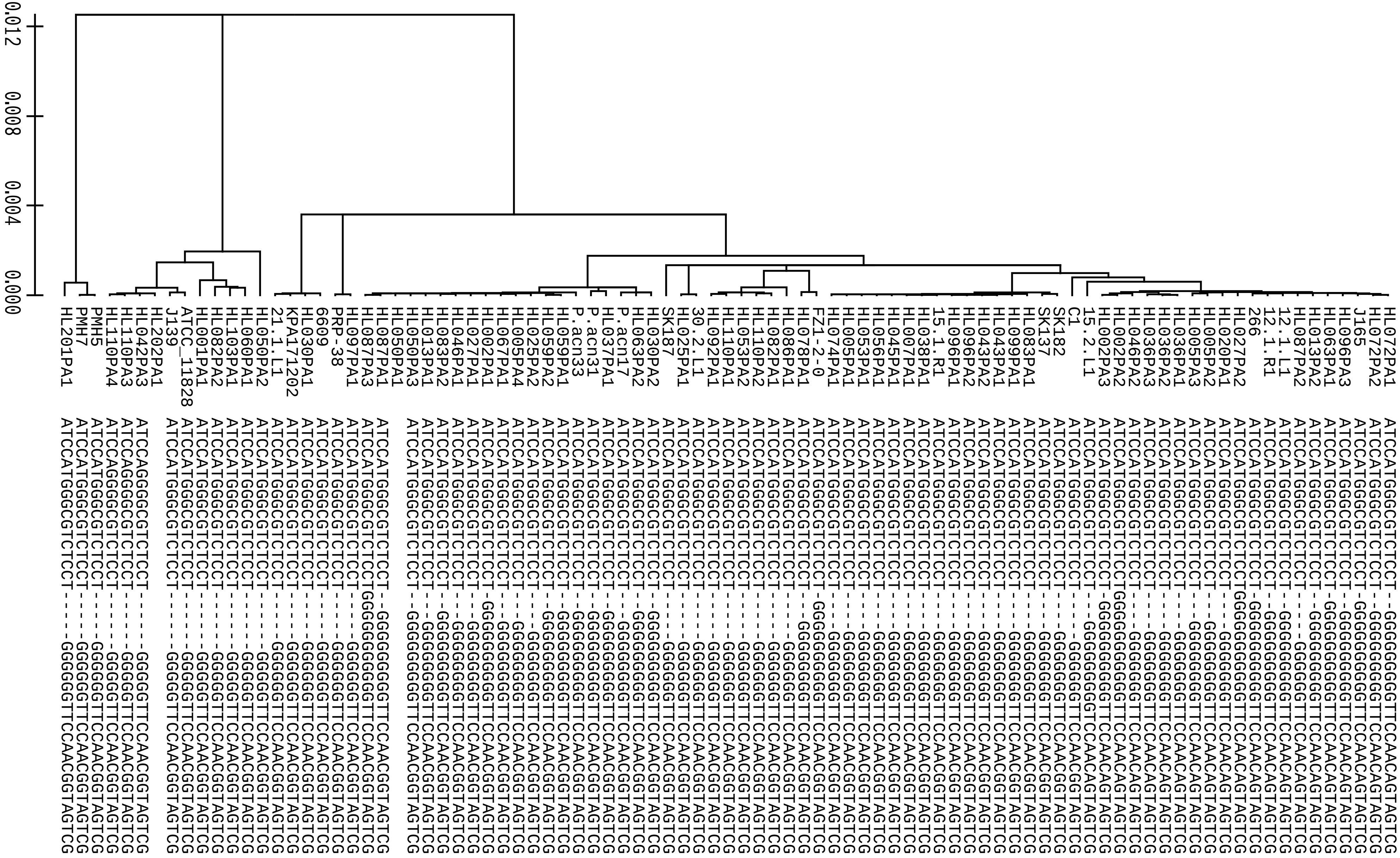

HPT#: HPT03
Gene: Glycosyl hydrolase
PPA: PPA0089
Location: 101518:101544 (KPA171202)
Page: 3/54

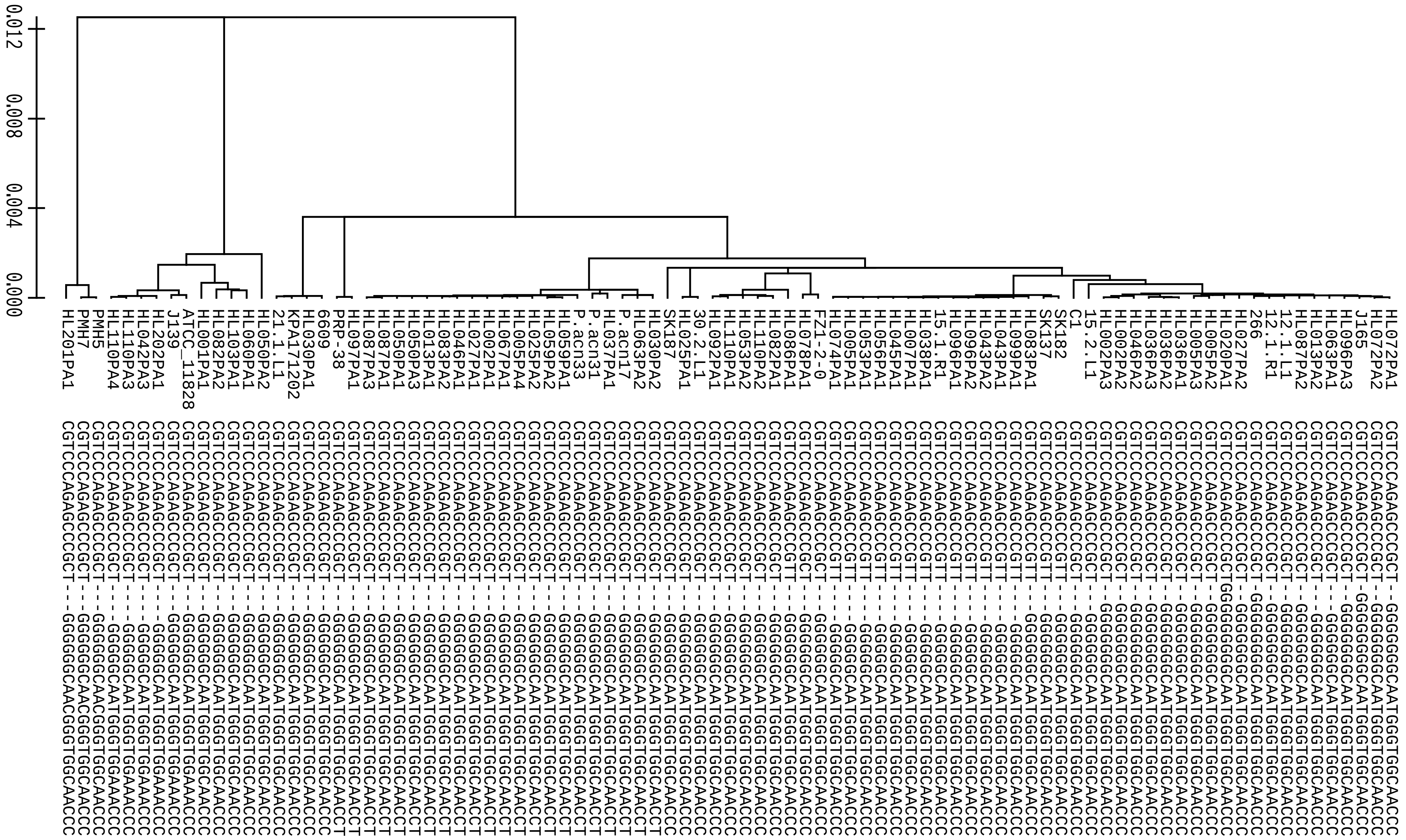

HPT#: HPT04
Gene: GntR family transcriptional regulator
PPA: PPA0165
Location: 199410:199437 (KPA171202)
Page: 4/54

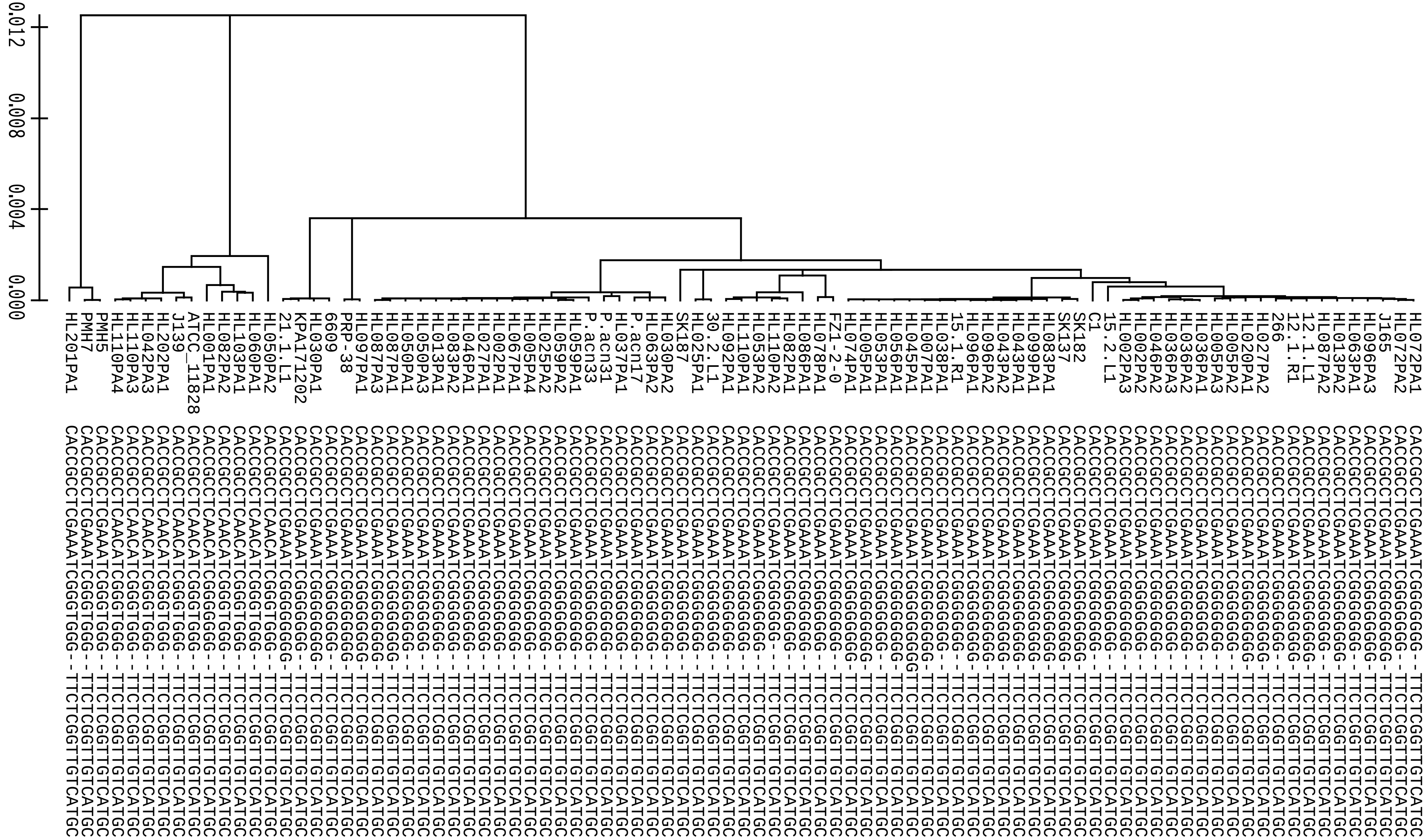

HPT#: HPT05

Gene: RHS-family protein / YD repeat protein

PPA: PPA0200

Location: 249739:249764 (KPA171202)

Page: 5/54

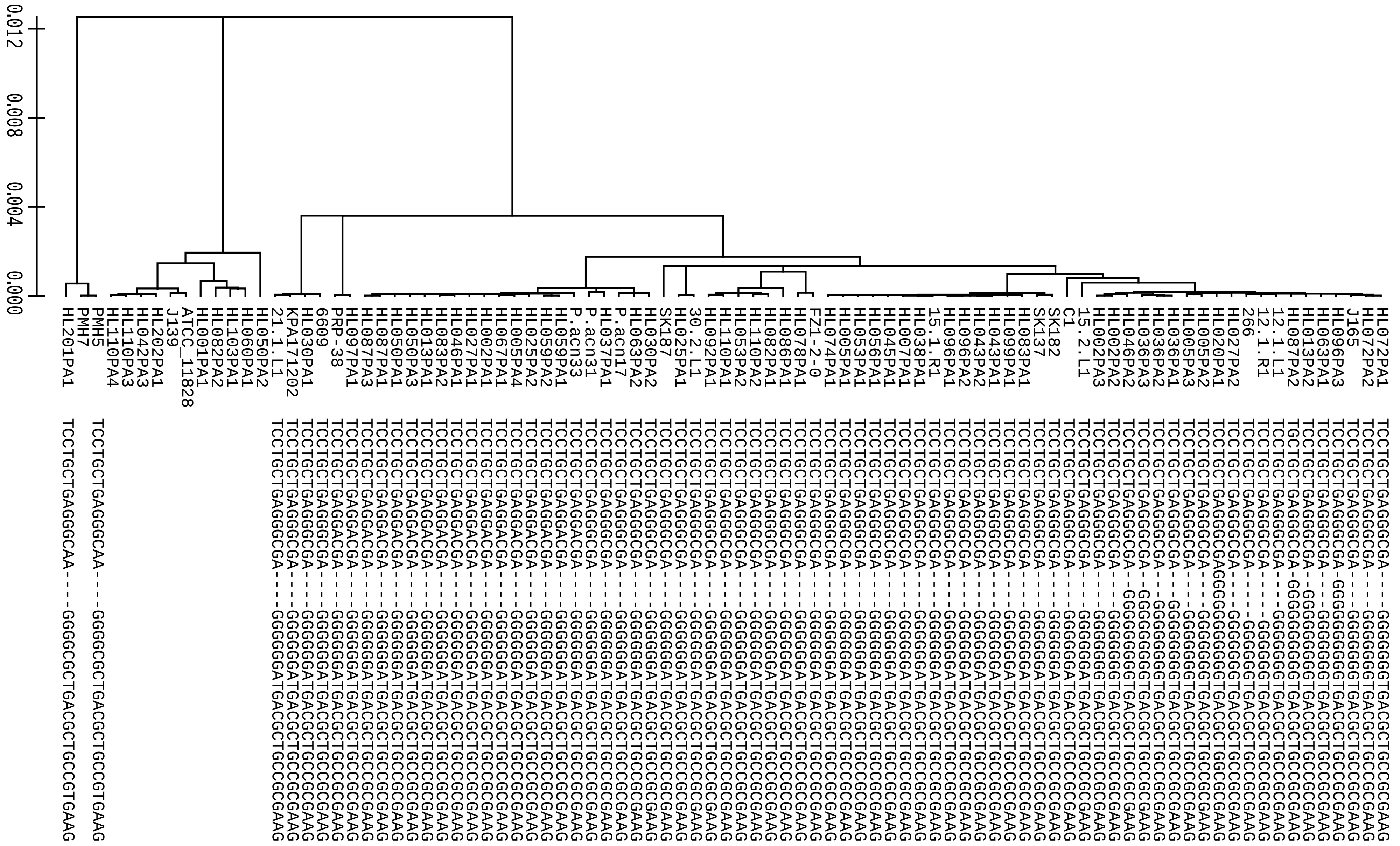

HPT#: HPT06
Gene: Putative oxidoreductase
PPA: PPA0378
Location: 424850:424877 (KPA171202)
Page: 6/54

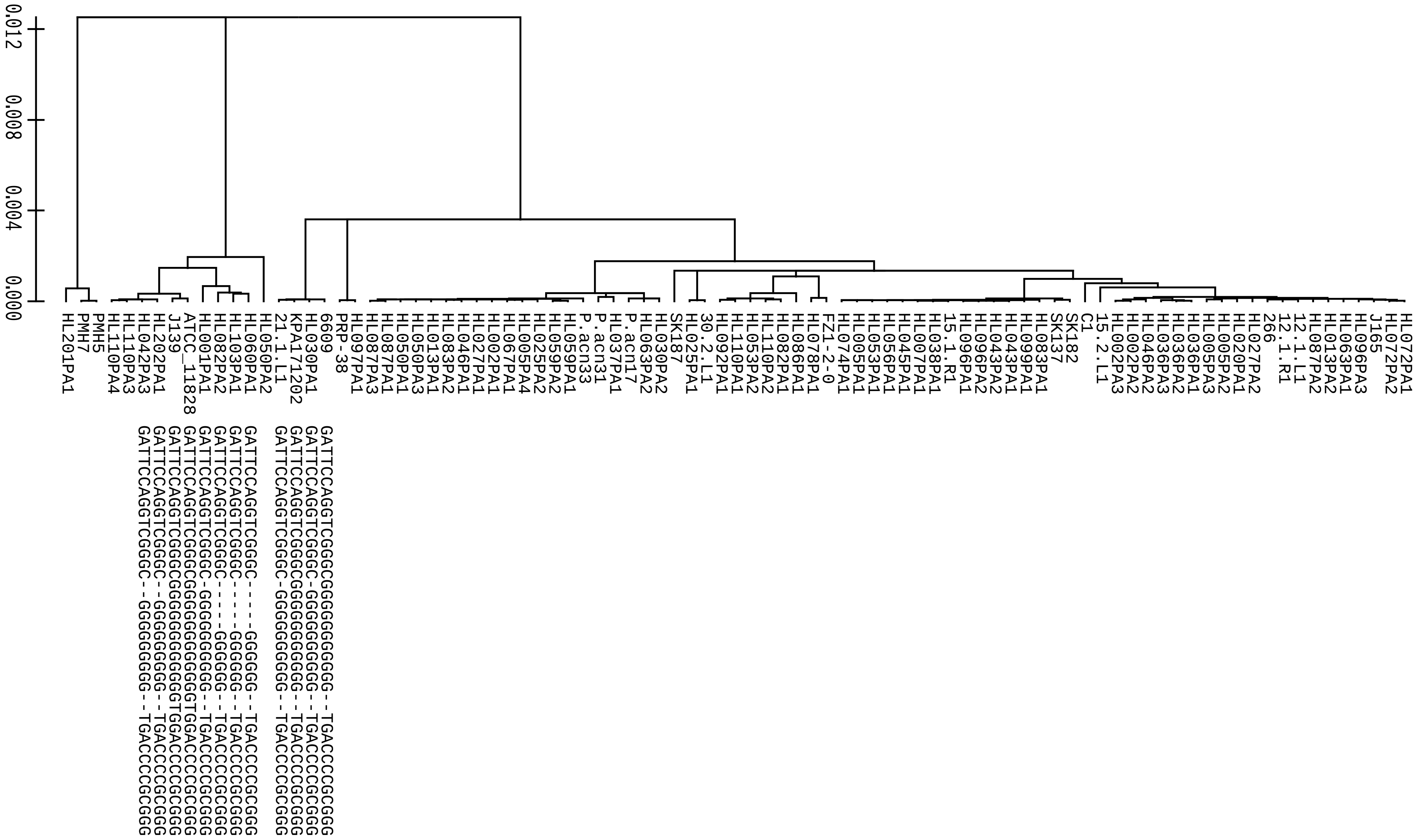

HPT#: HPT07
Gene: Hypothetical Protein
PPA: PPA0448
Location: 494973:494999 (KPA171202)
Page: 7/54

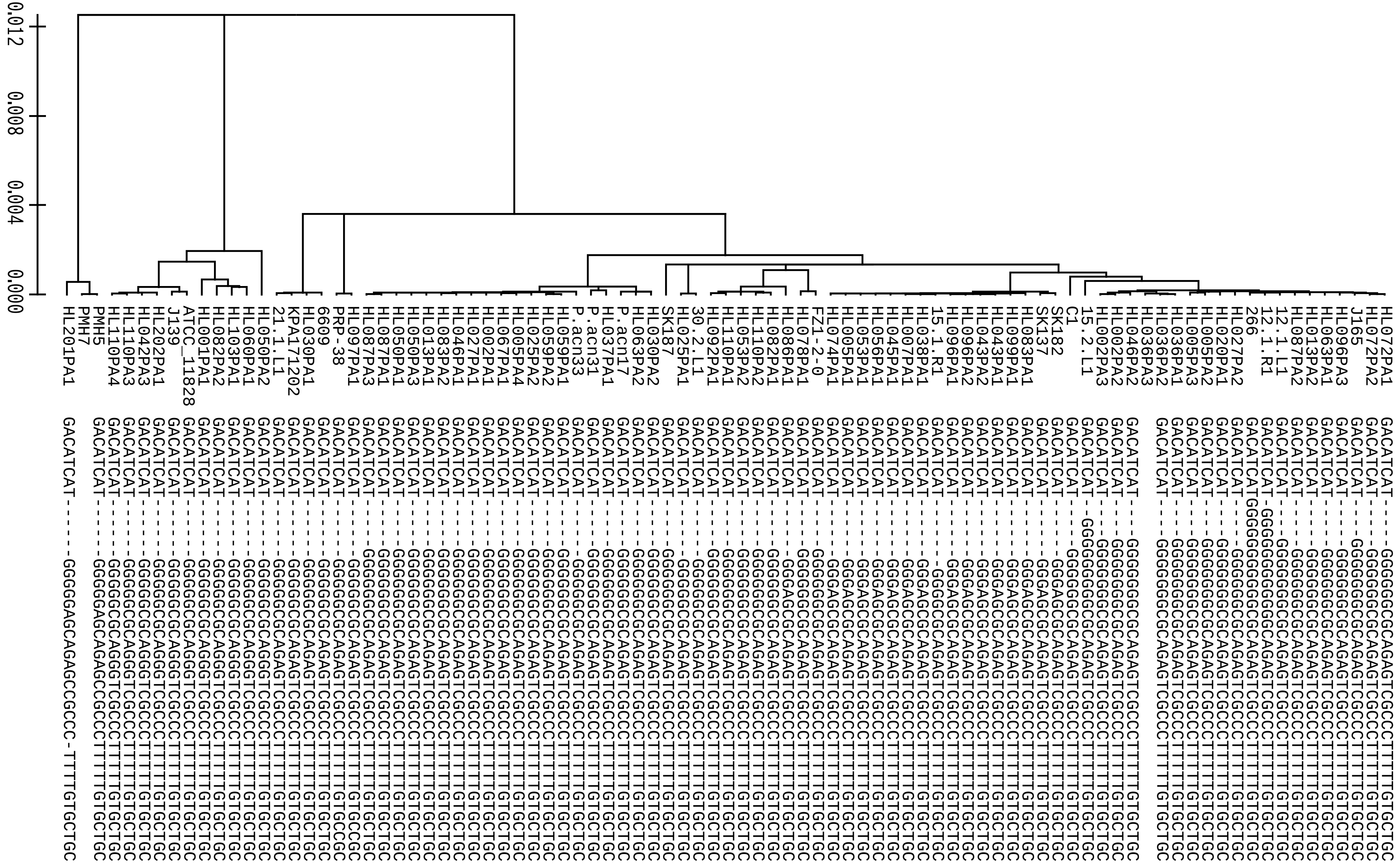

HPT#: HPT08
Gene: Molybdenum cofactor synthesis domain
PPA: PPA0513
Location: 565070:565097 (KPA171202)
Page: 8/54

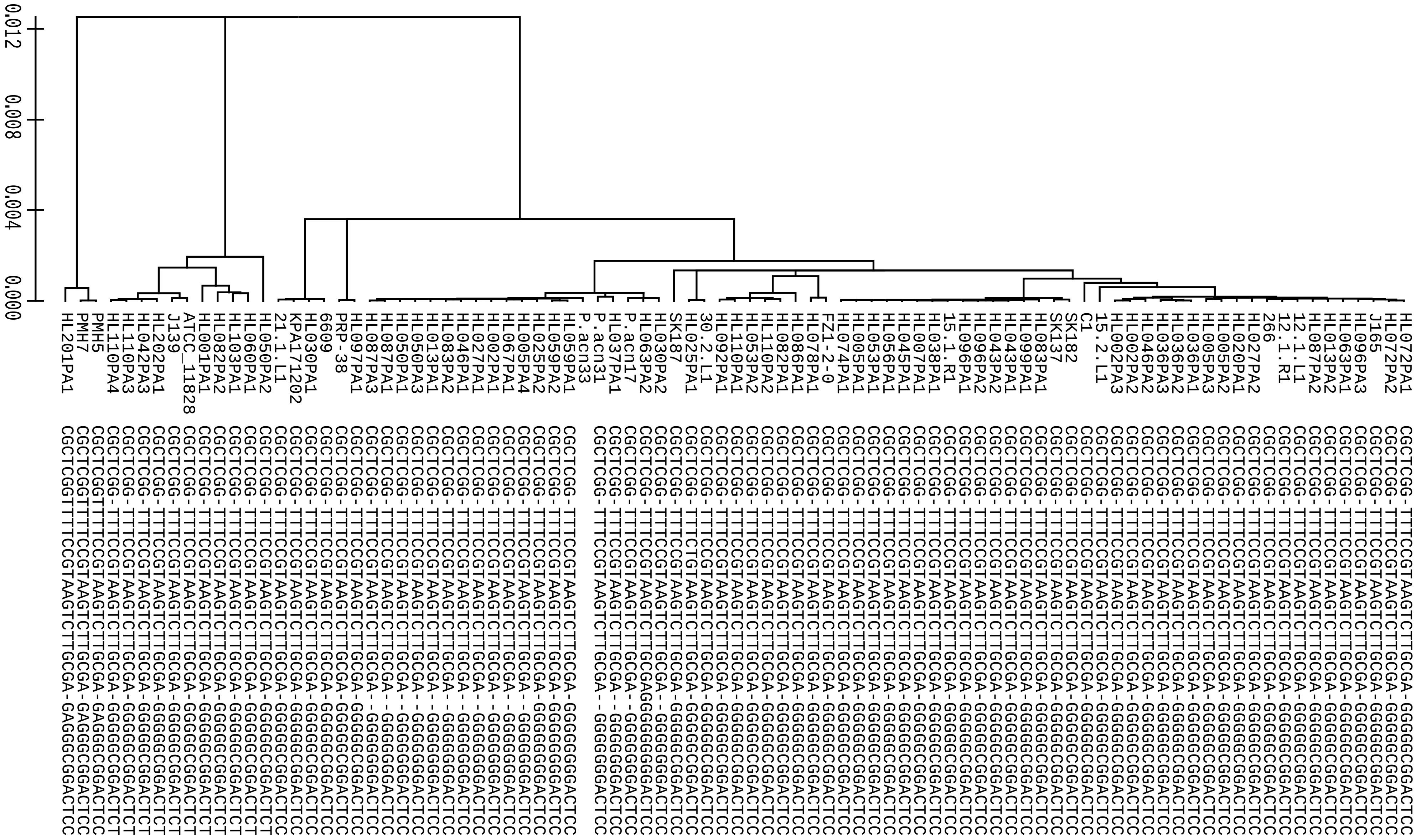

HPT#: HPT09
Gene: Sodium neurotransmitter symporter family
PPA: PPA0557
Location: 616529:616545 (KPA171202)
Page: 9/54

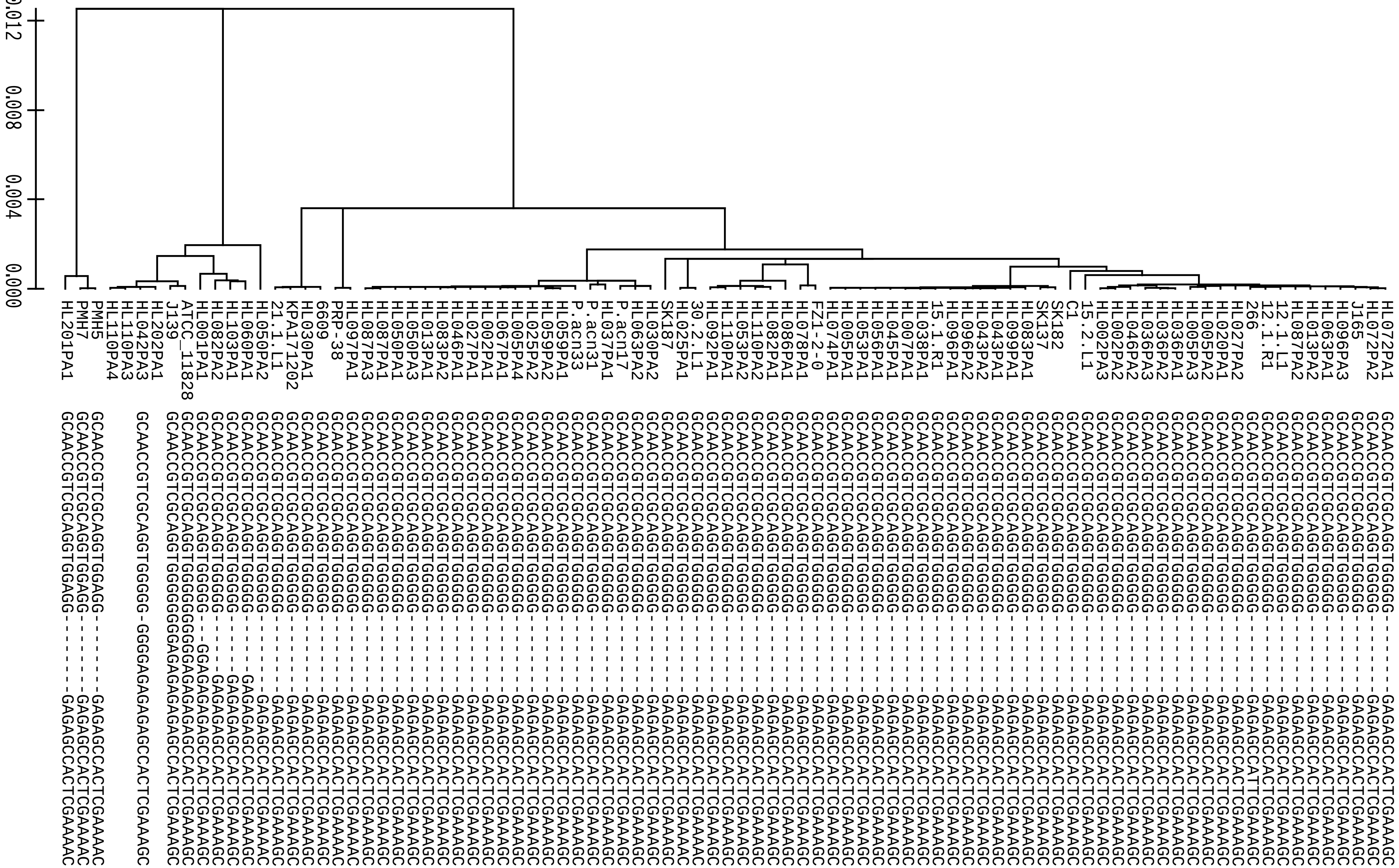

HPT#: HPT10
Gene: Putative hydrolase
PPA: PPA0735
Location: 808256:808279 (KPA171202)
Page: 10/54

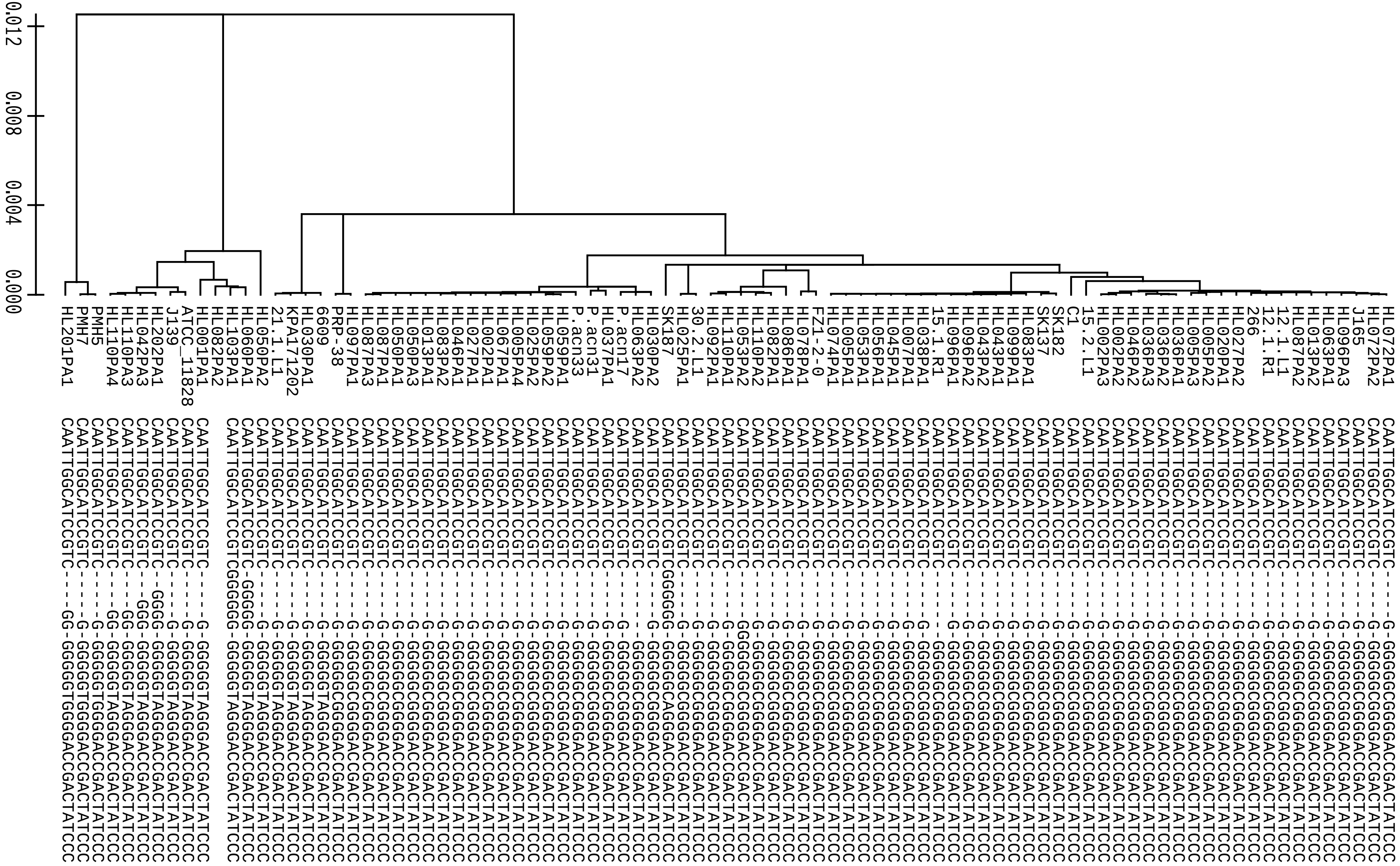

HPT#: HPT11
Gene: HtaA domain, Putative Fe-transport
PPA: PPA0779
Location: 856169:856202 (KPA171202)
Page: 11/54

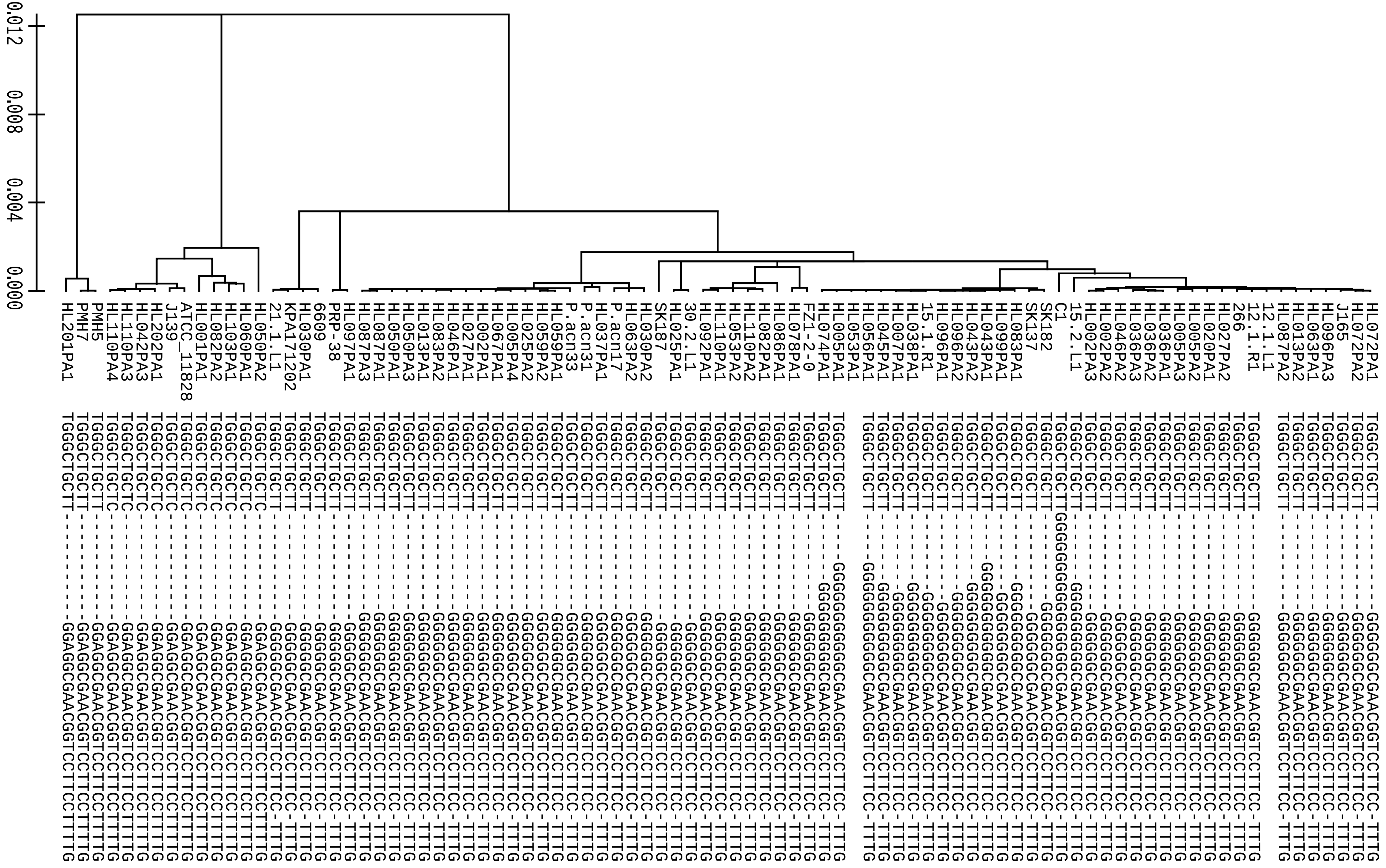

HPT#: HPT12

Gene: CobN/magnesium chelatase subunit

PPA: PPA0791

Location: 873556:873582 (KPA171202)

Page: 12/54

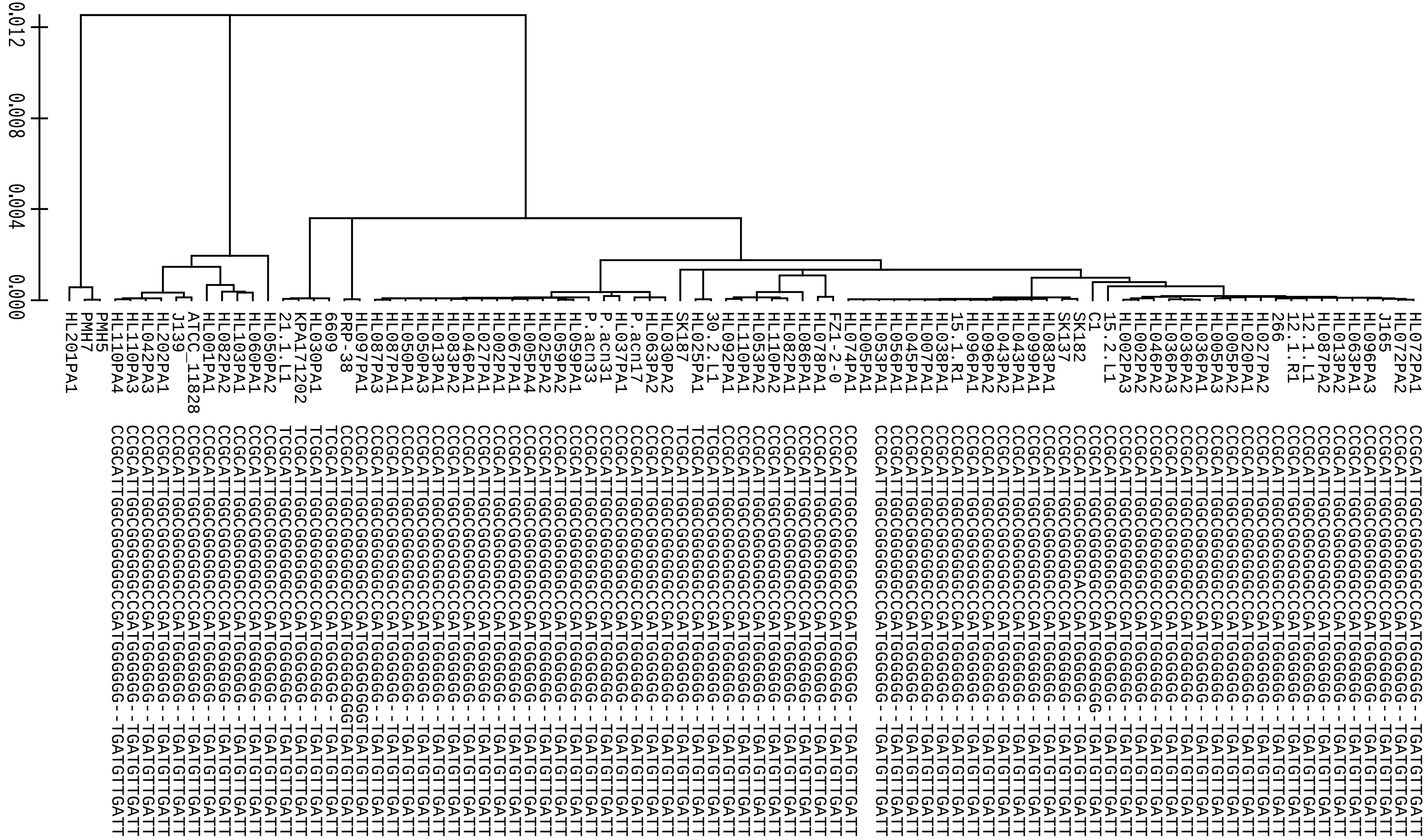

HPT#: HPT13
Gene: CobN/magnesium chelatase subunit
PPA: PPA0791
Location: 874120:874148 (KPA171202)
Page: 13/54

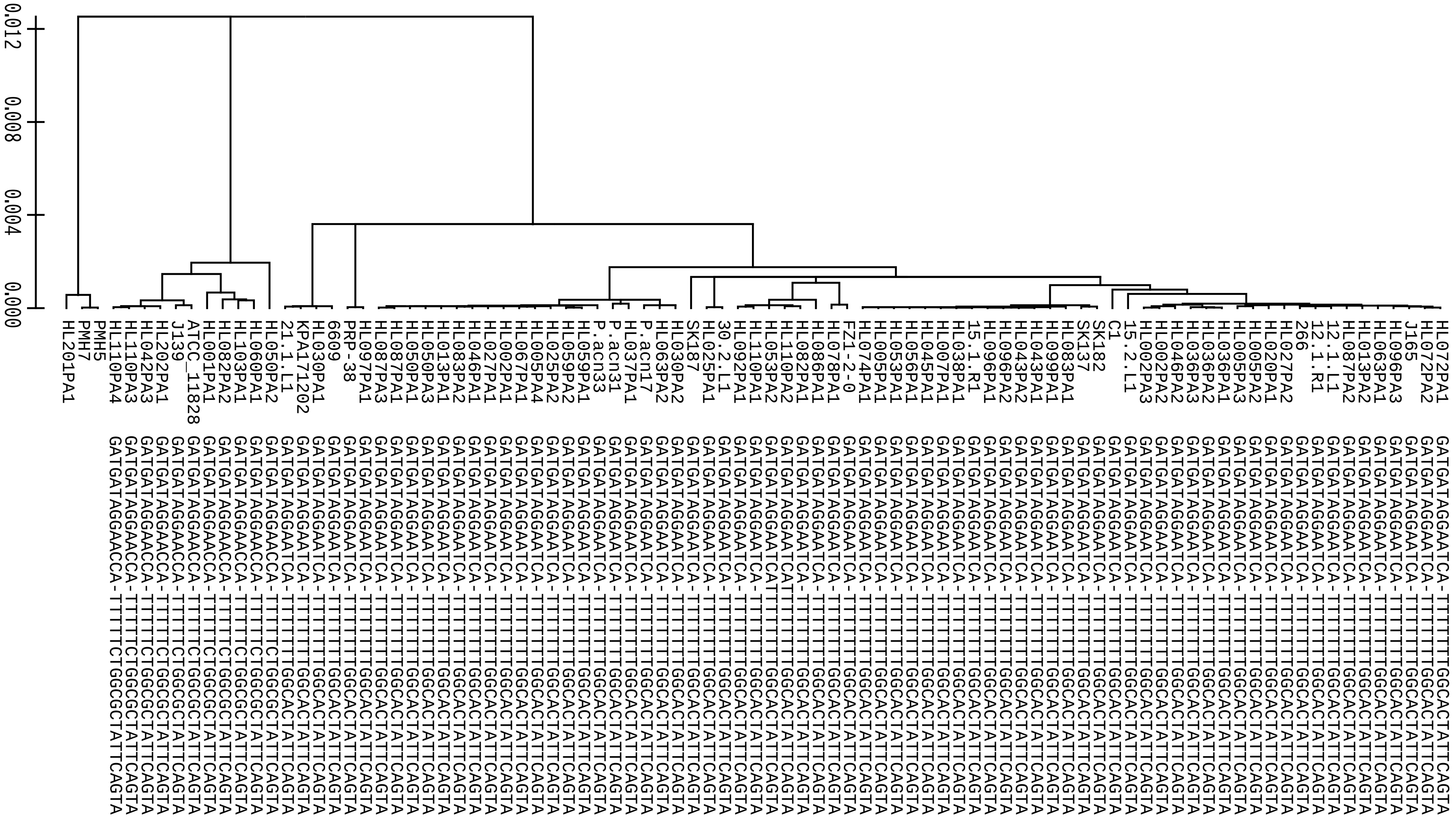

HPT#: HPT14
Gene: GntR family transcriptional regulator
PPA: PPA0875
Location: 950951:950975 (KPA171202)
Page: 14/54

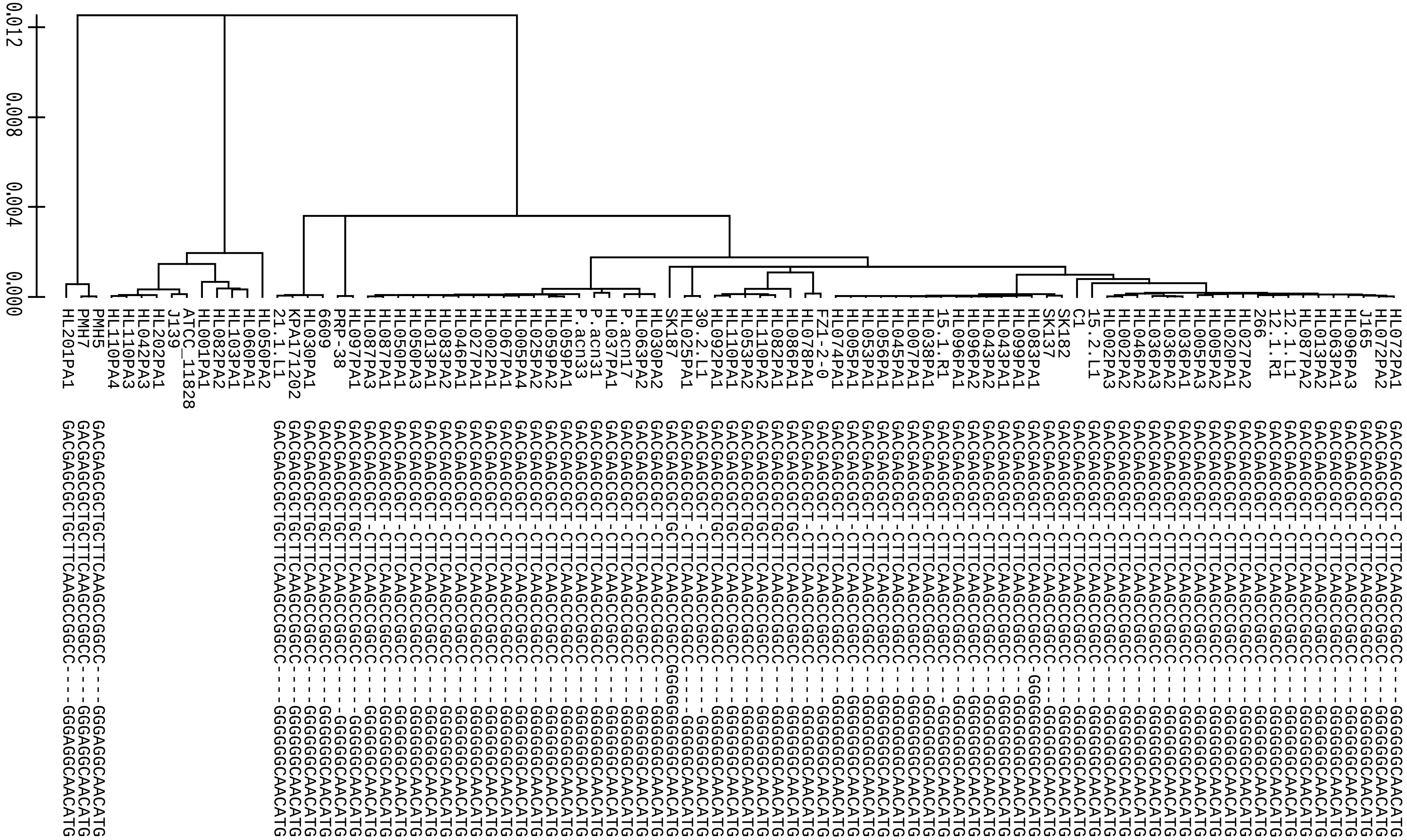

HPT#: HPT15
Gene: putative beta-glycosidase fragment
PPA: PPA0992
Location: 1076736:1076761 (KPA171202)
Page: 15/54

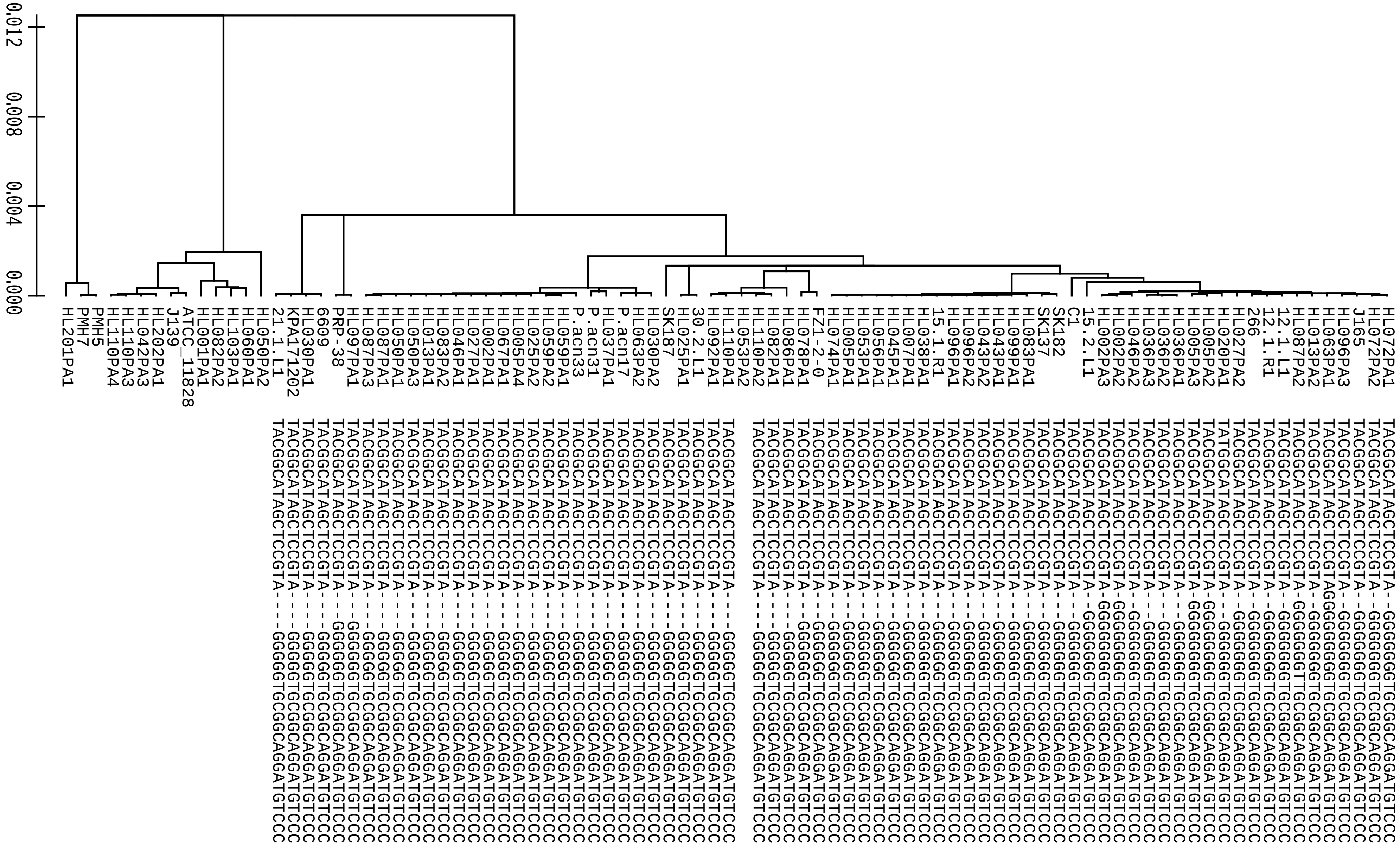

HPT#: HPT16
Gene: Acetyltransferase, GNAT family
PPA: PPA1040
Location: 1131631:1131663 (KPA171202)
Page: 16/54

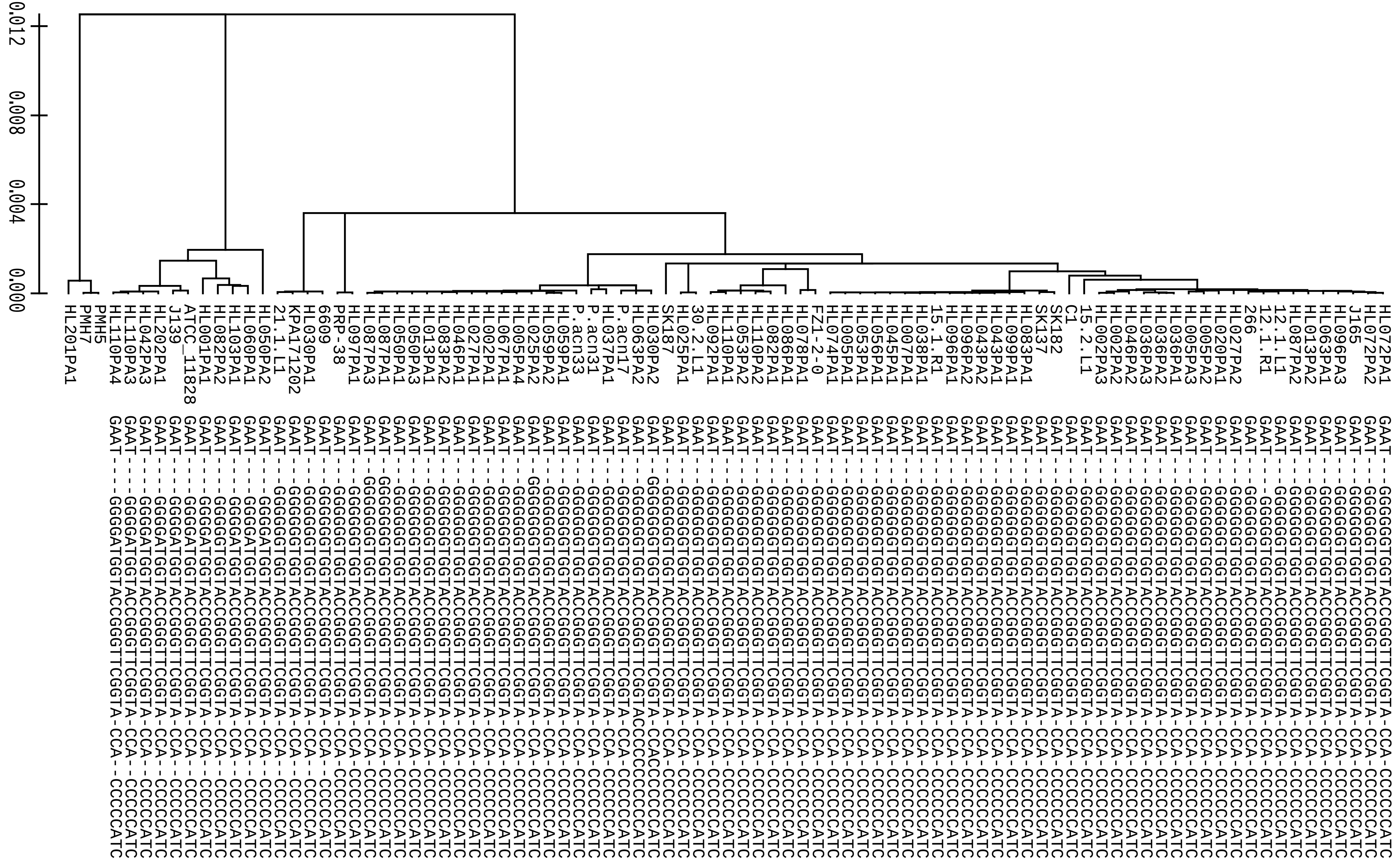

HPT#: HPT17
Gene: Molecular chaperone / BnaK domain
PPA: PPA1098
Location: 1189744:1189768 (KPA171202)
Page: 17/54

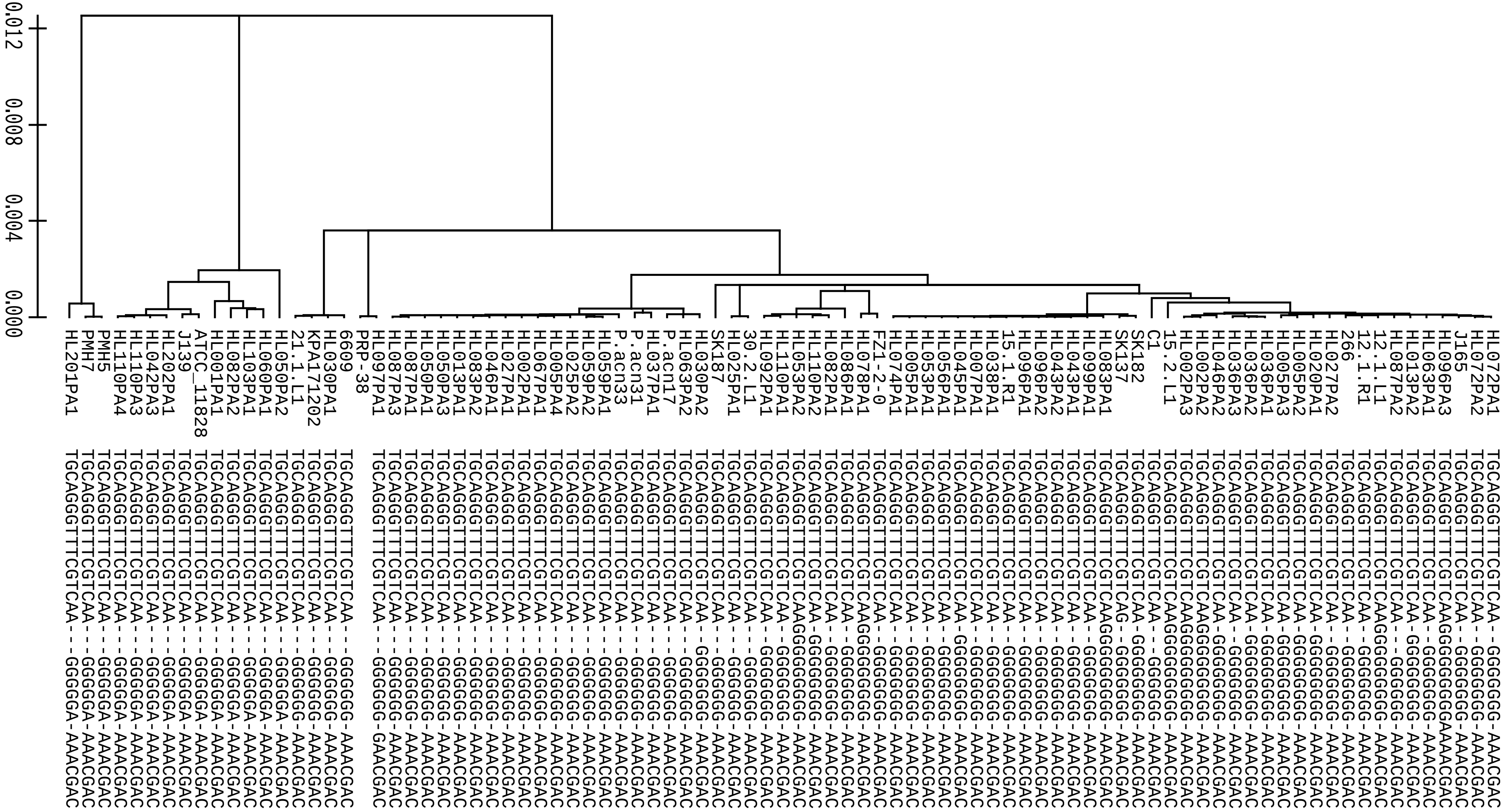

HPT#: HPT18
Gene: Putative sensor histidine kinase PdtaS
PPA: PPA1265
Location: 1372989:1373014 (KPA171202)
Page: 18/54

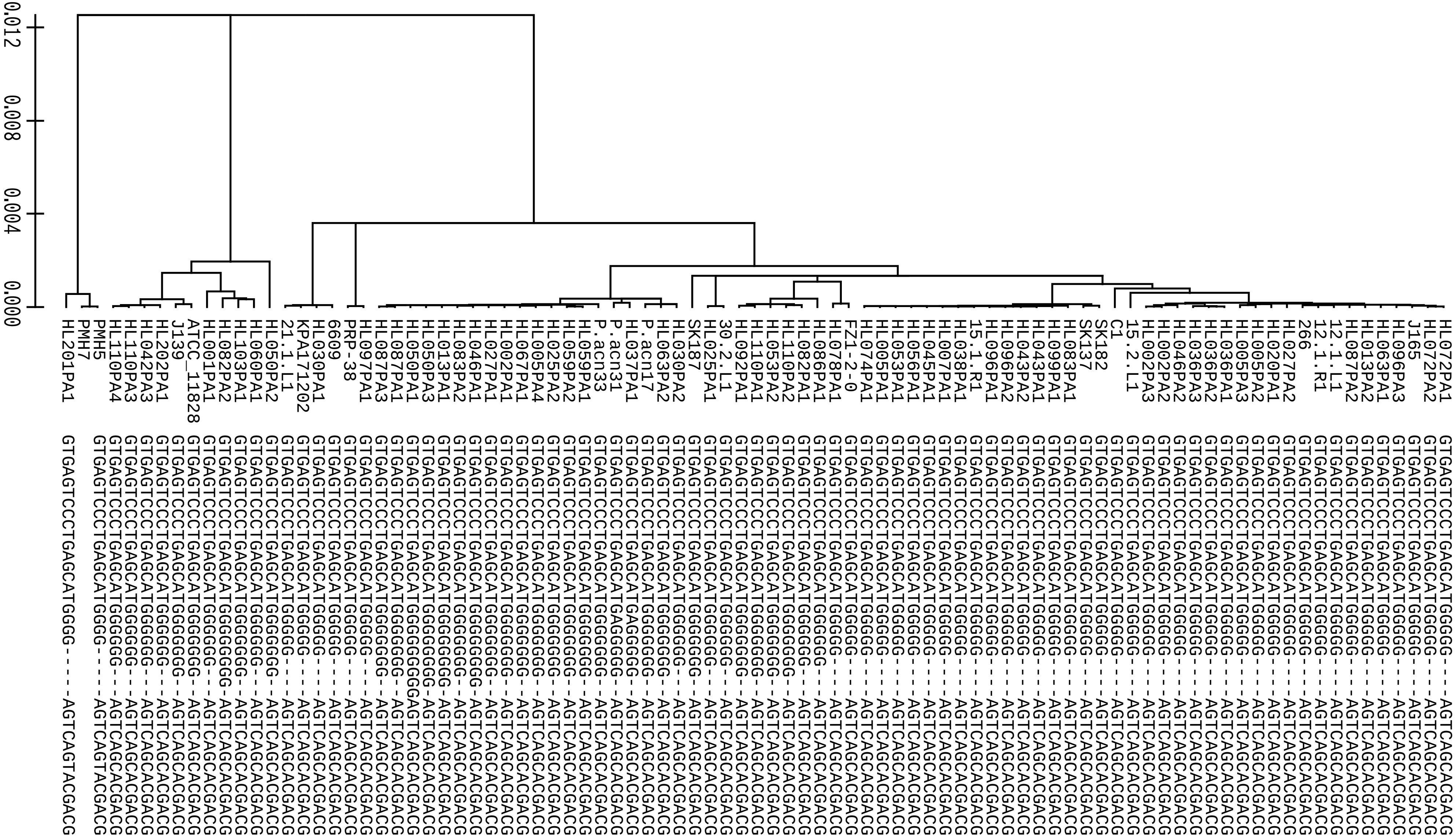

HPT#: HPT19

Gene: Hypothetical Protein

PPA: PPA1294

Location: 1406560:1406593 (KPA171202)

Page: 19/54

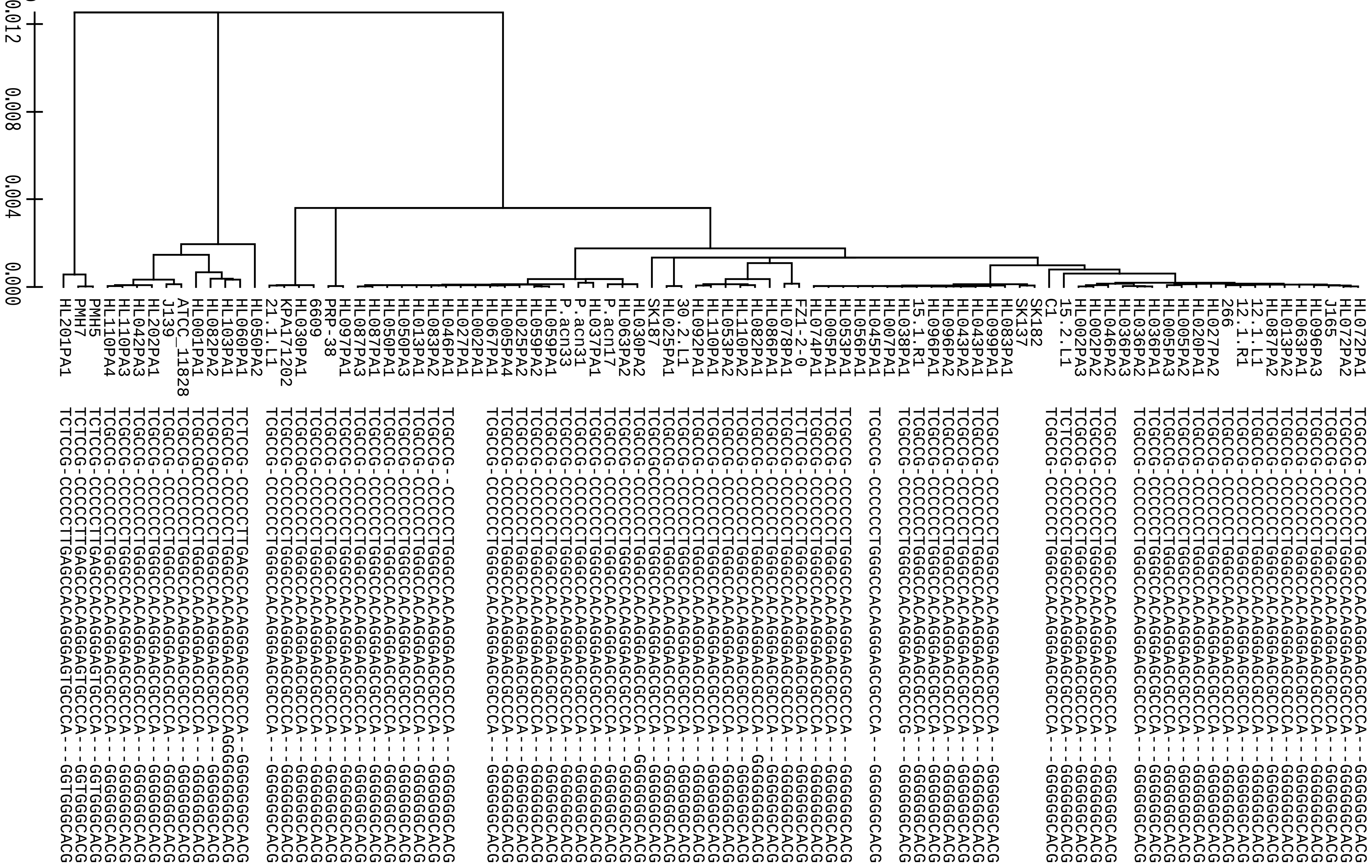

HPT#: HPT20

Gene: Preprotein translocase SecA subunit

PPA: PPA1333

Location: 1455191:1455211 (KPA171202)

Page: 20/54

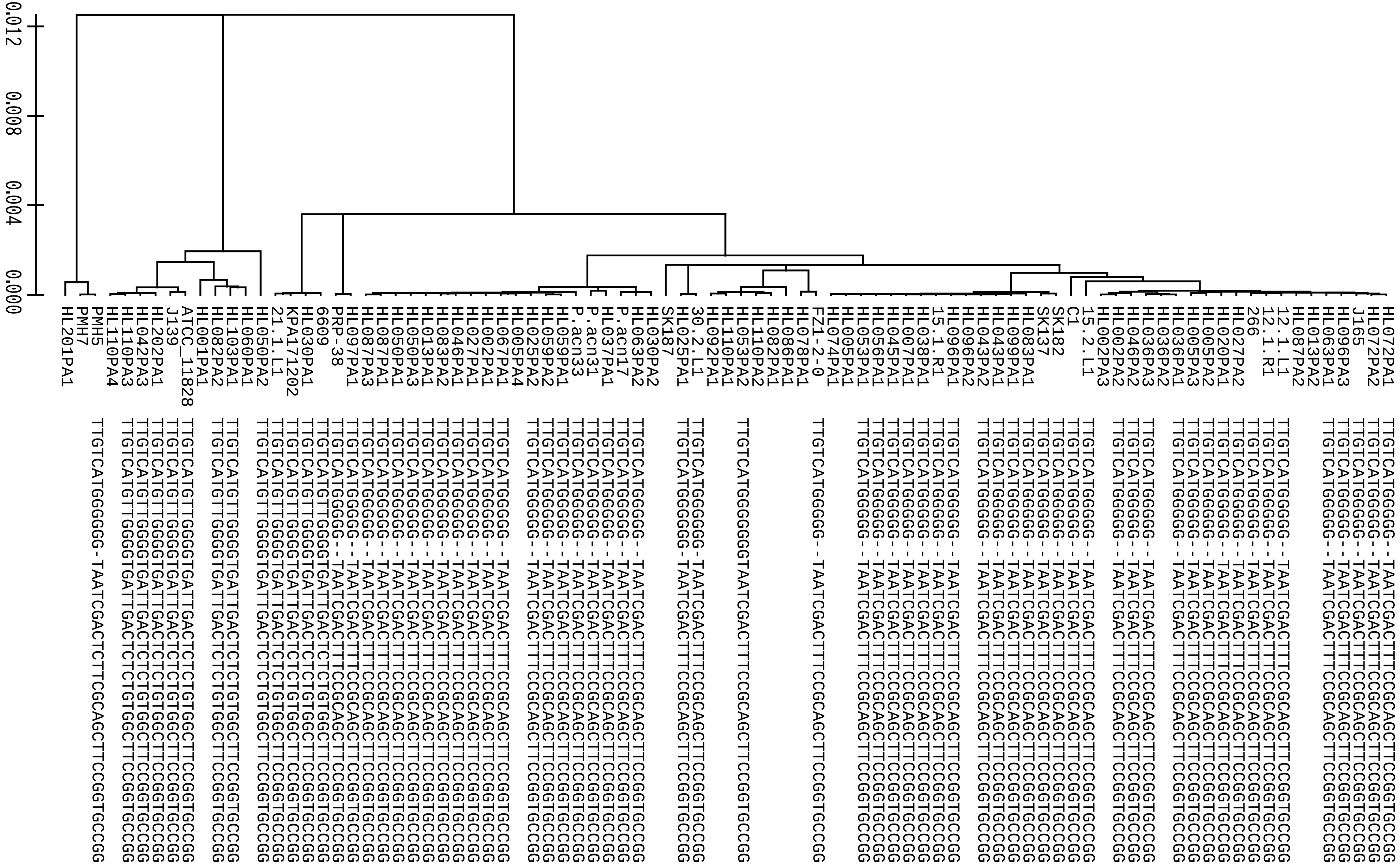

HPT#: HPT21

Gene: 3-isopropylmalate dehydratase

PPA: PPA1361

Location: 1485771:1485819 (KPA171202)

Page: 21/54

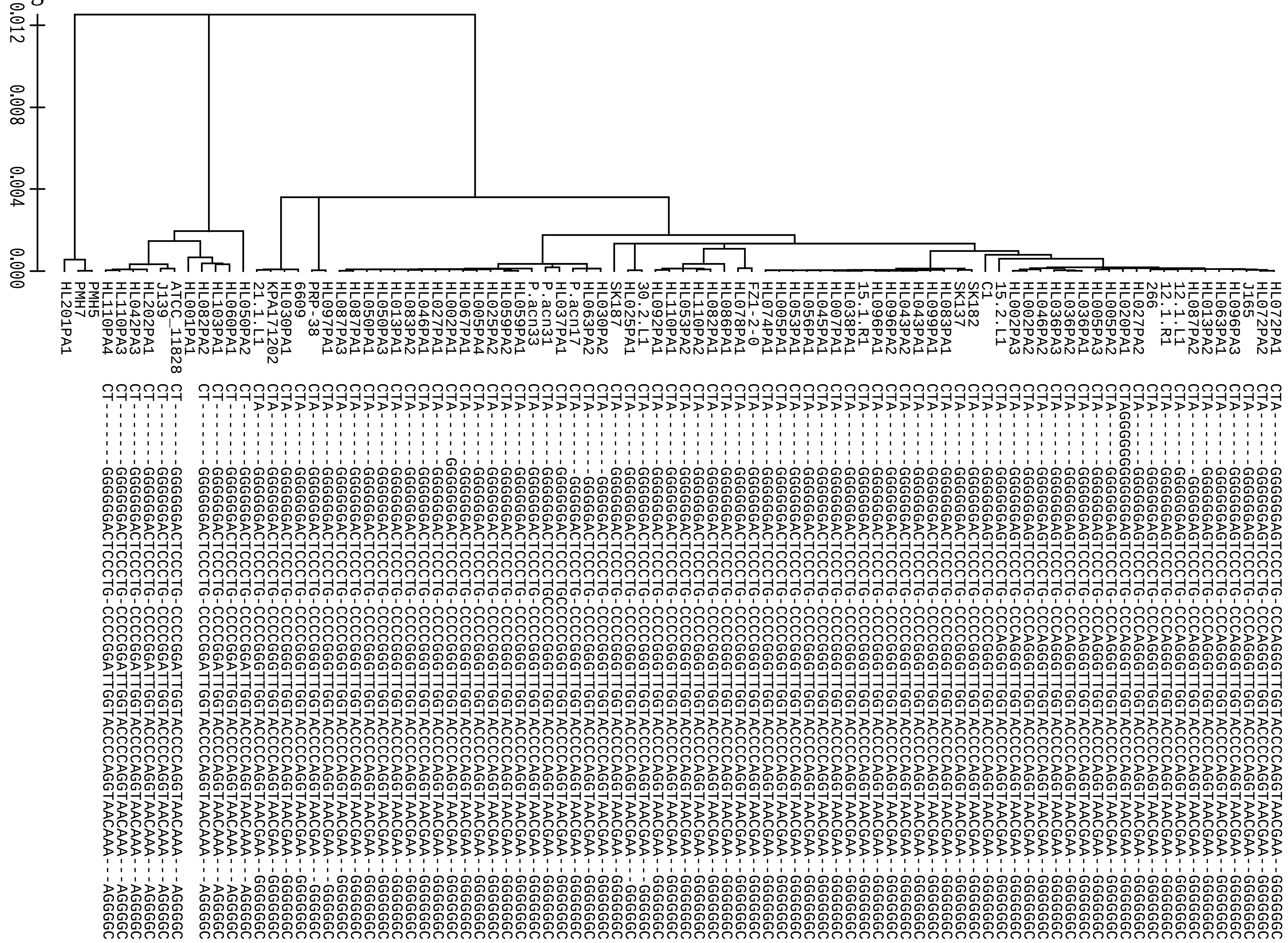

HPT#: HPT22
Gene: 50S ribosomal protein L20
PPA: PPA1412
Location: 1535003:1535026 (KPA171202)
Page: 22/54

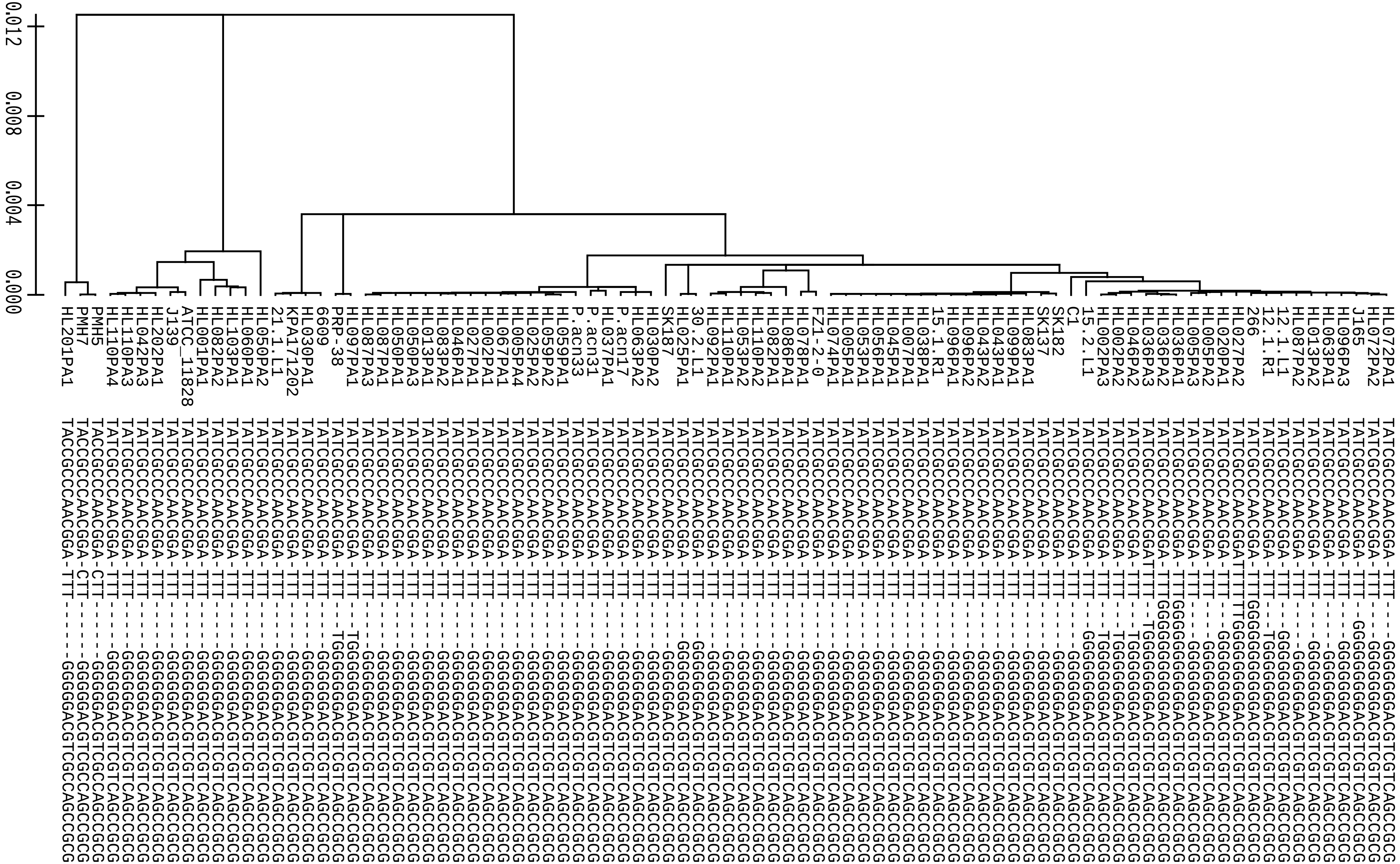

HPT#: HPT23
Gene: translation initiation factor IF-2
PPA: PPA1493
Location: 1612804:1612831 (KPA171202)
Page: 23/54

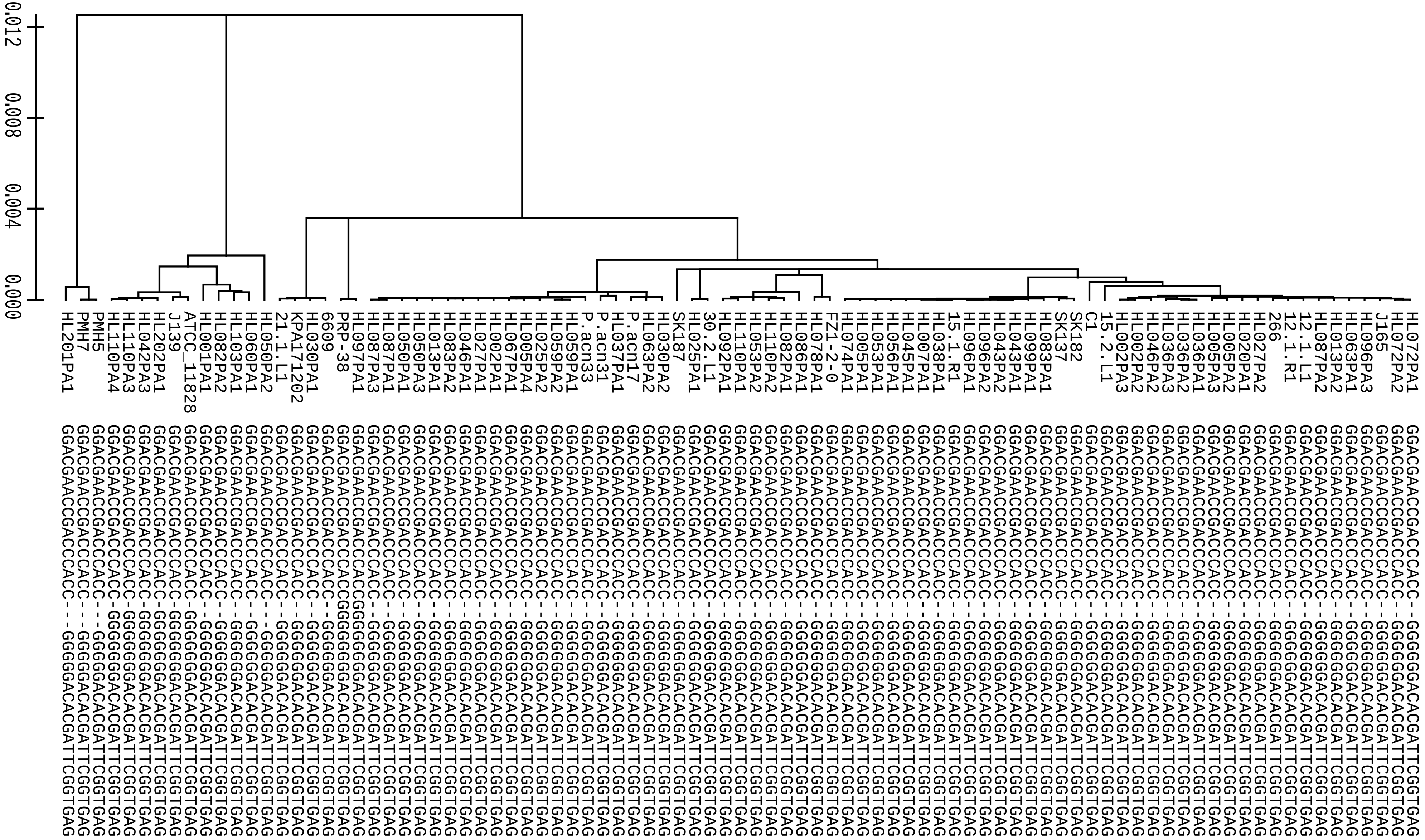

HPT#: HPT24
Gene: Hypothetical Protein
PPA: PPA1537
Location: 1657456:1657481 (KPA171202)
Page: 24/54

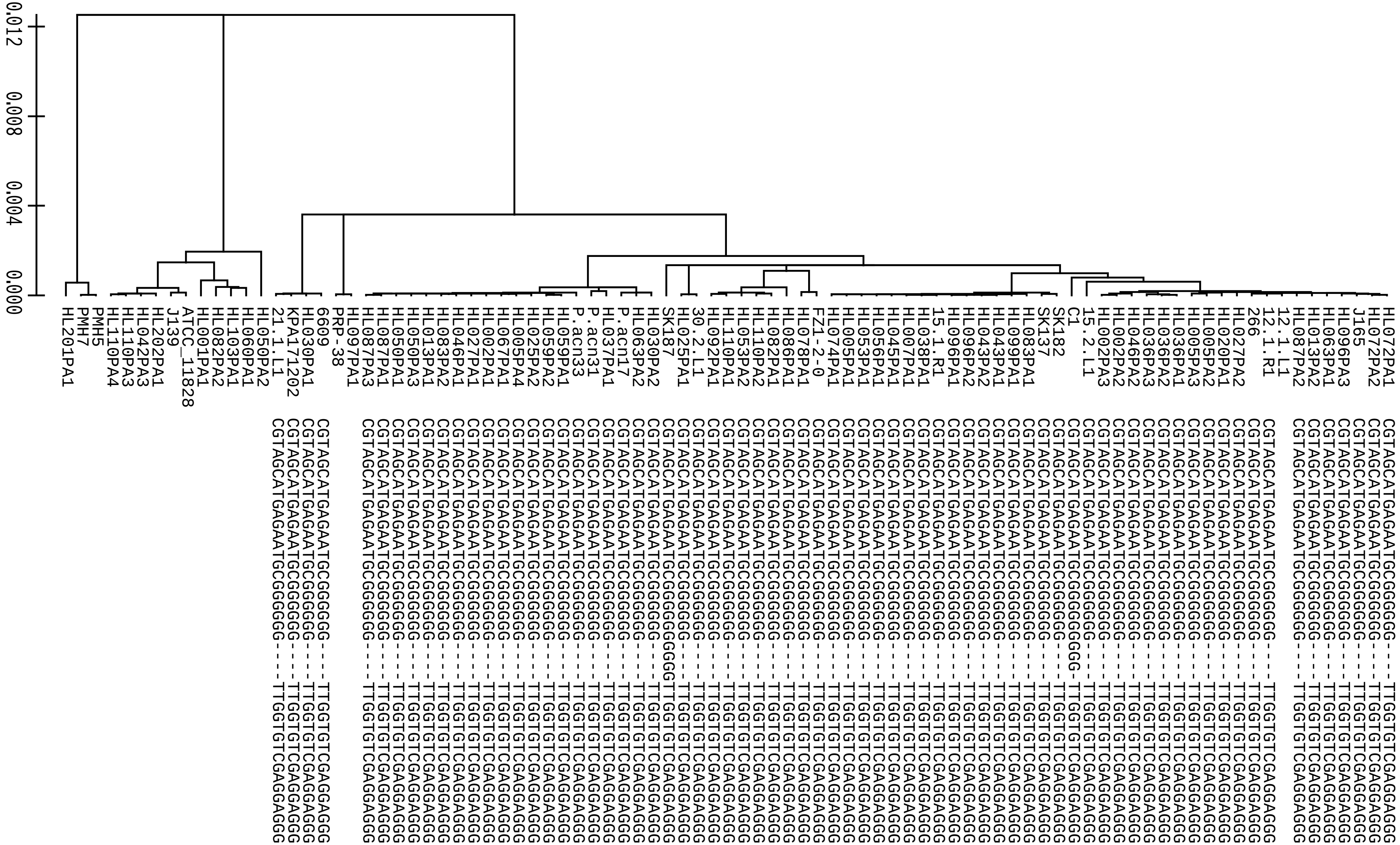

HPT#: HPT25

Gene: D-serine/D-alanine/glycine transporter

PPA: PPA1643

Location: 1789777:1789810 (KPA171202)

Page: 25/54

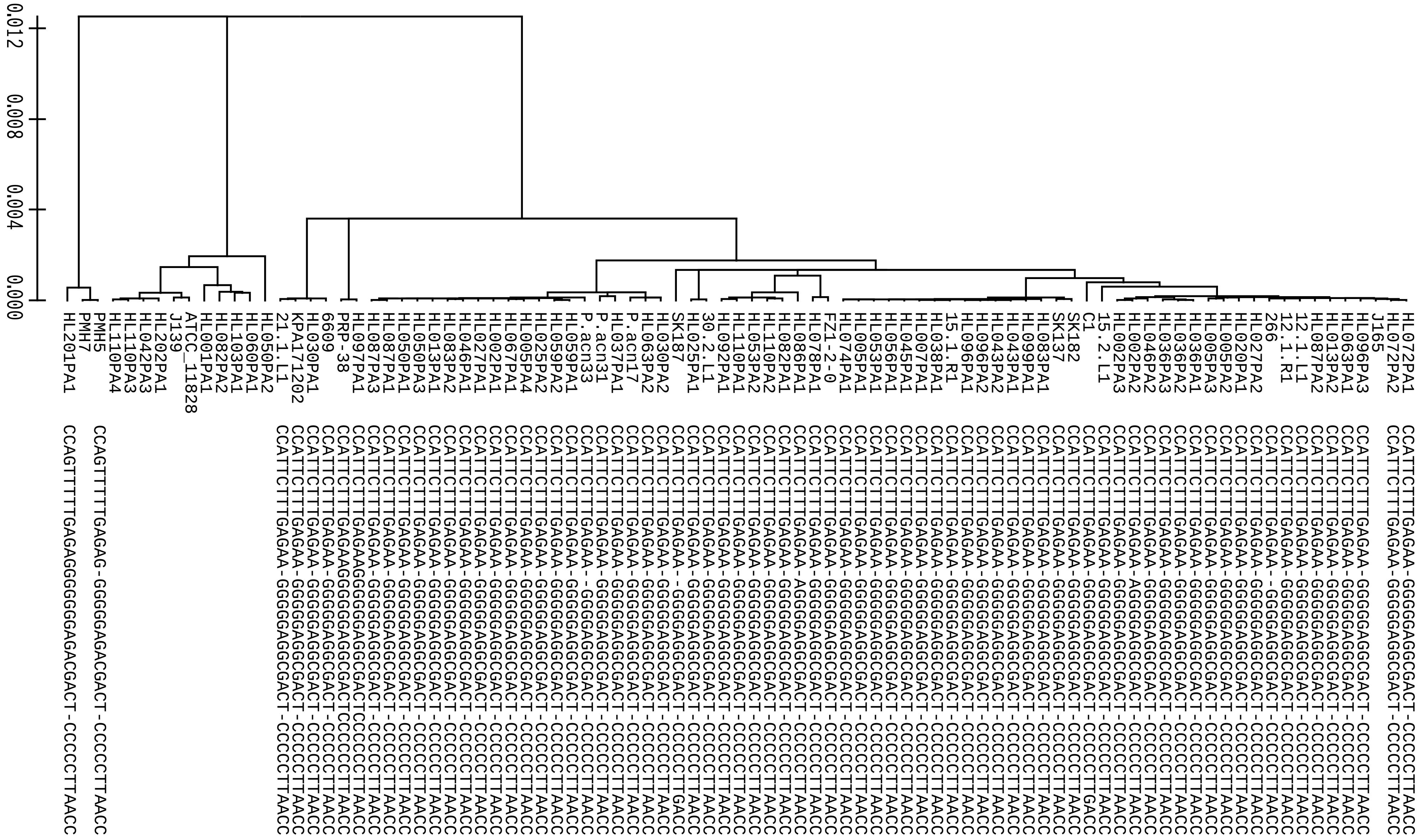

HPT#: HPT26
Gene: Glycosyl hydrolase family 25/lysozyme M1
PPA: PPA1662
Location: 1815548:1815584 (KPA171202)
Page: 26/54

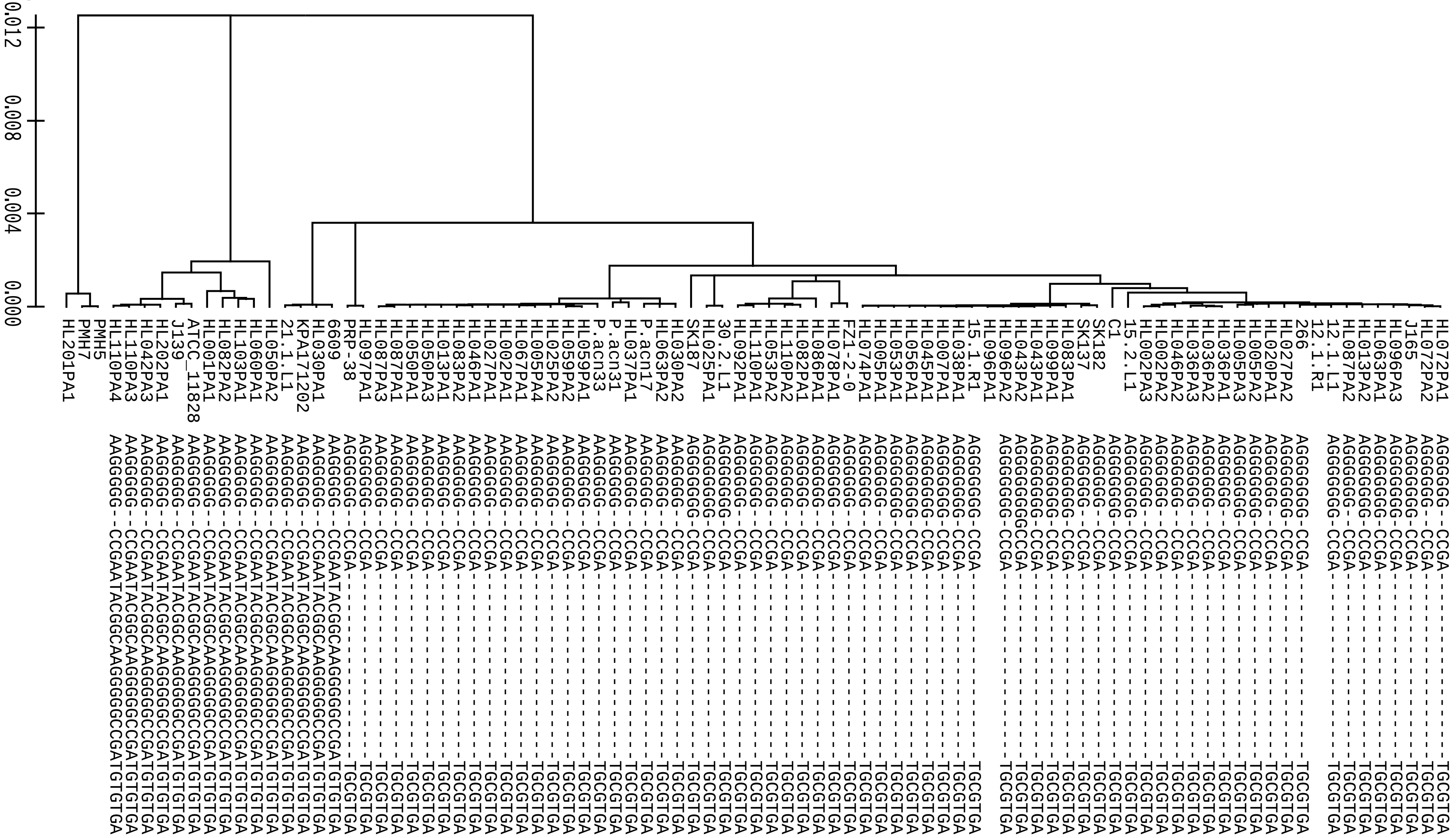

HPT#: HPT27

Gene: Putative magnesium/cobolt transport CorA

PPA: PPA1716

Location: 1869600:1869634 (KPA171202)

Page: 27/54

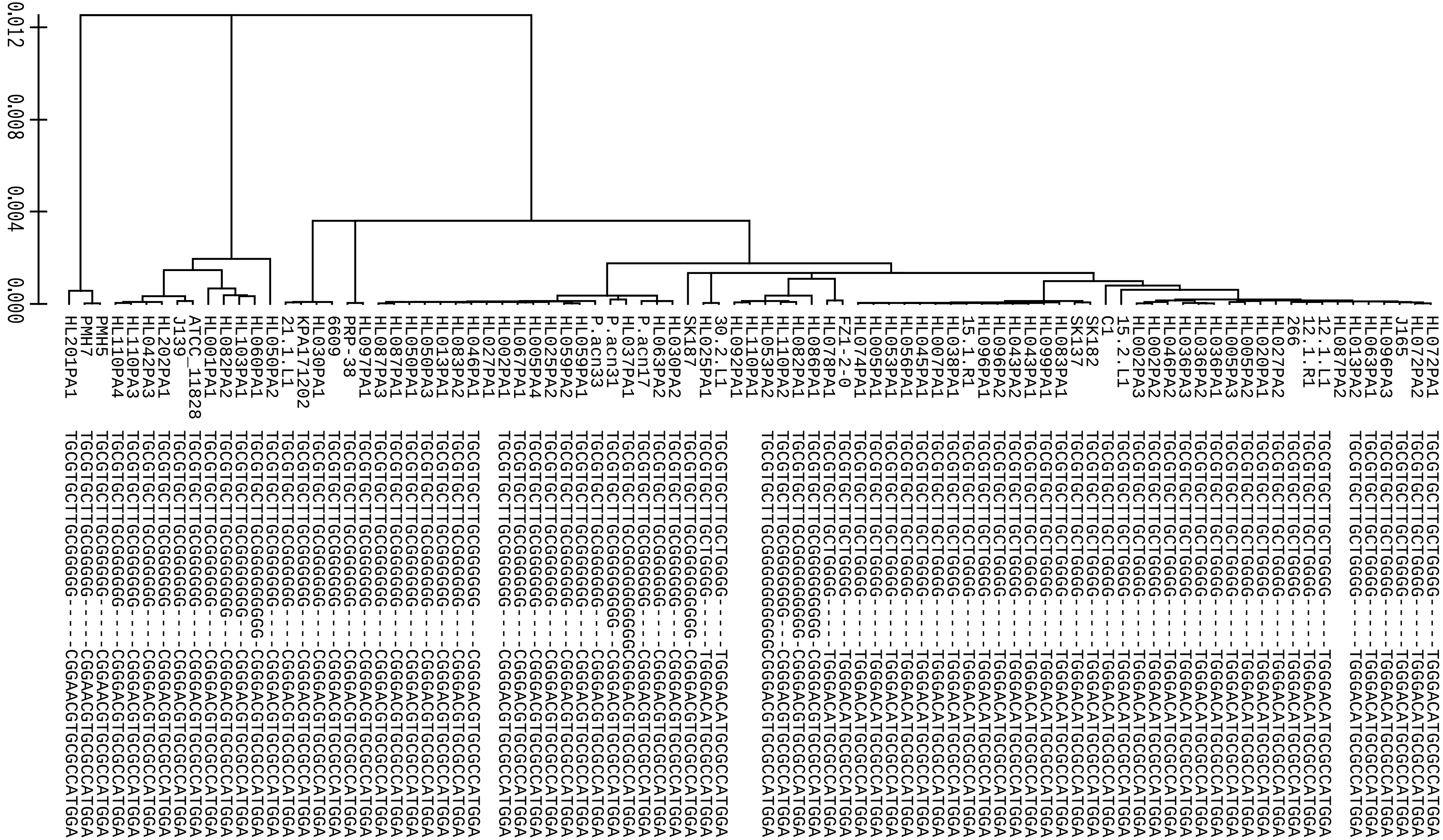

HPT#: HPT28
Gene: Cardiolipin synthetase/phospholipase D
PPA: PPA1761
Location: 1924263:1924286 (KPA171202)
Page: 28/54

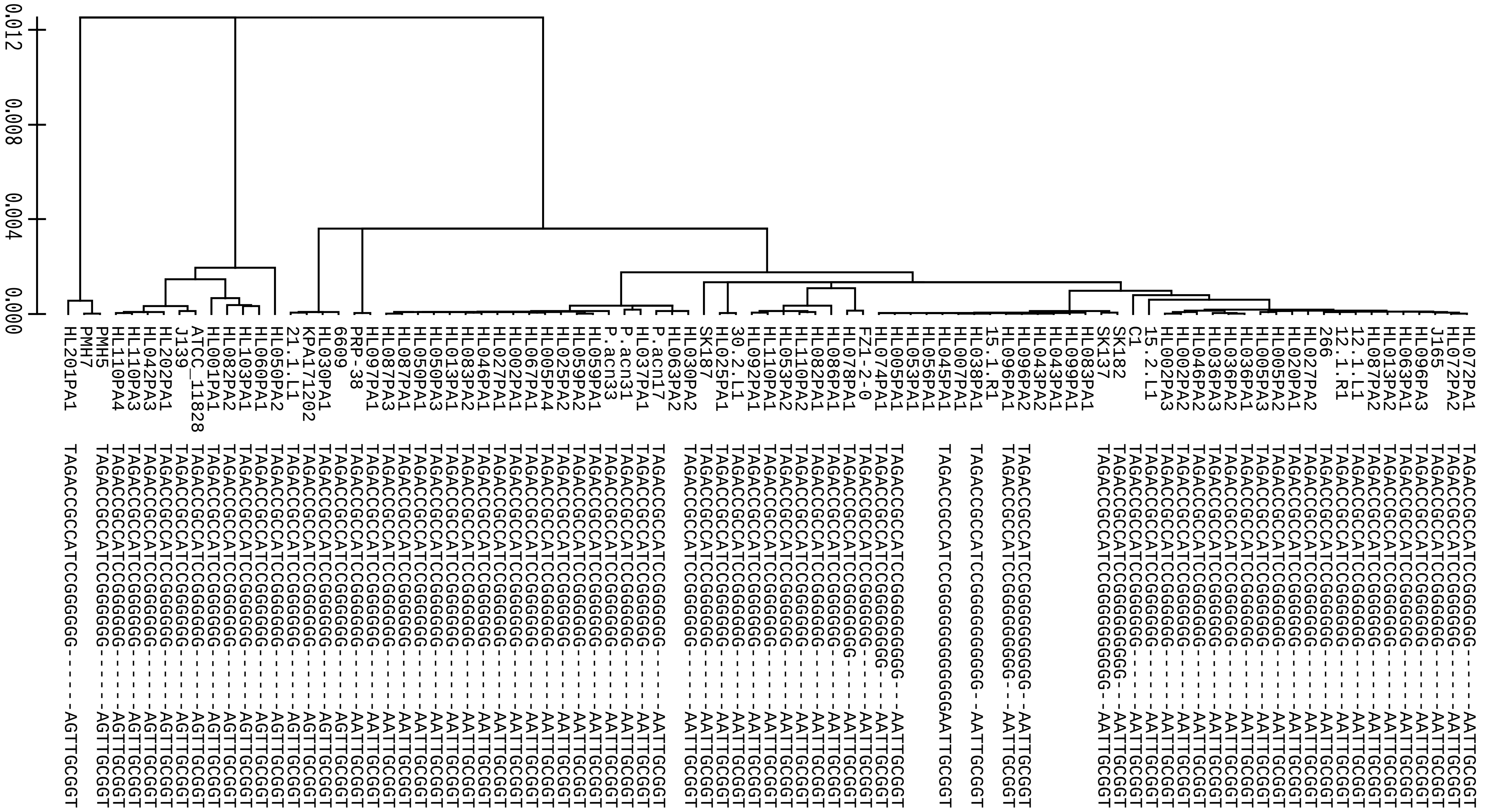

HPT#: HPT29
Gene: Hypothetical Protein
PPA: PPA1771
Location: 1935888:1935911 (KPA171202)
Page: 29/54

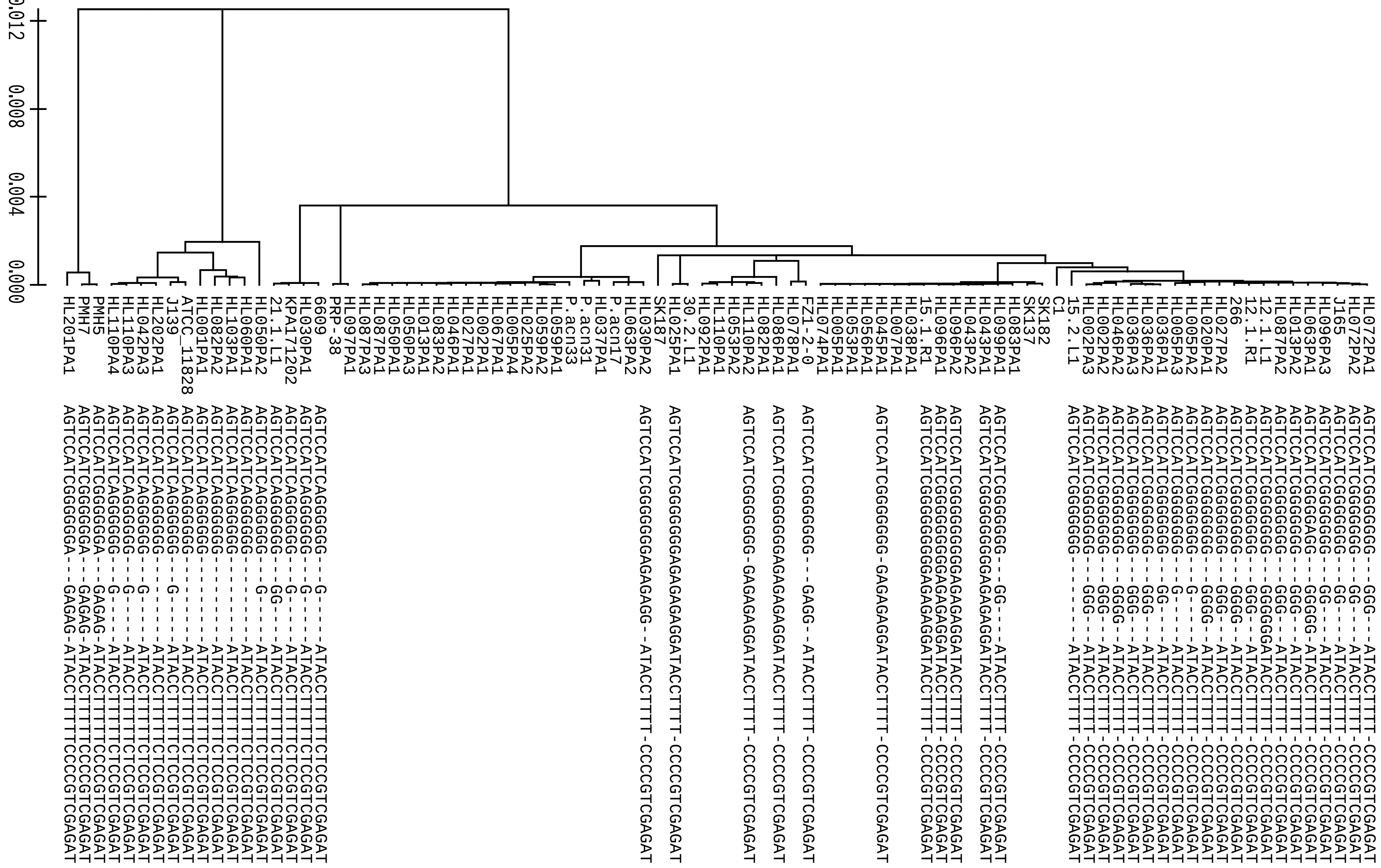

HPT#: HPT30
Gene: Putative holo-[acyl-carrier-protein] synthase, or 4'-phosphopantetheinyl transferase
PPA: PPA1793
Location: 1958937:1958964 (KPA171202)
Page: 30/54

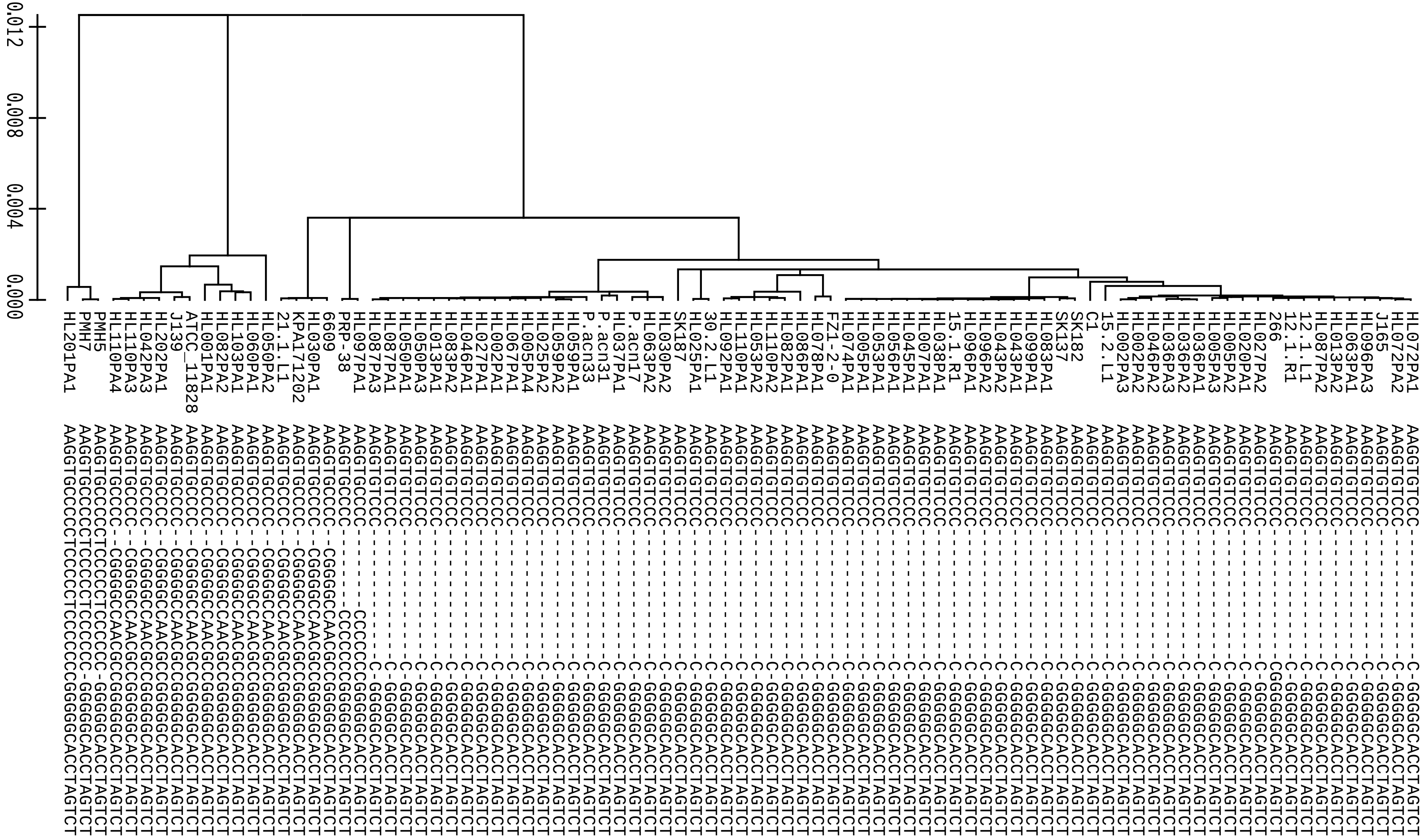

HPT#: HPT31

Gene: Endo-beta-N-acetylglucosaminidase family

PPA: PPA1805

Location: 1967962:1967986 (KPA171202)

Page: 31/54

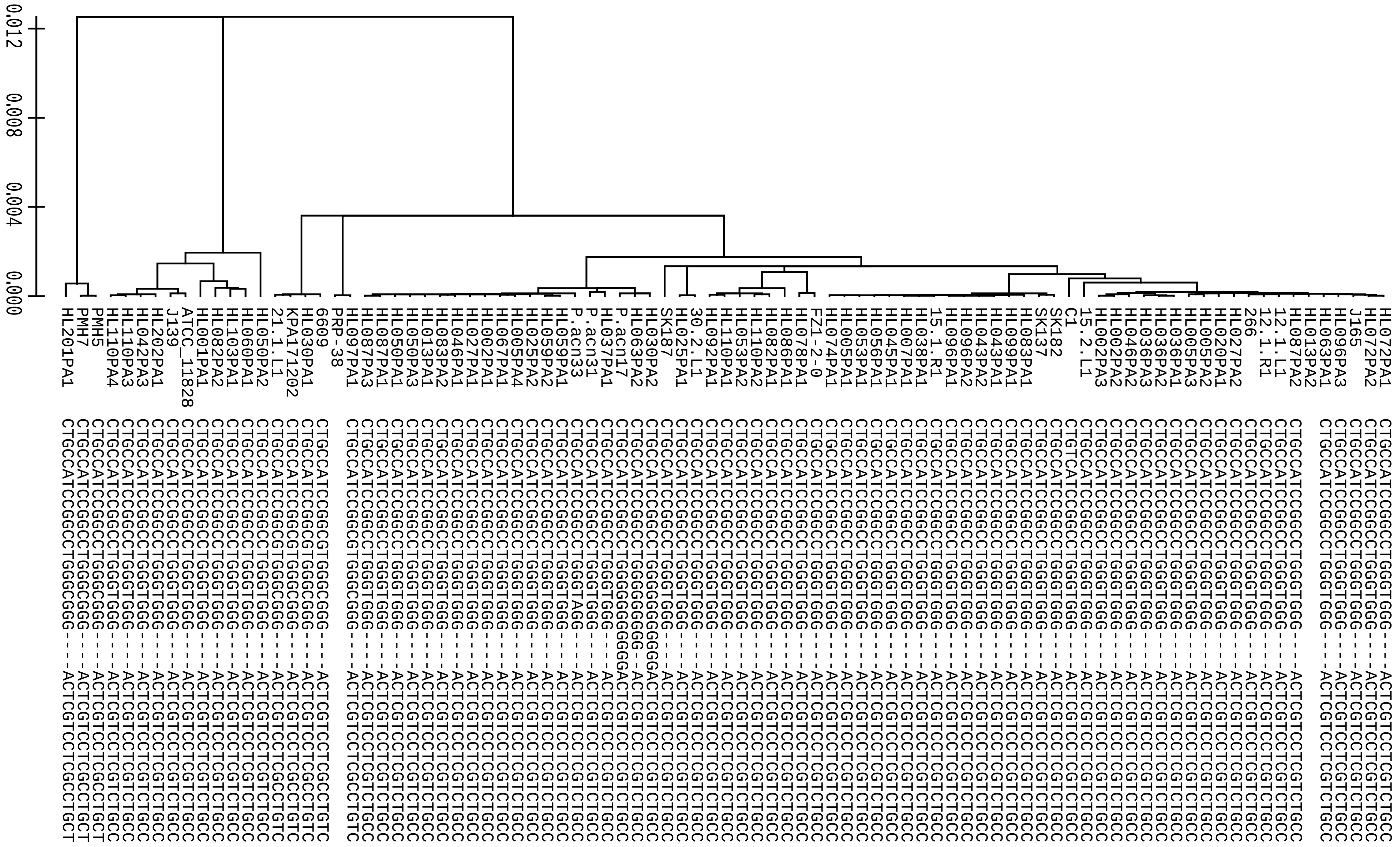

HPT#: HPT32
Gene: Hypothetical Protein
PPA: PPA1874
Location: 2032637:2032658 (KPA171202)
Page: 32/54

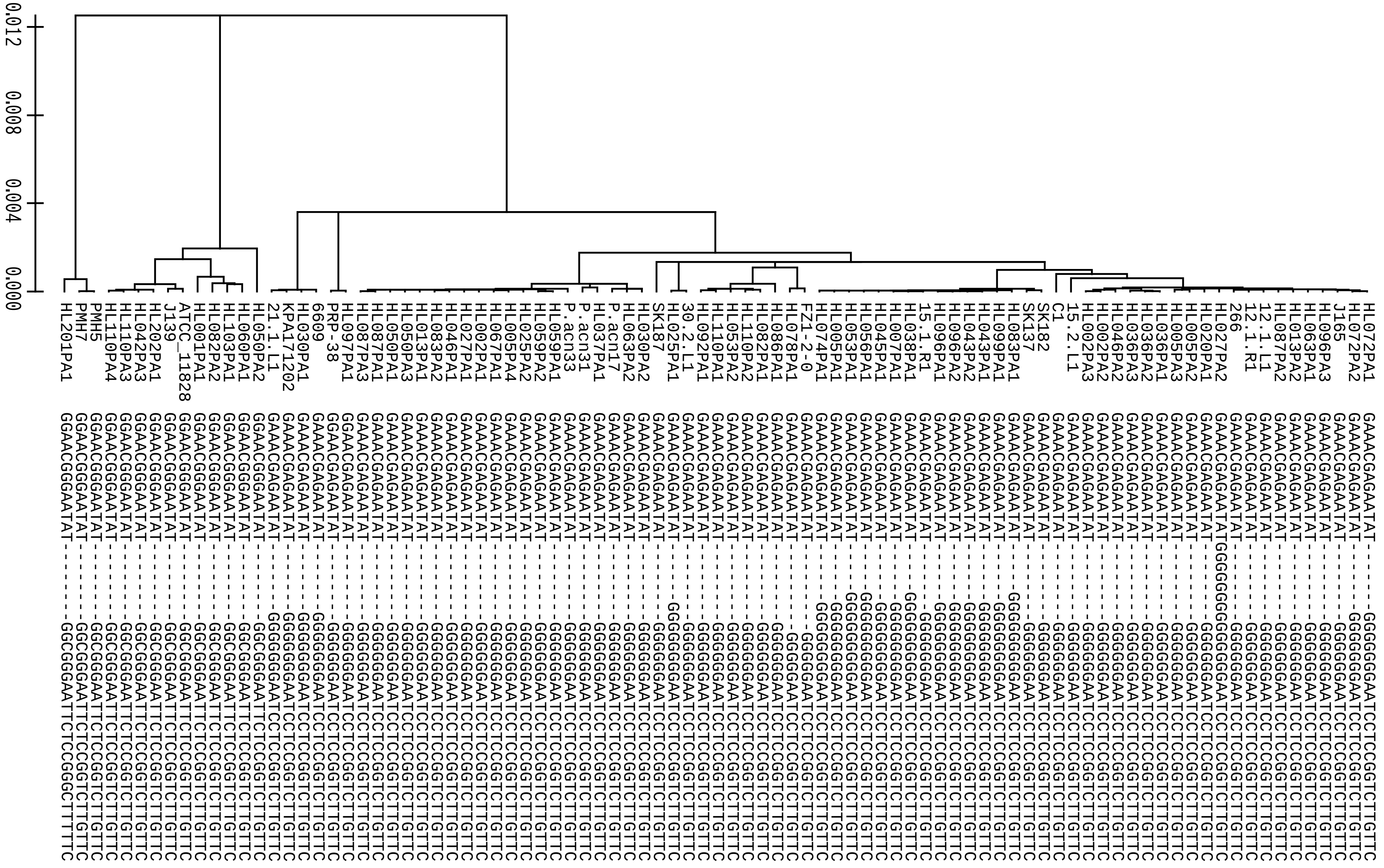

HPT#: HPT33
Gene: Hypothetical Protein
PPA: PPA0089
Location: 2039174:2039199 (KPA171202)
Page: 33/54

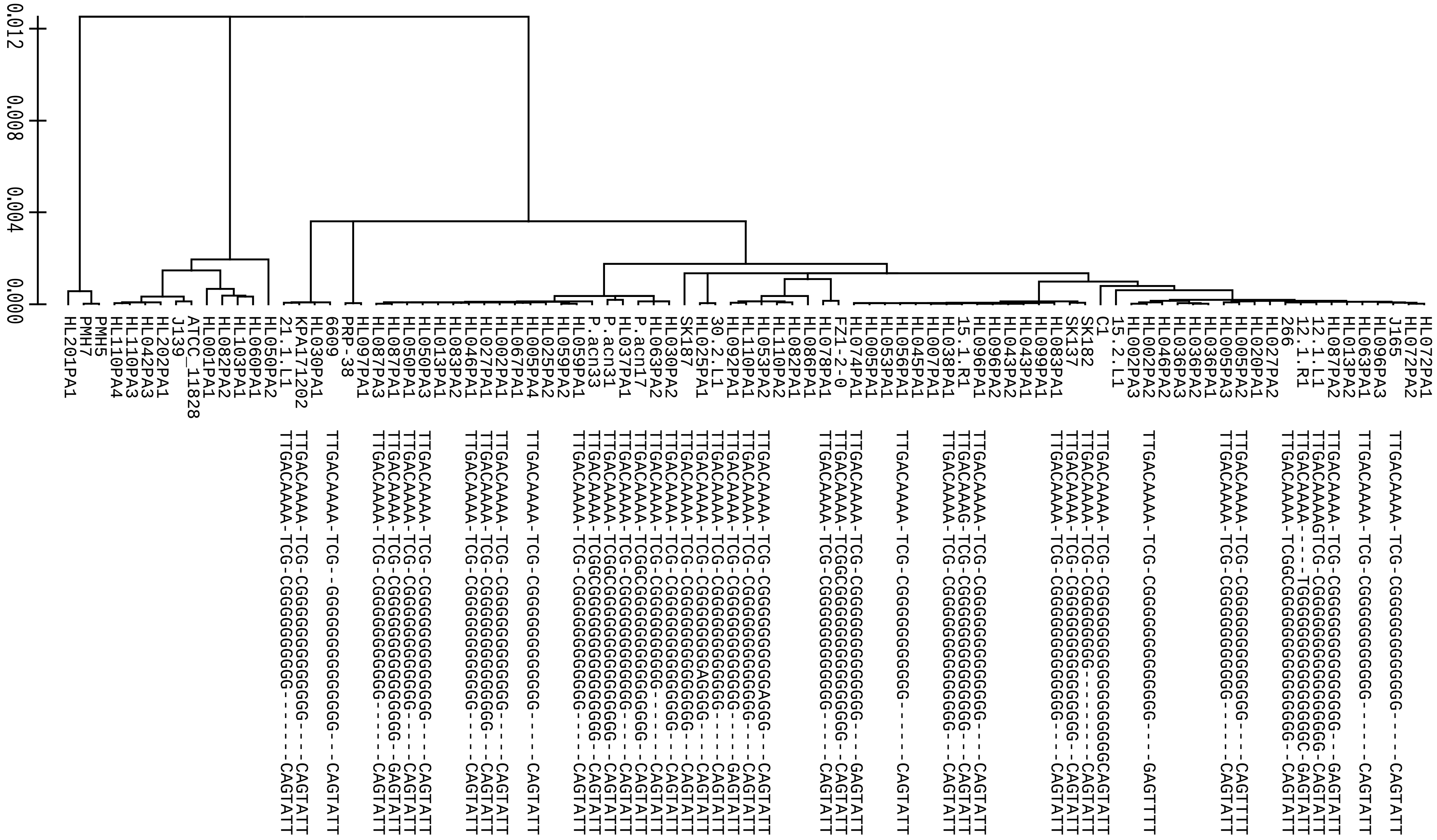

HPT#: HPT34

Gene: 50S ribosomal protein L10

PPA: PPA1887

Location: 2050286:2050309 (KPA171202)

Page: 34/54

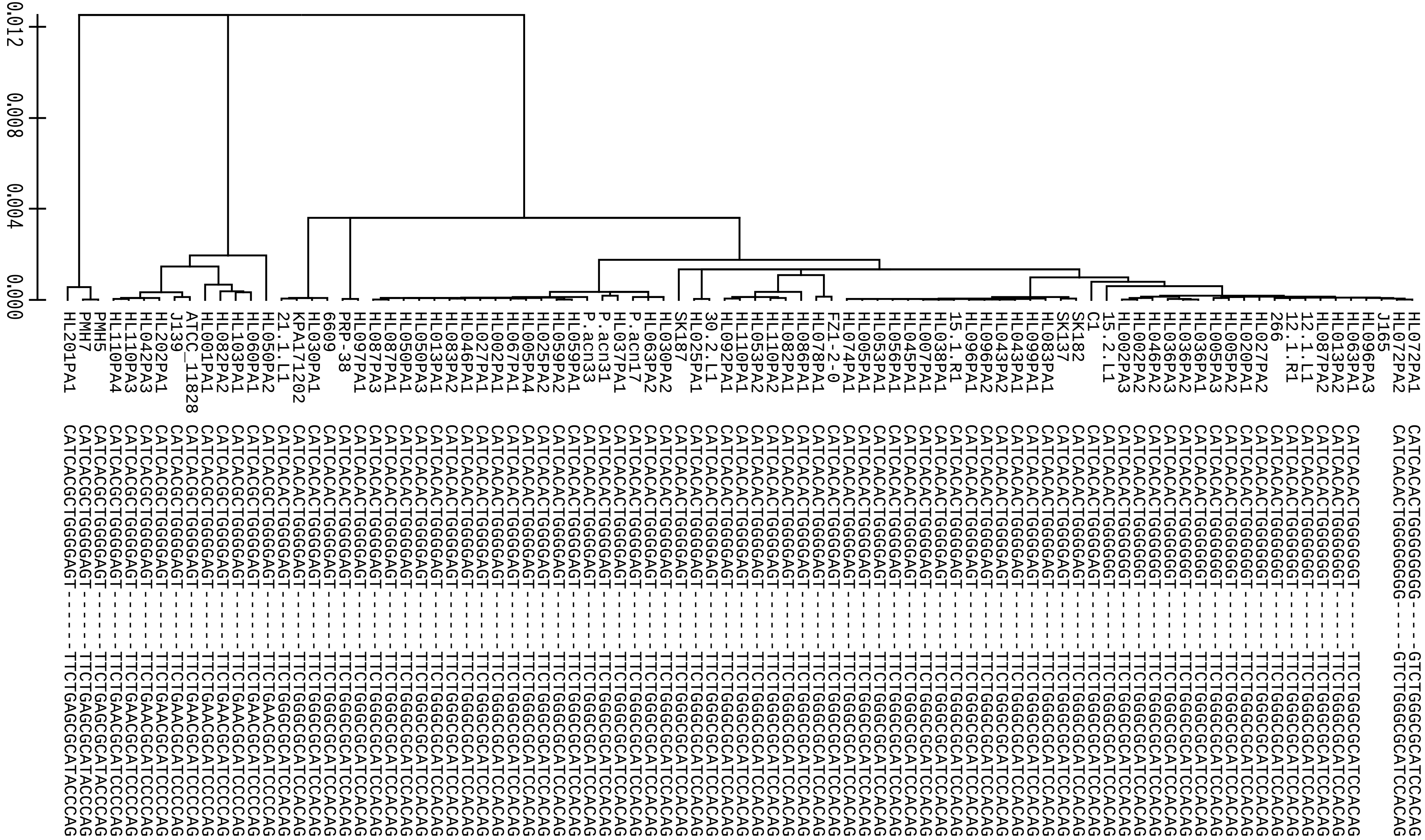

HPT#: HPT35
Gene: Protein associated to adhesion protein
PPA: PPA1907
Location: 2069318:2069346 (KPA171202)
Page: 35/54

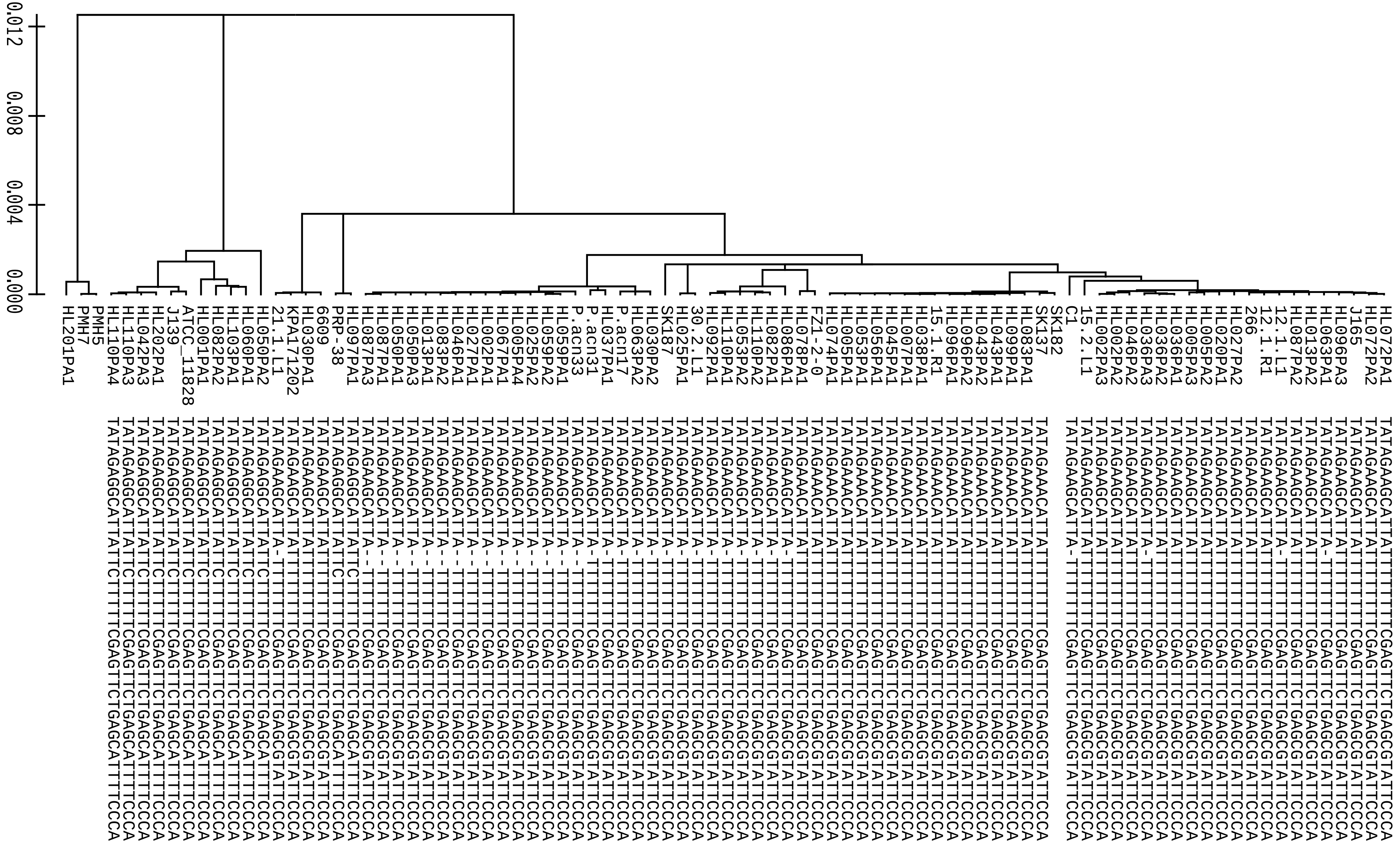

HPT#: HPT36
Gene: ABC Transporter, ATP-binding protein
PPA: PPA1911
Location: 2074490:2074507 (KPA171202)
Page: 36/54

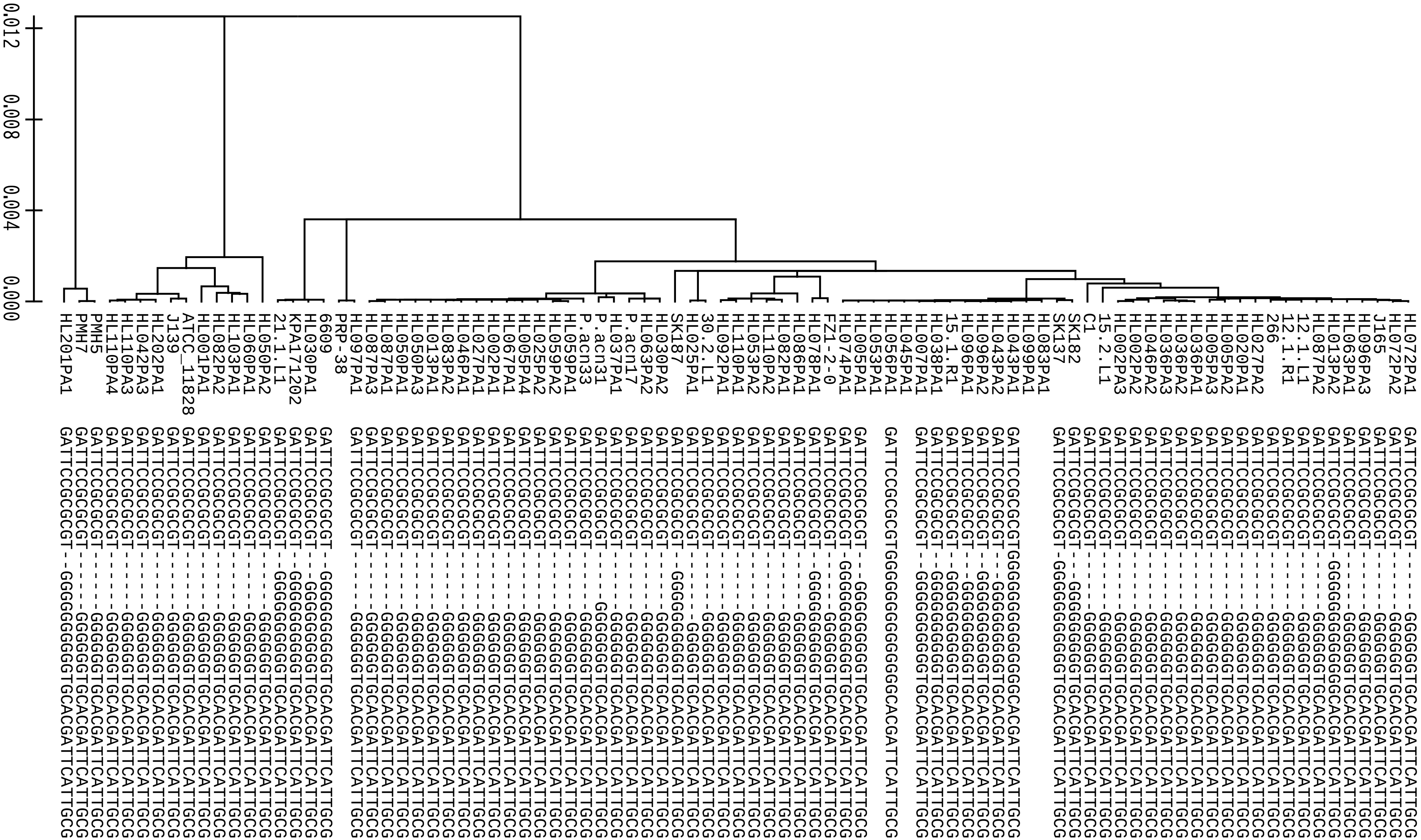

HPT#: HPT37

Gene: ubiquinone/menaquinone biosynthesis methyltransferase UbiE

PPA: PPA1943

Location: 2103894:2103925 (KPA171202)

Page: 37/54

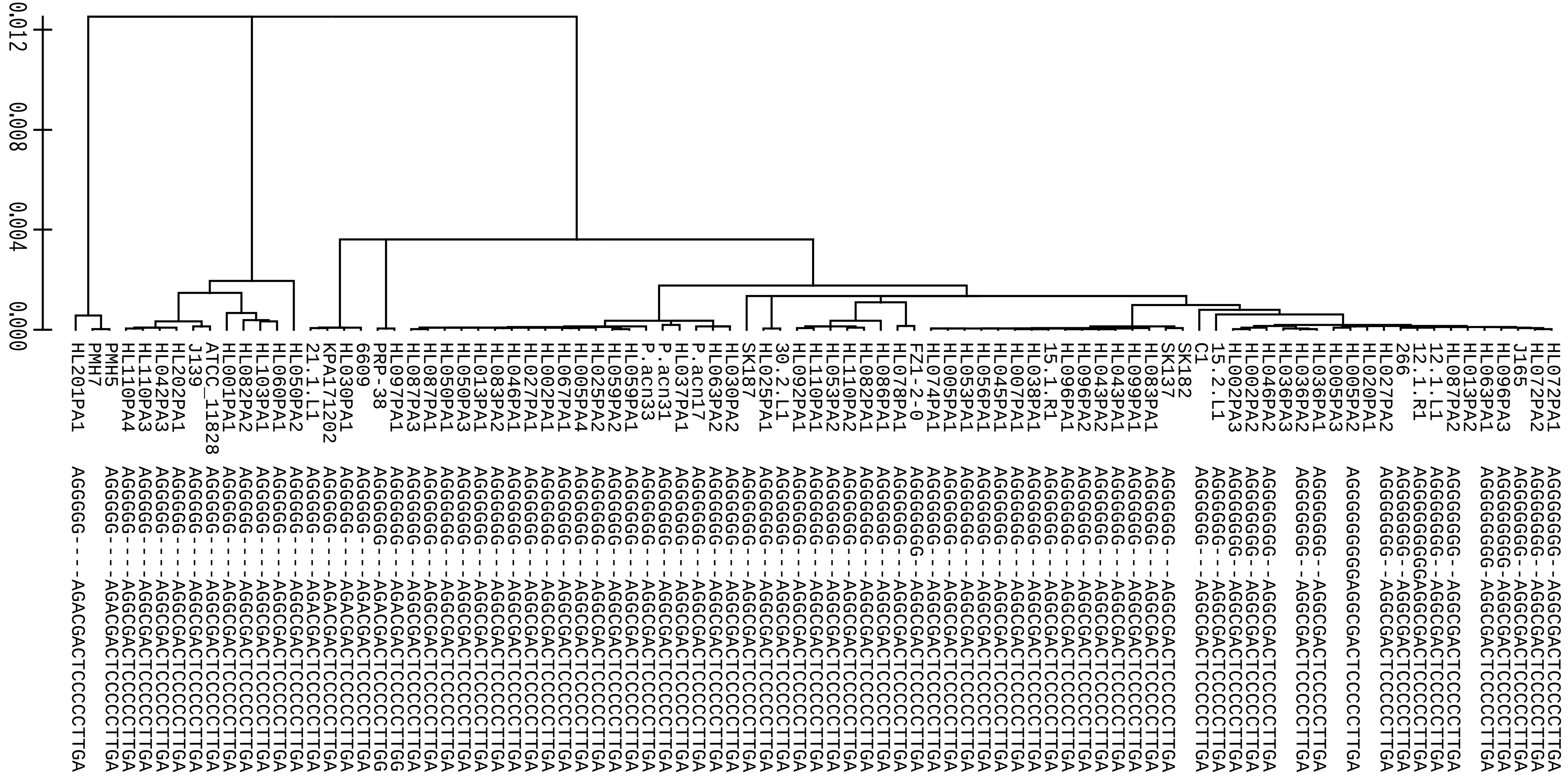

HPT#: HPT38
Gene: Peptidoglycan binding domain
PPA: PPA2004
Location: 2177057:2177094 (KPA171202)
Page: 38/54

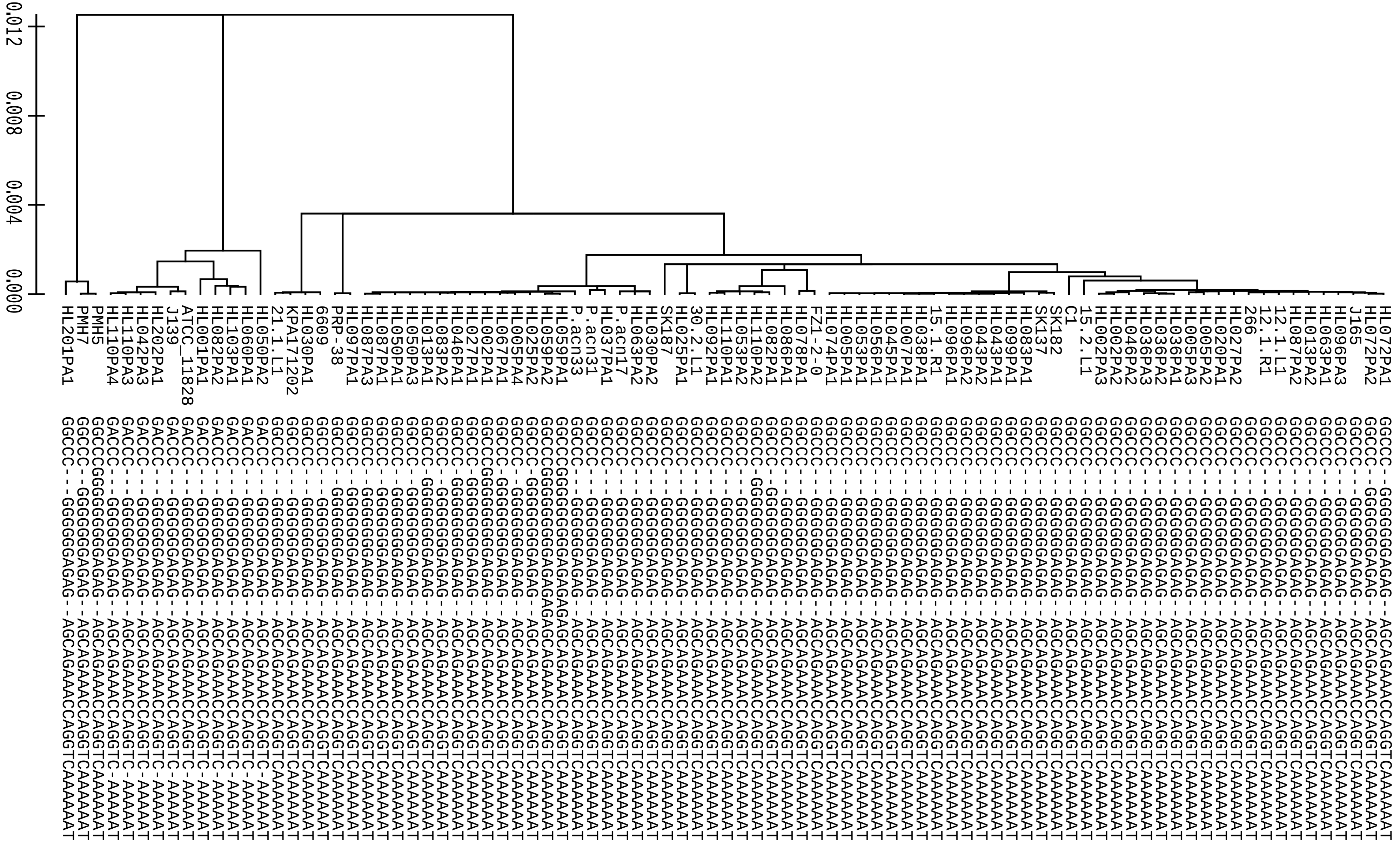

HPT#: HPT39
Gene: Adhesion or S-layer protein
PPA: PPA2127
Location: 2302361:2302390 (KPA171202)
Page: 39/54

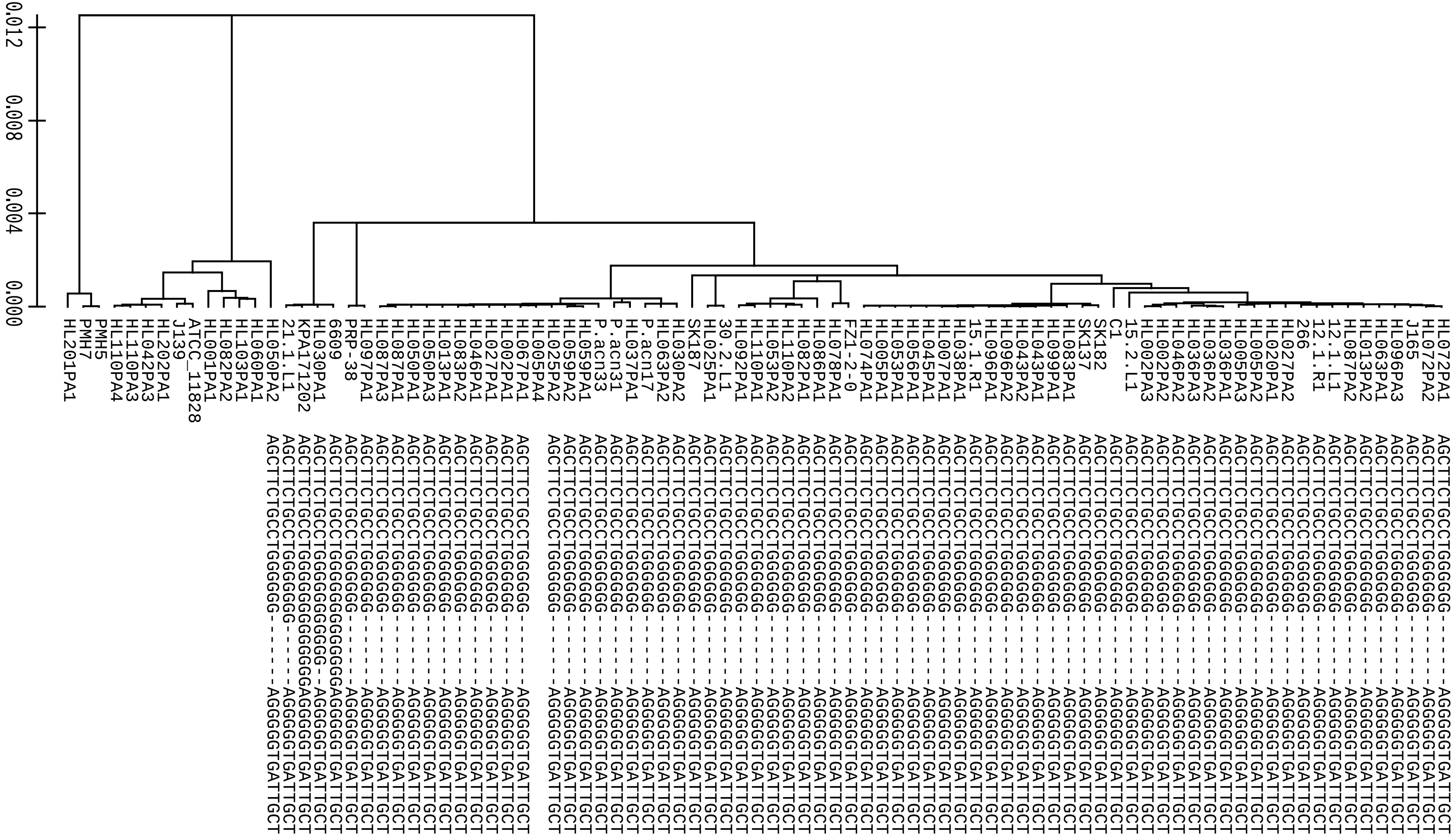

HPT#: HPT40
Gene: Class I glutamine aminotransferase
PPA: PPA2188
Location: 2370041:2370068 (KPA171202)
Page: 40/54

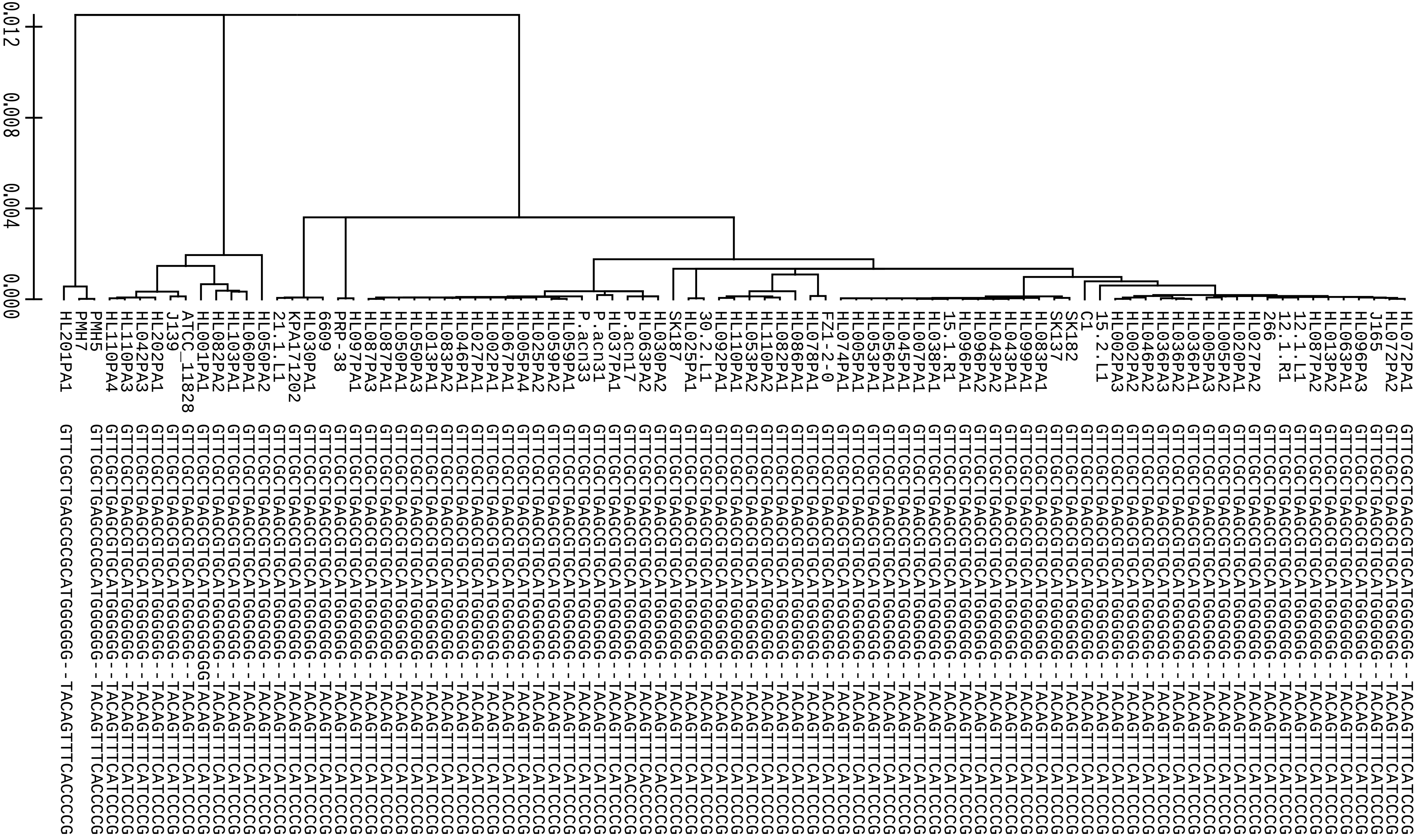

HPT#: HPT41

Gene: Long-chain fatty-acid—CoA ligase/synthetase

PPA: PPA2234

Location: 2419073:2419099 (KPA171202)

Page: 41/54

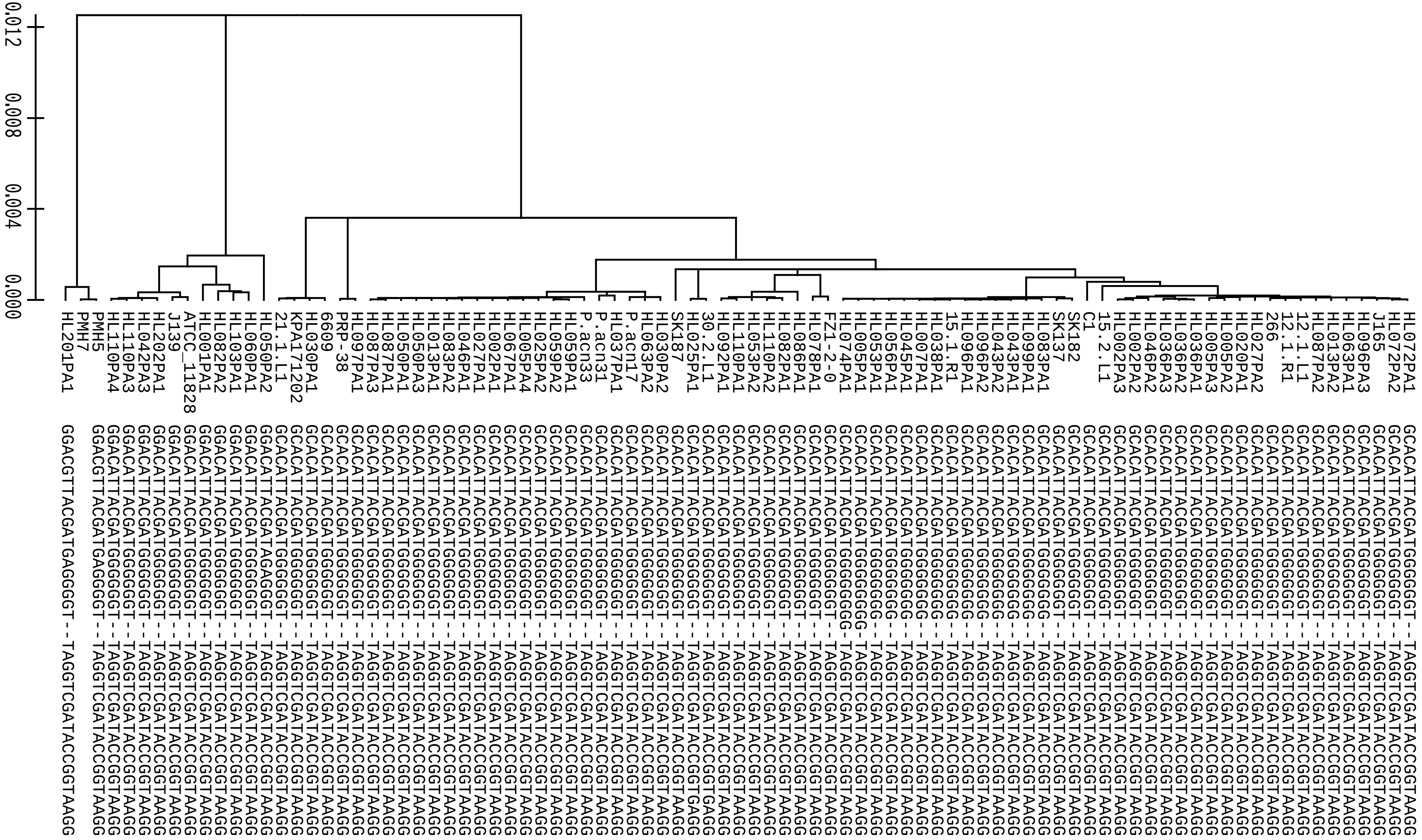

HPT#: HPT42
Gene: 3-methyladenine DNA glycosylase
PPA: PPA2247
Location: 2430915:2430943 (KPA171202)
Page: 42/54

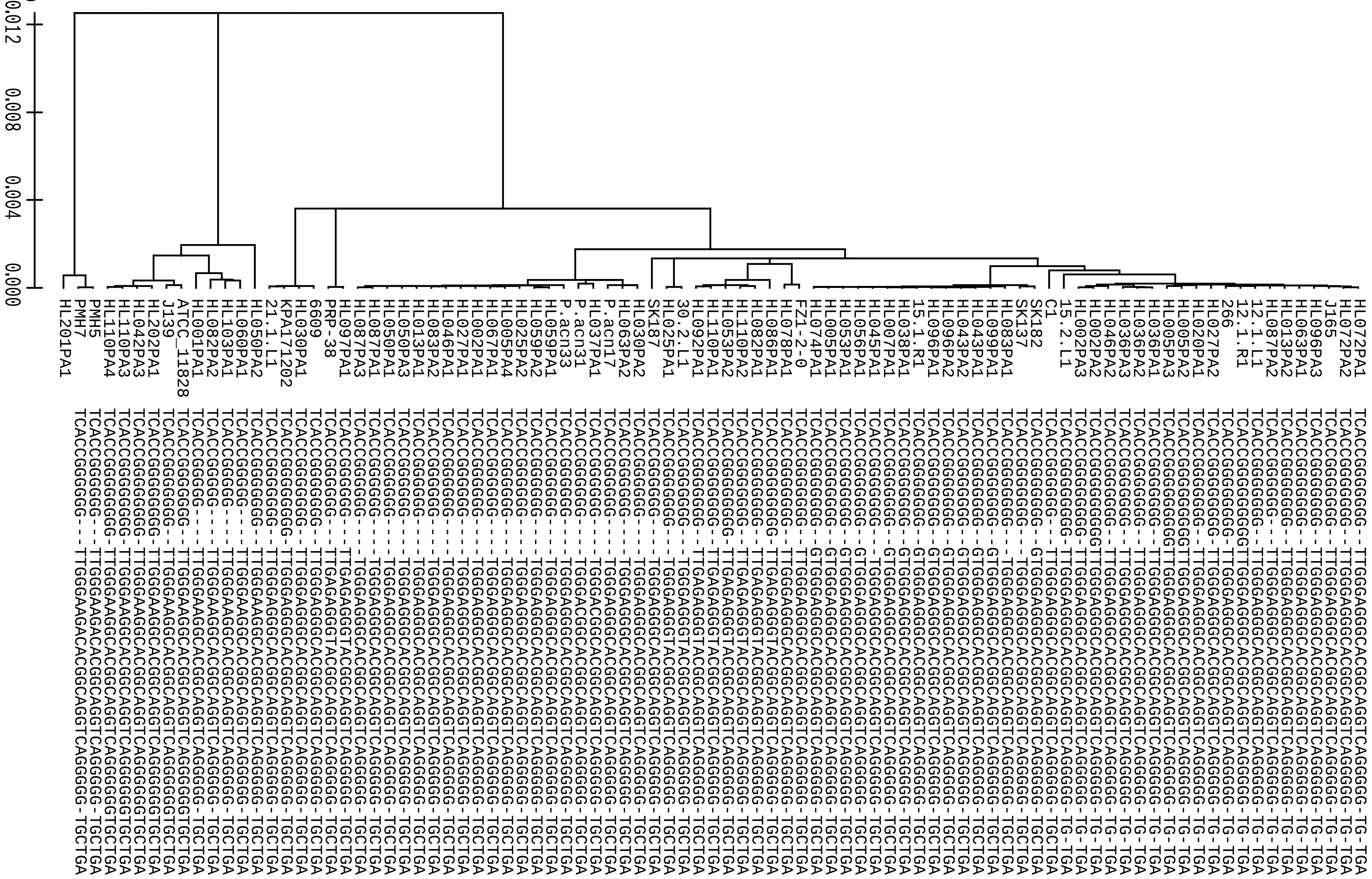

HPT#: HPT43
Gene: Glycerol-3-phosphate dehydrogenase, anaerobic, subunit A
PPA: PPA2250
Location: 2435469:2435495 (KPA171202)
Page: 43/54

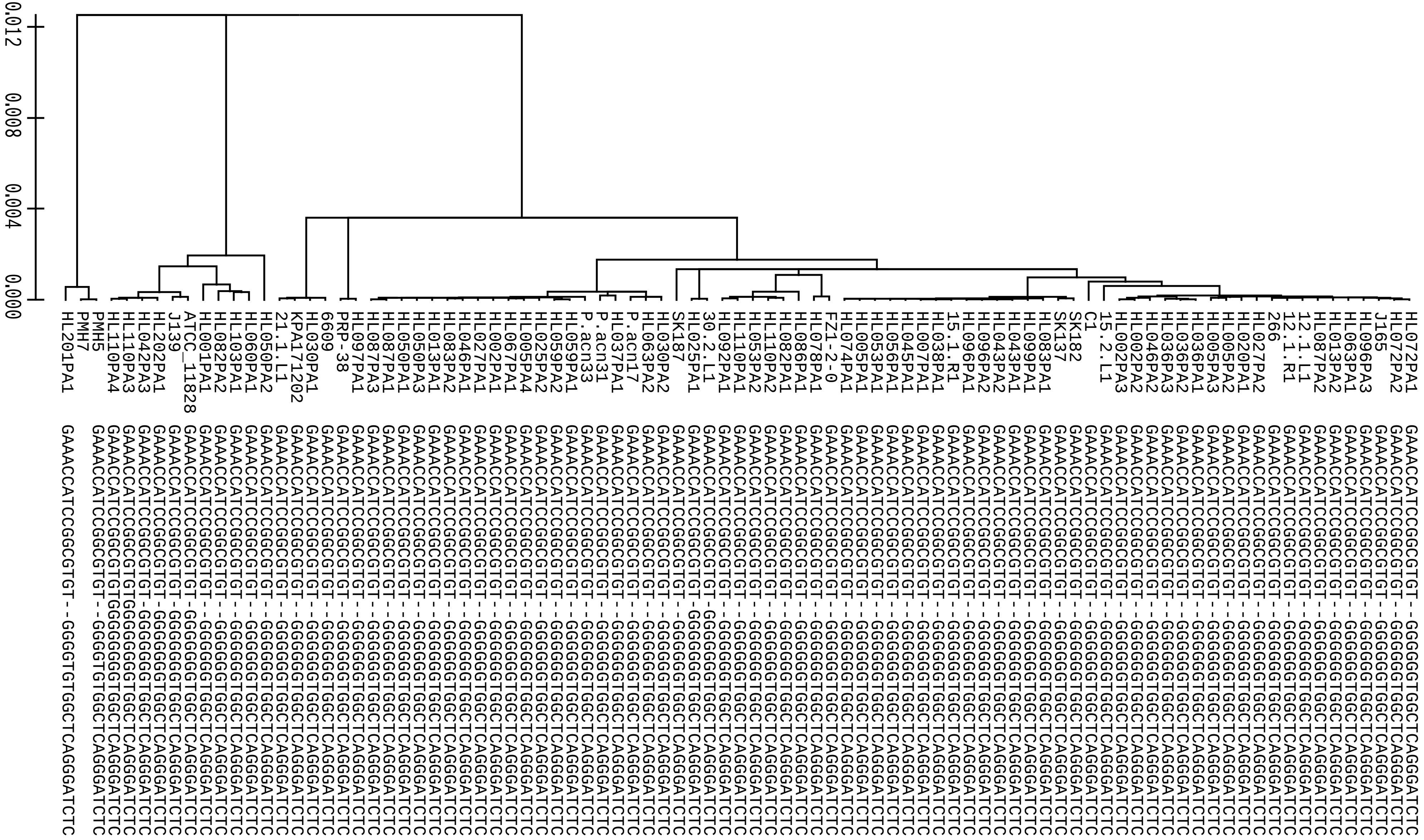

HPT#: HPT44

Gene: Glycerate kinase

PPA: PPA2299

Location: 2494978:2495000 (KPA171202)

Page: 44/54

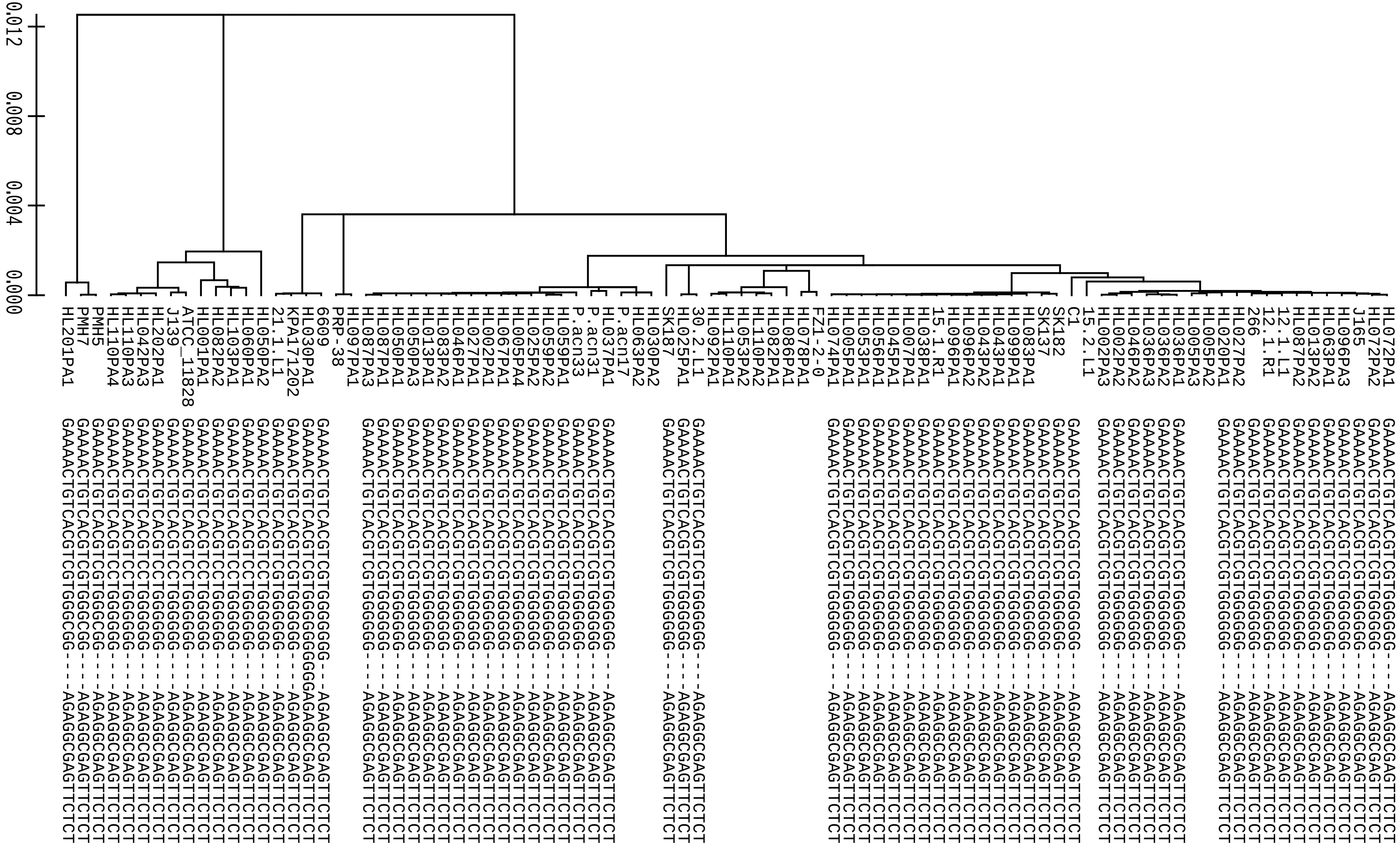

HPT#: HPT45
Gene: Thioredoxin disulfide reductase
PPA: PPA2309
Location: 2509661:2509688 (KPA171202)
Page: 45/54

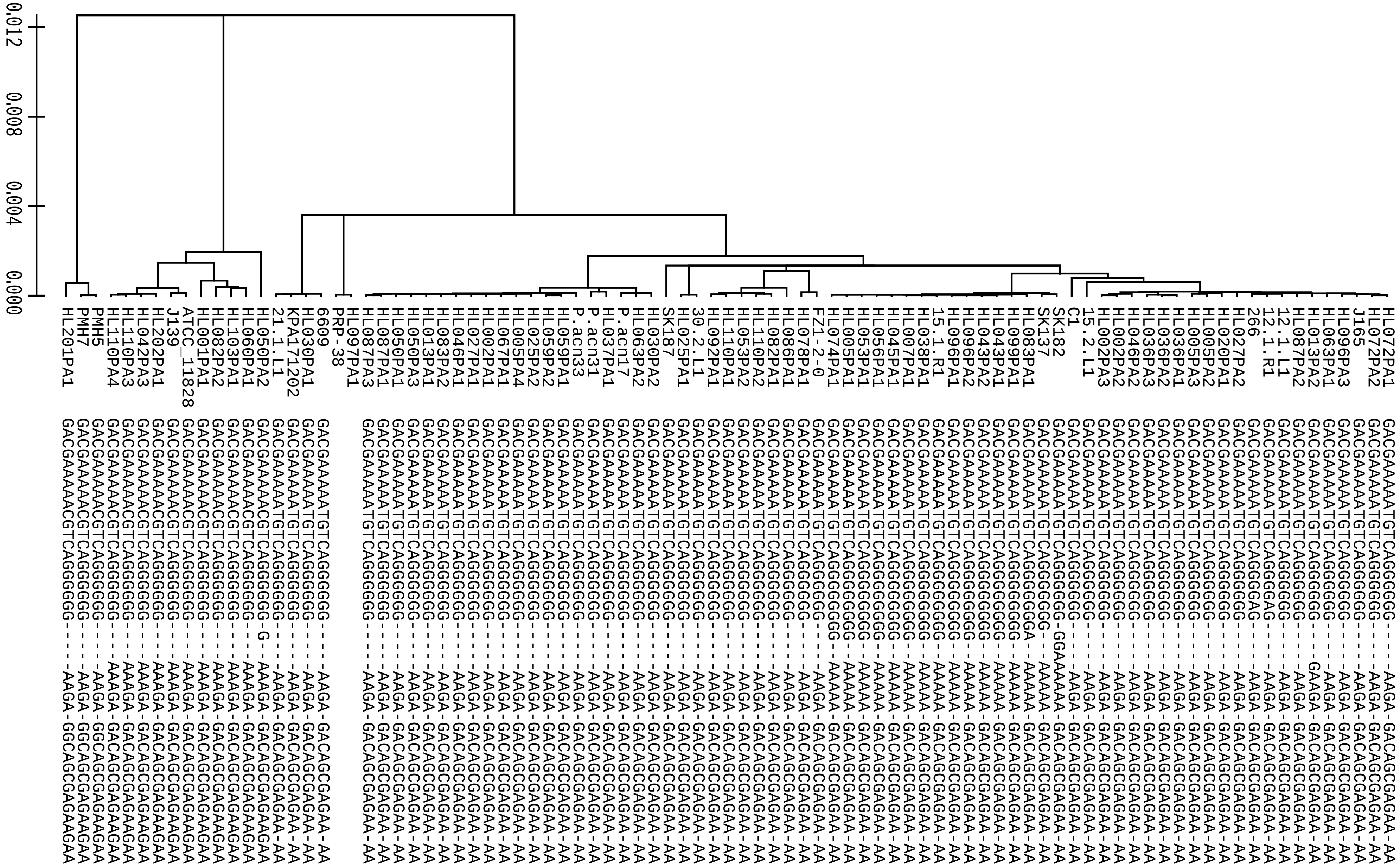

HPT#: HPT46
Gene: Thioredoxin disulfide reductase / alpha-L-arabinofuranosidase
PPA: PPA2308PPA2309
Location: 2508371:2508396 (KPA171202)
Page: 46/54

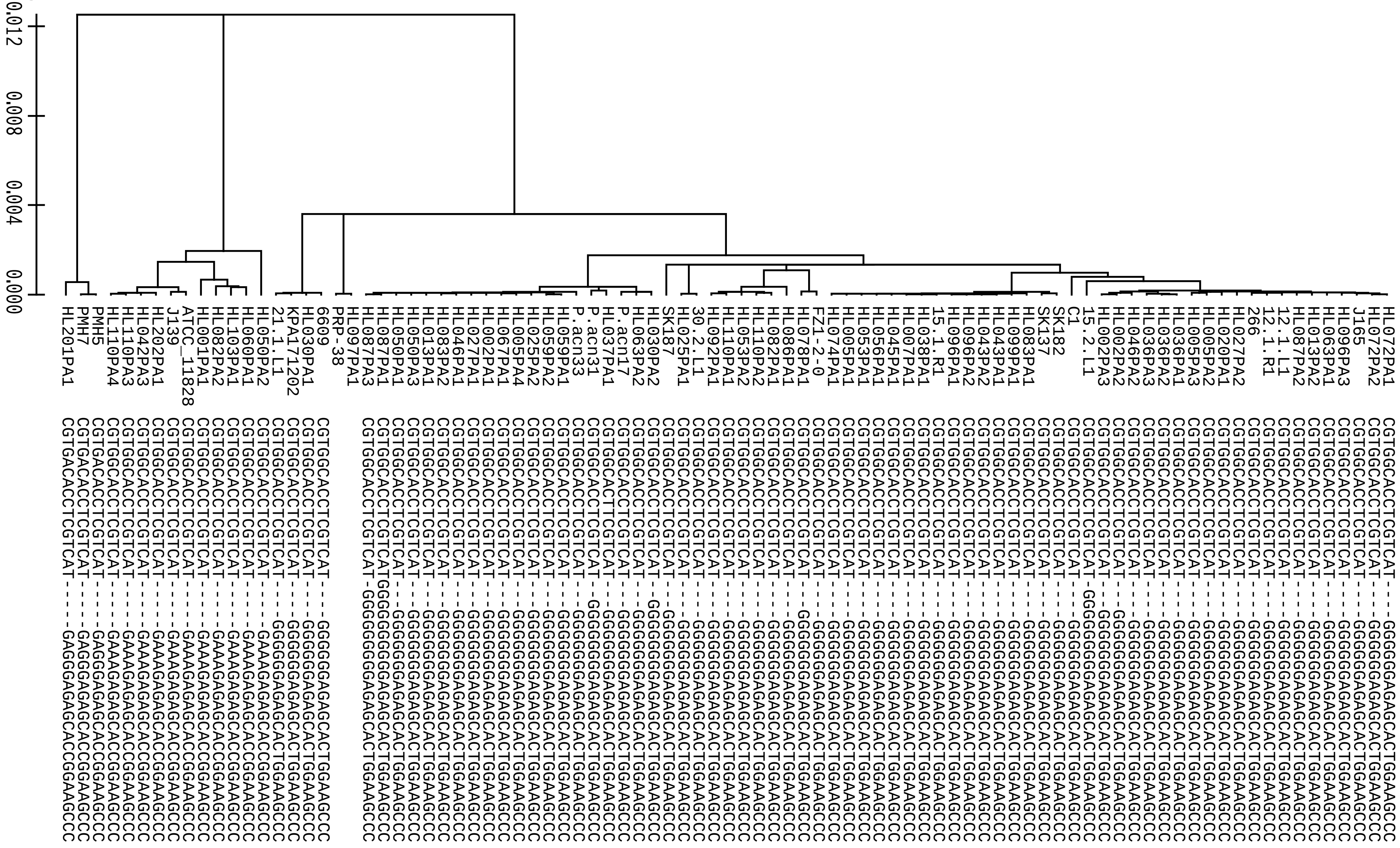

HPT#: HPT47
Gene: Hypothetical Protein
PPA: PPA2379
Location: 1249158:1249187 (KPA171202)
Page: 47/54

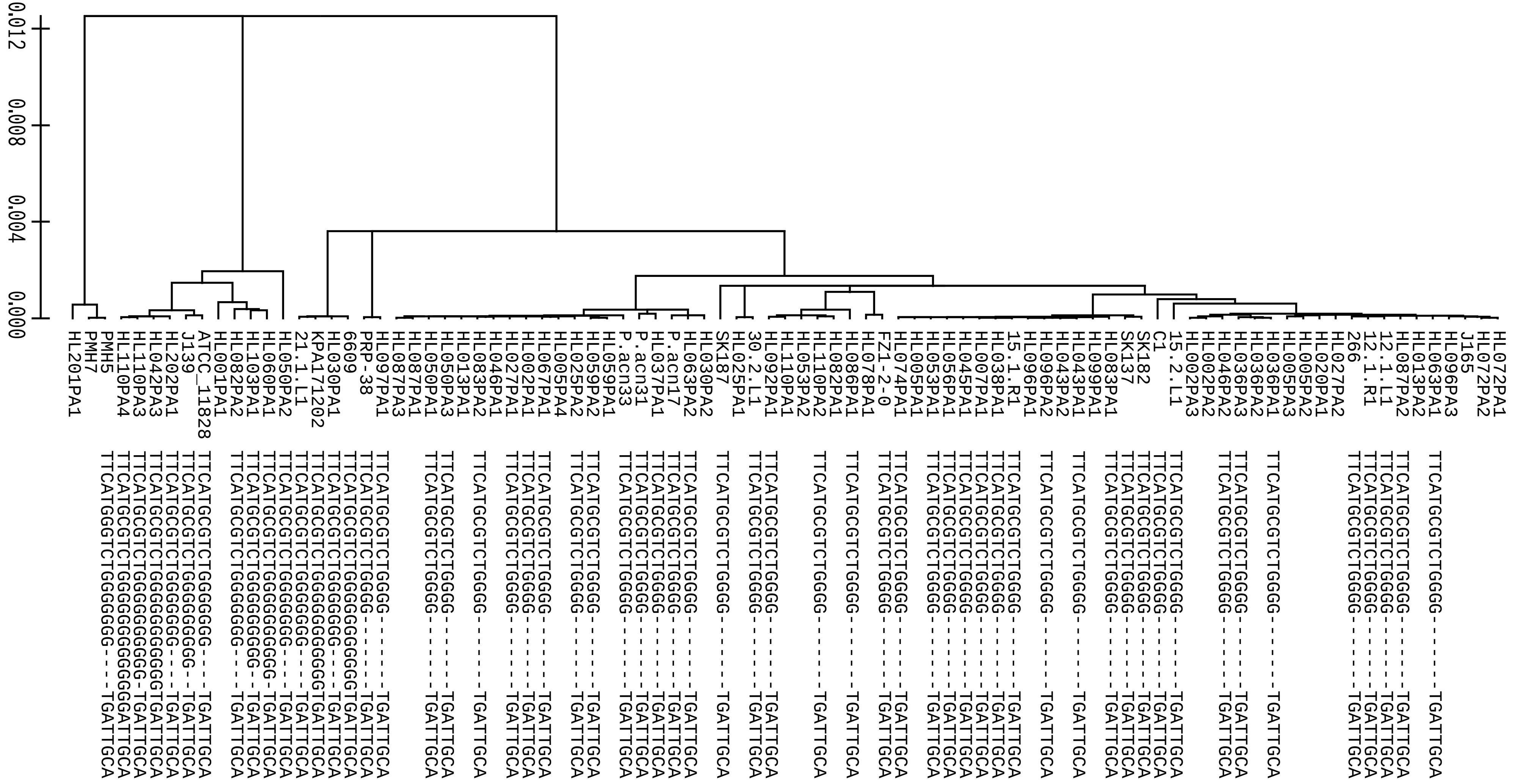

HPT#: HPT48
Gene: Hypothetical Protein
PPA: PPA0089
Location: 31654:31684 (KPA171202)
Page: 48/54

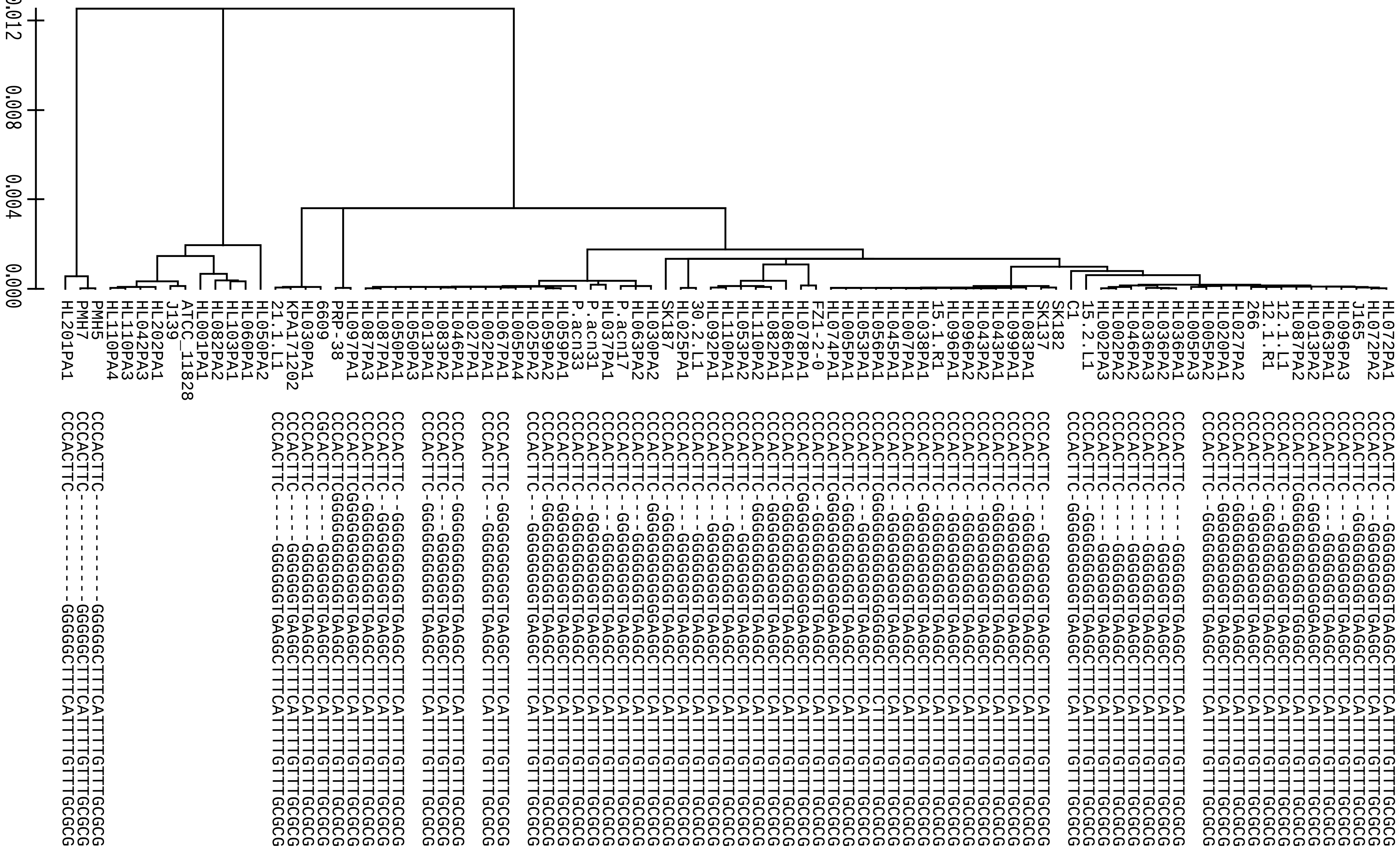

HPT#: HPT49
Gene: Putative sugar transporter
PPA: PPA0292
Location: 345730:345753 (KPA171202)
Page: 49/54

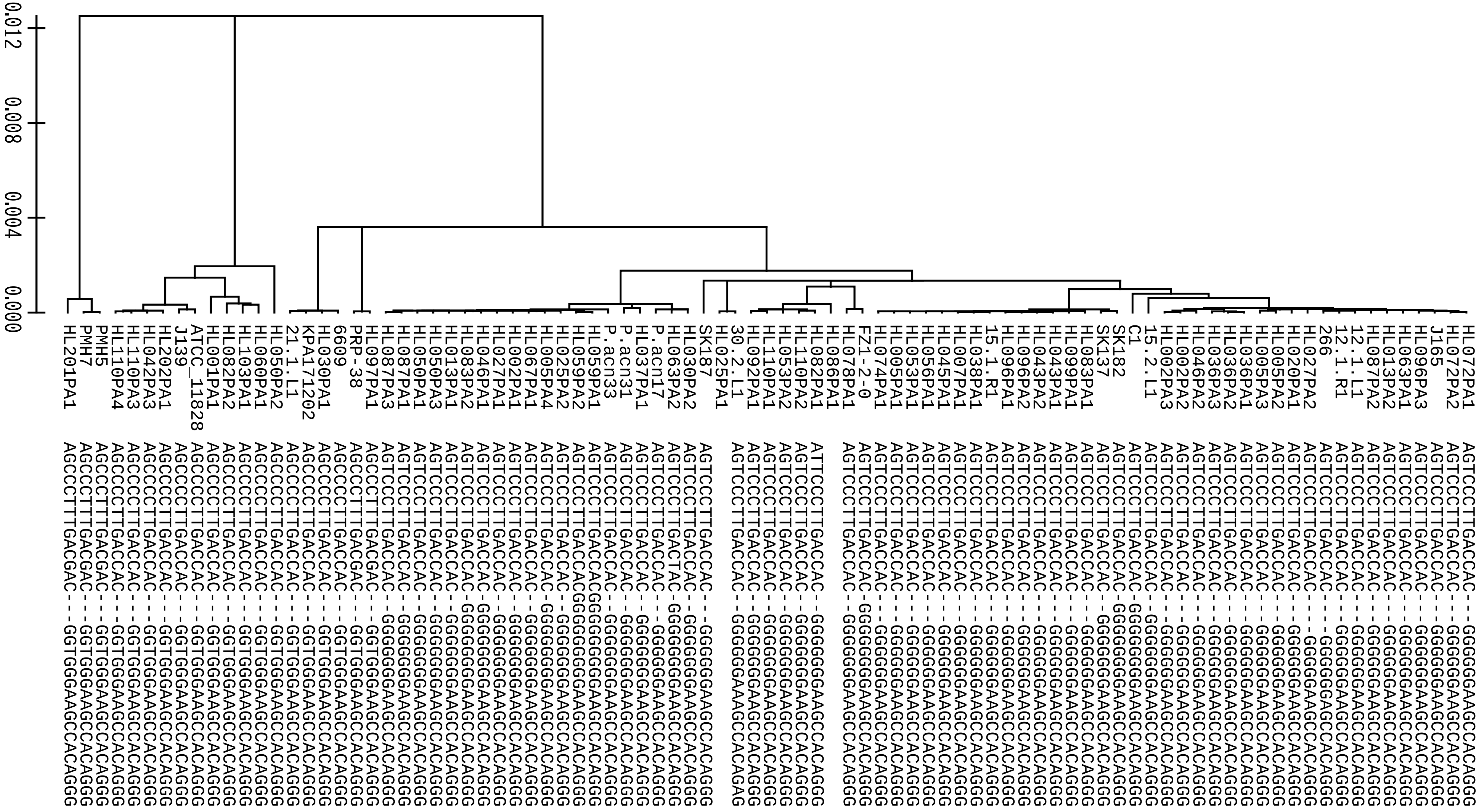

HPT#: HPT50

Type: RNA region, between genes

Location: multiple loci

Page: 50/54

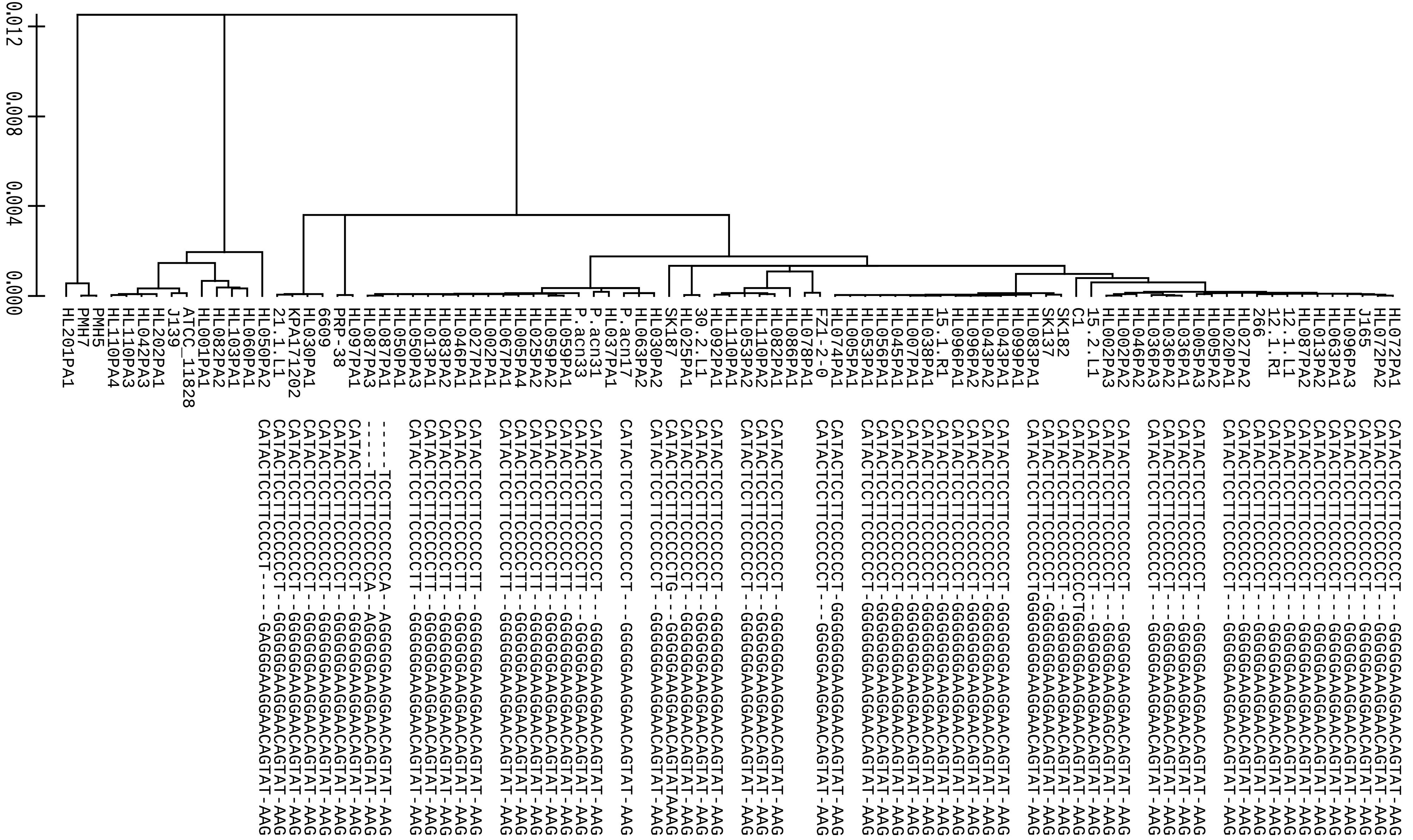

HPT#: HPT51
Location: 1385893:1385915 (KPA171202)
Page: 51/54

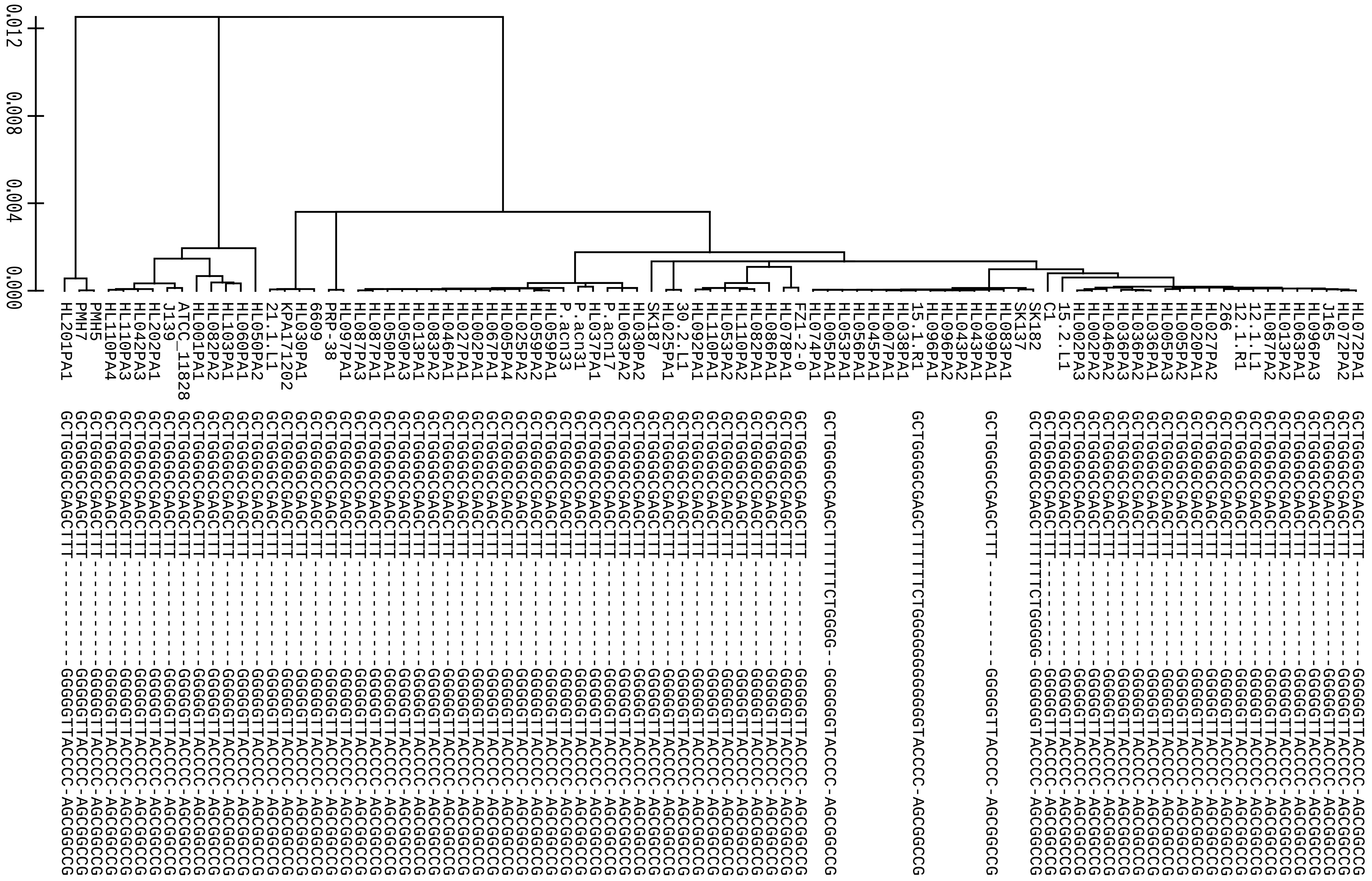

HPT#: HPT52
Gene: Hypothetical Protein
PPA: N/A
Location: 2059397:2059419 (266)
Page: 52/54

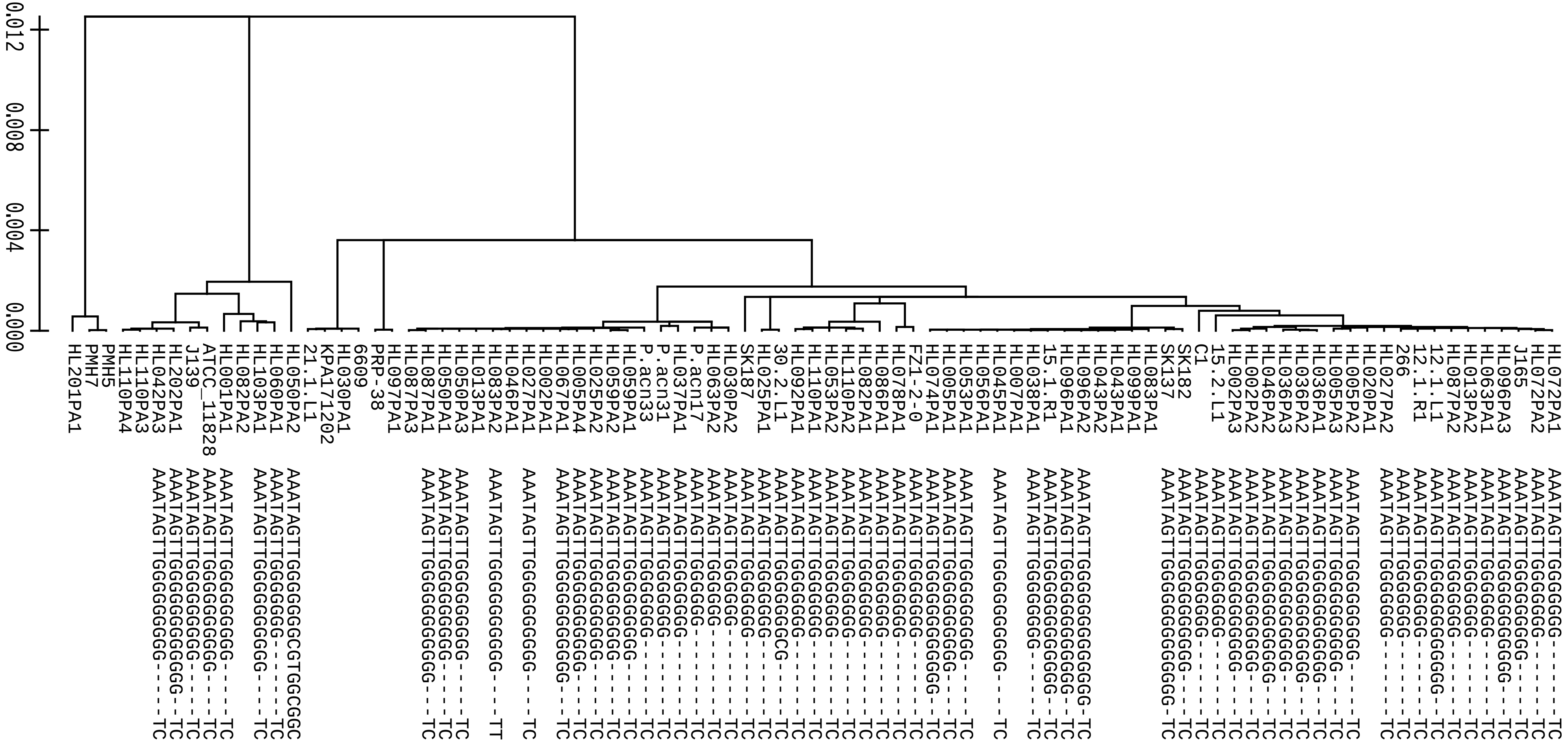

HPT#: HPT53
Gene: N/A
PPA: N/A
Location: 785167:785193 (ATCC 11828)
Page: 53/54

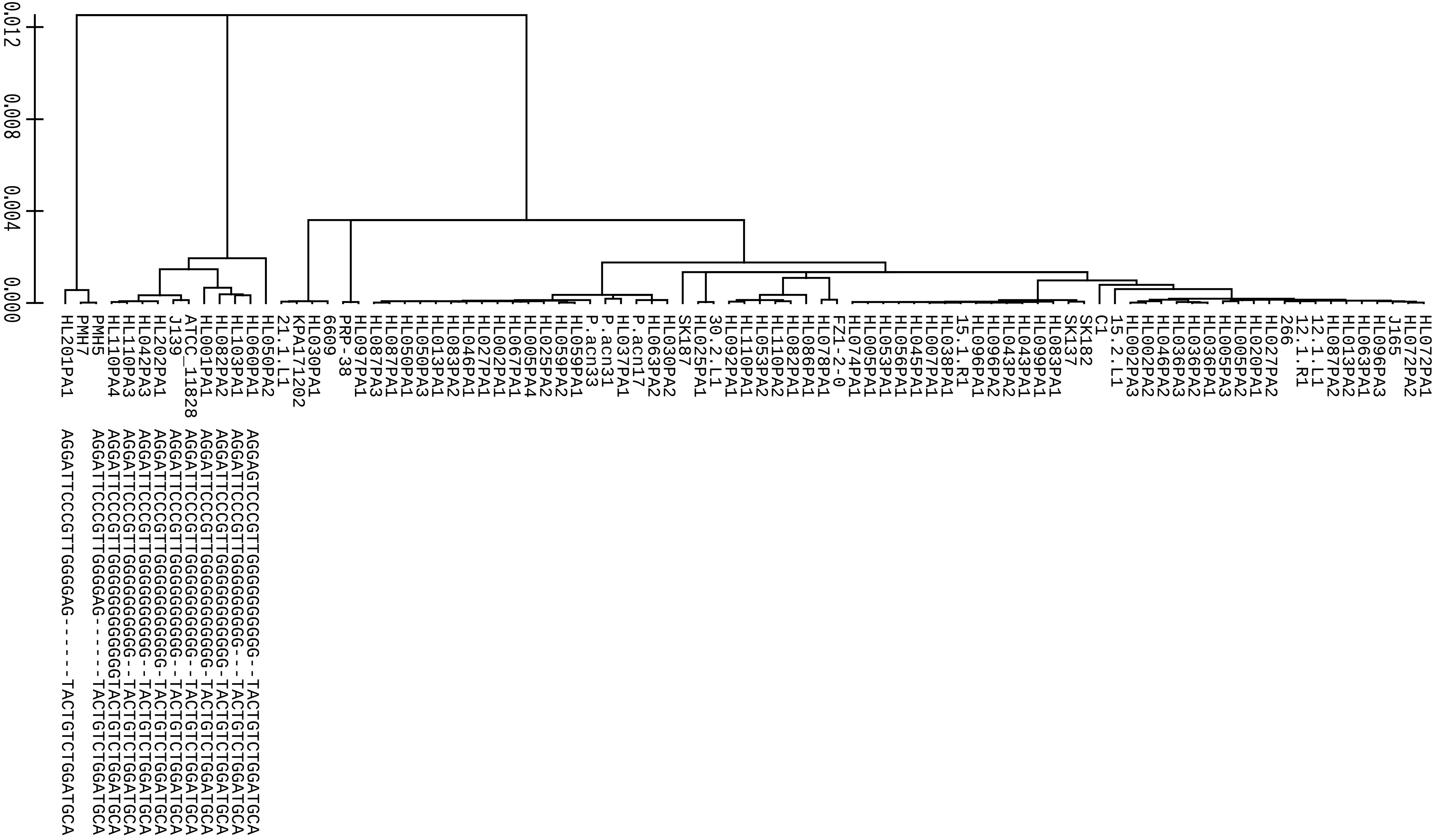

HPT#: HPT54

Gene: Hypothetical Protein

PPA: N/A

Location: 461950:461979 (266)

Page: 54/54

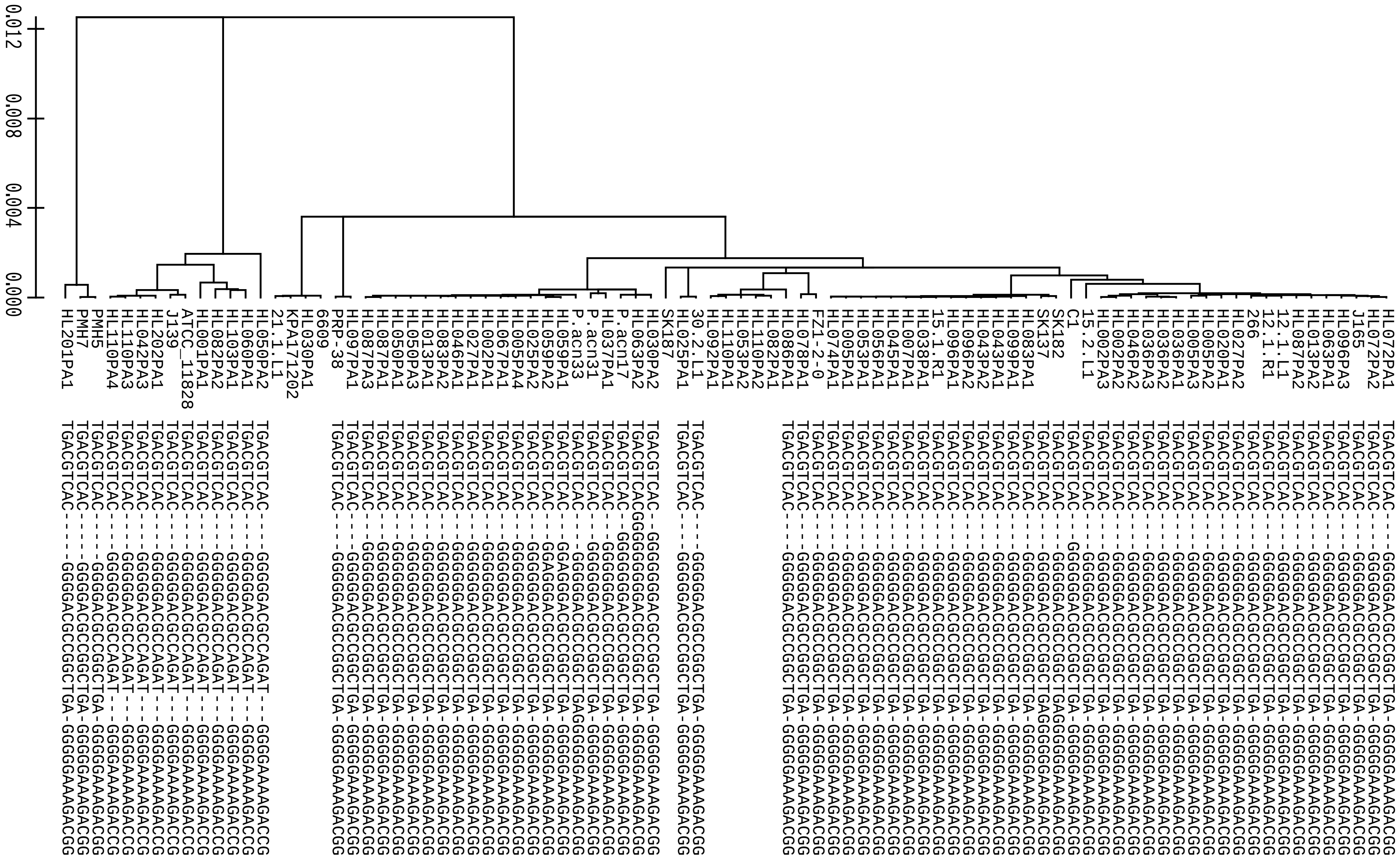

Supplement: Supplementary Information [file srep20662-s1.pdf]
